# Energy Generation and Carbon Footprint under Future Projections (2022–2100) of Central Asian Temperature Extremes

**DOI:** 10.1002/gch2.202400356

**Published:** 2025-03-31

**Authors:** Parya Broomandi, Mehdi Bagheri, Ali Mozhdehi Fard, Aram Fathian, Mohammad Abdoli, Adib Roshani, Sadjad Shafiei, Michael Leuchner, Jong Ryeol Kim

**Affiliations:** ^1^ Department of Civil and Environmental Engineering School of Engineering and Digital Sciences Nazarbayev University Kabanbay Batyr Ave. 53 Astana 010000 Kazakhstan; ^2^ Department of Electrical and Computer Engineering School of Engineering and Digital Sciences Nazarbayev University Kabanbay Batyr Ave. 53 Astana 010000 Kazakhstan; ^3^ Faculty of Civil Engineering Babol Noshirvani University of Technology Babol 484 Iran; ^4^ Neotectonics and Natural Hazards Institute RWTH Aachen University 52056 Aachen Germany; ^5^ UNESCO Chair on Coastal Geo‐Hazard Analysis Research Institute for Earth Sciences Tehran 13185‐1494 Iran; ^6^ Water, Sediment, Hazards, and Earth‐surface Dynamics (waterSHED) Lab Department of Geoscience University of Calgary Calgary Alberta T2N 1N4 Canada; ^7^ Physical Geography and Climatology Department of Geography RWTH Aachen University Wüllnerstr. 5b 52062 Aachen Germany

**Keywords:** CO_2_ emissions, cooling‐heating degree days, energy demand, fossil‐fueled development scenario, middle‐of‐the‐road scenario

## Abstract

Limiting the global temperature rise to 1.5 °C is becoming increasingly difficult. The study analyzed data from 700 locations (1962–2100) to assess climate change impacts on heating‐cooling energy and carbon footprint in under‐researched Central Asia (CA). Under SSP2‐4.5, icing and frost days reduce, while summer days and tropical nights increase. Central Asian countries will see an increase in cooling needs despite the projected decline in heating demands, with Kyrgyzstan experiencing the highest rise in cooling degree days, projected to increase by 132% and 165% in the near‐future under SSP2‐4.5 and SSP5‐8.5, respectively. As a result, cooling energy generation is expected to rise by 39% and 92% under SSP2‐4.5 and SSP5‐8.5, respectively. However, CO_2_ emissions for cooling are much lower in Kyrgyzstan and Tajikistan due to their reliance on renewable energy. CO_2_ emissions in these countries are projected to be ≈10 times lower than in other parts of CA. From 2022 to 2100, cooling‐related emissions are estimated to increase by 41% and 80% under SSP2‐4.5 and SSP5‐8.5, respectively across CA. Urgent adaptation is needed for resilient cities and stable power by expanding renewables, modernizing infrastructure, boosting efficiency, adopting policies, and fostering cooperation.

## Graphical abstract

This study examines climate change impacts on heating‐cooling demand and carbon footprint in Central Asia (CA). As icing and frost days decrease, cooling needs will rise, especially in Kyrgyzstan. However, Kyrgyzstan and Tajikistan maintain lower CO_2_ emissions due to renewables. Cooling‐related emissions in CA may rise by 41%–80%, highlighting the need for renewable expansion, infrastructure upgrades, and regional cooperation.

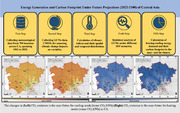

## 1 Introduction

Climate change significantly impacts the energy sector, crucial for developing strategies to address global warming.^[^ ^1^, ^2^, ^3^, ^4^ ^]^ Addressing climate change involves two essential approaches: mitigation and adaptation. Mitigation requires urgent actions such as decarbonizing the electricity grid and reducing per capita energy consumption, given that electricity and heat generation represent the largest source of global greenhouse gas (GHG) emissions^[^ ^5^ ^]^ The integration of advanced technologies and innovative energy materials further complements these strategies, enhancing energy efficiency and sustainability in the long‐term.^[^ ^6^, ^7^, ^8^ ^]^ Scenario‐based approaches for modern power systems, especially those with a high share of renewable energy, are critical for achieving low‐carbon energy transitions while ensuring reliability and grid flexibility.^[^ ^9^ ^]^ Adaptation strategies emphasize ensuring a reliable energy supply to support critical functions like food production, water treatment, and climate acclimatization, which are vital for economic resilience.^[^ ^10^ ^]^ Balancing energy supply with the expected increase in global energy consumption has become a critical concern for policymakers.

In the context of global warming, anthropogenic GHG emissions, particularly from energy production, have reached record levels, driving substantial shifts in climate patterns. Studies highlight that CO_2_ alone accounts for ≈64% of total GHG radiative forcing, making the energy sector a central focus for mitigation strategies.^[^ ^11^ ^]^ Trends in extreme weather events and rising global temperatures underscore the urgency of reducing fossil fuel reliance and expanding renewable energy sources to achieve net‐zero emissions. Simultaneously, adaptation policies must ensure energy security amidst growing demand and evolving climate patterns.^[^ ^12^ ^]^

Electricity grid planners must consider anticipated shifts in electricity demand influenced by various factors. Socio‐economic and meteorological conditions significantly impact electricity demand over different timescales, with socio‐economic factors influencing long‐term variability and daily weather conditions affecting day‐to‐day demand.^[^ ^13^, ^14^, ^15^, ^16^, ^17^ ^]^ Meteorological factors like increased cloud cover can increase lighting demands, variations in relative humidity can impact air conditioning efficiency, and wind speed can affect heat transmission through buildings.^[^ ^10^ ^]^ Temperature is a primary meteorological driver, influencing electricity demand in a nonlinear fashion across various socio‐economic contexts.^[^ ^18^, ^19^ ^]^ Extreme temperatures drive up electricity usage due to increased heating‐cooling needs, measured using weather‐based indices like heating degree days (HDDs) and cooling degree days (CDDs), reflecting directly on energy consumption patterns.^[^ ^20^, ^21^ ^]^

Reflecting on the established relationship between demand and temperature, and considering the ongoing trends in global warming, climate change is anticipated to significantly reshape regional electricity demand patterns. Studies consistently forecast a decrease in HDDs and an increase in CDDs across various regions, suggesting rising cooling demand and declining heating needs.^[^ ^3^, ^20^, ^21^, ^22^ ^]^ This overall shift in electricity demand will largely depend on regional variations in temperature changes. Studies on the influence of climate change on energy usage in various regions underscore the extensive scholarly attention to this issue.

In Brazil, weather variables were found to significantly influence electricity demand trajectories from 2016 to 2100.^[^ ^23^ ^]^ In the US, an increase in the intensity and frequency of peak demand events is forecasted, posing challenges such as energy shortages and higher electricity costs.^[^ ^19^, ^24^ ^]^ In Europe, future warming's net impact on electricity consumption is expected to be negligible due to a north‐south polarization, with northern Europe seeing a decline and southern and western parts experiencing an increase in demand.^[^ ^10^, ^19^, ^25^ ^]^ Minimal Fluctuations in the average yearly demand are attributed to seasonal adjustments, with reductions in autumn, spring, and winter offset by summer increases.^[^ ^26^ ^]^ The annual peak burden in numerous European countries may transition from winter to summer by the century's end, highlighting the challenges for energy policy and infrastructure adaptation.^[^ ^27^, ^28^ ^]^

In CA, research on the experienced and projected impacts of climate change is limited mainly to mean precipitation and temperature changes. Temperature changes exhibit a uniform pattern, while precipitation variations are heterogeneous.^[^ ^29^, ^30^, ^31^, ^32^, ^33^ ^]^ An insufficient understanding of climate change impacts may lead to heightened economic, environmental, and human tolls. Addressing these knowledge gaps is crucial for effective risk mitigation. Long‐term low‐emission development strategies (LEDS), like those highlighted in global case studies, offer an essential framework for reducing emissions while ensuring energy security and resilience in CA.^[^ ^34^ ^]^ Given the projected increase in extreme temperature events, integrating LEDS with adaptation measures is crucial to building regional energy resilience. This integration can support the development of sustainable energy systems while simultaneously addressing the growing risks posed by climate variability and extreme weather patterns.

Comprehensive assessments of climate indicators—such as temperature extremes, sea level rise, and variations in surface humidity—are crucial for understanding these risks. A detailed analysis of heating‐cooling energy requirements, coupled with multi‐CMIP6 General Circulation Model (GCM) ensemble predictions, provides an enhanced perspective on the region's future energy landscape.^[^ ^12^ ^]^

By concentrating on CA, this study provides new insights into an underexplored area by incorporating multi‐CMIP6 GCMs ensemble predictions and combining analysis of temperature extremes with changes in energy requirements to offer a comprehensive perspective on the influence of climate on energy consumption and CO_2_ emissions. This research study will:

1. Analyze the evolving patterns in warm and cold days and nights to understand shifting temperature extremes.
2. Examine changes in frost‐ice‐summer days, and tropical nights to evaluate dynamic climate conditions.
3. Investigate fluctuations in heating and cooling degree days to comprehend changing energy requirements for climate regulation.
4. Evaluate the impact of changing climate patterns on heating‐cooling energy demands and analyze resulting CO_2_ emission fluctuations from energy production.

By providing essential information on temperature extremes and energy demands, this research aims to support policymakers in formulating effective mitigation and adaptation plans to address the impacts of climate change in CA.

## 2 Experimental Section

### 2.1 Study Domain

**Figure 1:**
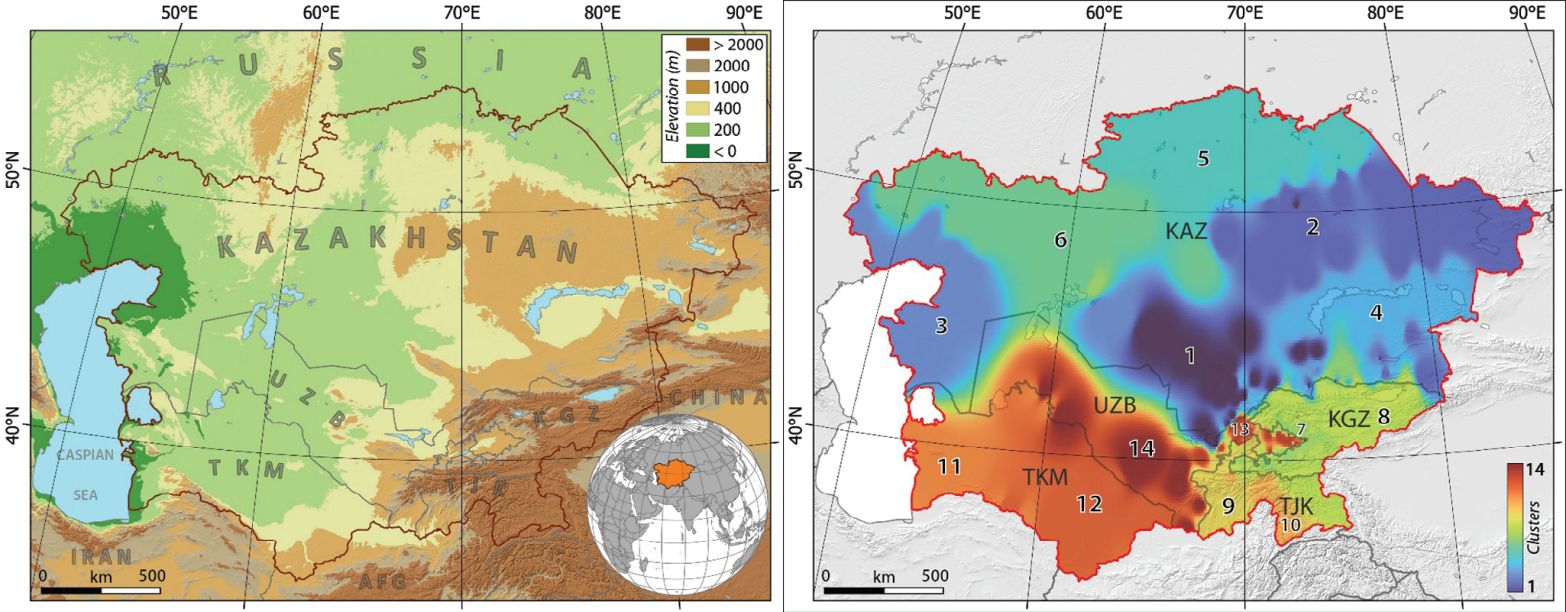
**(Left)** Physiographic map of Central Asia (study area) encompassing Kazakhstan, Kyrgyzstan, Tajikistan, Turkmenistan, and Uzbekistan.; **(Right)** Cluster zoning of CA based on the maximum and minimum temperatures and geographical characteristics.

CA encompasses Uzbekistan, Kazakhstan, Kyrgyzstan, Tajikistan, and Turkmenistan, situated between 35.0° to 55.4°N latitude and 46.5° to 88.0°E longitude (**Figure** **1**). This region features arid and grassland landscapes transitioning from steppes in the north to semi‐deserts in the south. Predominantly, CA experiences arid to semi‐arid climates, shaped mainly by mid‐latitude westerly winds. Substantial temperature fluctuations, limited precipitation, and high evaporation rates were prevalent across much of CA. Notably, Uzbekistan, Turkmenistan, and Kazakhstan, with lower elevations, were particularly affected. Conversely, mountainous areas like Tajikistan and Kyrgyzstan exhibit lower mean temperatures and higher precipitation levels.^[^ ^35^ ^]^ CA has a population of ≈66.3 million. Among the countries, Kazakhstan has the lowest population density while Uzbekistan has the highest.^[^ ^31^ ^]^

### 2.2 Data

In the current research, daily meteorological data were retrieved from the ERA5 (ECMWF Reanalysis v5) reanalysis database, encompassing average daily air temperatures (minimum and maximum) (°C).^[^ ^36^ ^]^ The dataset comprised information from 700 locations across CA, spanning urban, suburban, and rural areas, from 1962 to 2021.

Additionally, GCMs, CMIP6, featuring varied spatial resolutions, were accessed from the C3S center to assess climate change impacts on key variables (Table S1, Supporting Information). Historical CMIP6 model outputs were utilized for the period spanning 1962–2014, while simulation outputs were analyzed for two future periods: the near‐future (2022–2051) and the far‐future (2071–2100).

Here, the Inverse Distance Weighting (IDW) algorithm is employed for interpolation, generating raster outputs with a spatial resolution of 0.1° by 0.1°. The interpolation calculations were based on the four nearest points. To ensure consistency in visualizing all map groups with a unified color bar, the upper and lower limits of the color bar were determined using the 2nd and 98th percentiles, respectively, across all maps. For map groups featuring both negative and positive values, the color bar limit was defined as the maximum absolute value between the 2nd and 98th percentiles, centered at zero.

### 2.3 Clustering using K‐Means Method

To investigate the differences in how various regions within the studied countries respond to climate change, based on their maximum and minimum temperatures and geographical characteristics is decided to group them into clusters. One widely used clustering method was the K‐means algorithm, wherein samples were grouped into groups based on similar characteristics.^[^ ^37^ ^]^ In this approach, the number of clusters was pre‐established, and the validation of the suitable number of clusters was conducted using the Elbow index, which was computed using Equation (1) as proposed by Brusco and Steinley (2007).^[^ ^38^ ^]^

(1) $$WCSS=\sum_{k=1}^{K}\sum_{i\in C_{k}}\sum_{v=1}^{V}{\left(x_{iv}-\bar{x_{vk}}\right)}^{2}$$

The sets of data in the K^th^ cluster are denoted as *C_k_*, and the mean of the variable *v* inside the cluster is represented as $\bar{x_{vk}}$. To ascertain the optimal number of clusters, a graphical representation is constructed wherein the horizontal axis denotes the number of clusters, while the vertical axis is represented by *WCSS*. The computation of the value *K* is performed for different values, commencing from 1 and progressively increasing until the point at which the value *WCSS*stabilizes or reaches a plateau‐like state, typically corresponding to the maximum number of clusters. The point on the plot commonly referred to as the “Elbow” point is typically seen as indicative of the ideal number of clusters for the given data^[^ ^39^ ^]^ (Figure 1b).

### 2.4 Calculation of Climate Indices

The climatic indices outlined by the World Meteorological Organization's (WMO) climatology commission^[^ ^40^ ^]^ were utilized for monitoring severe climatic conditions (Table S2, Supporting Information). The software tool ClimPACT2 (accessible at https://climpact‐sci.org) was employed for computing and evaluating climate indices. The percentage of cool nights is examined (TN10p, %) and days (TX10p, %), warm nights (TN90p, %) and days (TX90p, %), frost days (FD, days), ice days (ID, days), summer days (SU, days), tropical nights (TP, days), cooling degree days (CDDcold18, degree‐days), and heating degree days (HDDheat10, degree‐days) (Table S2, Supporting Information). The long‐term trends of the climatic indicators described above were determined by calculating linear trends using the least squares approach. The statistical significance of these observed trends was assessed using the Mann‐Kendall nonparametric test.^[^ ^32^, ^33^, ^41^, ^42^ ^]^

### 2.5 . Climate Change: Statistical Analysis of GCMs and Different Scenarios

Future climate projections for CA were derived from multiple GCMs^[^ ^43^ ^]^ using two climate scenarios from the CMIP6 archive: SSP2–4.5 and SSP5–8.5. These scenarios address moderate and high global challenges to adaptation and mitigation, respectively, and reflect the global effective radiative forcing expected by 2100.

To correct for modeling discrepancies, bias correction was applied to GCMs outputs using observed data, and spatial resolutions were downscaled for climate change impact assessments. A hybrid semi‐parametric approach was employed for the CMIP6 GCMs data from 2022 to 2100, adjusting statistical properties to align with historical observations.^[^ ^44^ ^]^ This method assumes that disparities between observed and modeled climatic variables remain consistent over time, allowing future projections to be based directly on historical data using a transfer function derived from Equation (2).^[^ ^44^ ^]^

(2) $${\hat{X}}_{m}^{\prime }=\mu_{o}+\frac{\sigma_{o}}{\sigma_{m}}\left({X^{\prime }}_{m}-\mu_{m}\right)$$

${\hat{X}}_{m}^{\prime }$, *X*′_*m*_, µ_*o*_, µ_*m*_, σ_*o*_, and σ_*m*_ correspond to bias‐corrected simulated future mean values, future mean values, historical mean values, simulated historical mean values, historical standard deviation, and simulated historical standard deviation, respectively.

To address divergent results from different GCMs, a “one model, one vote” weighting scheme was used, computing multi‐GCMs ensemble averages by equal weighting and averaging bias‐corrected projections. This approach reduces the uncertainty inherent in individual GCM models.^[^ ^44^ ^]^

### 2.6 Assessment of Bias Correction Performance

The bias and root mean square (RMSE) were computed to statistically assess the performance of the model in simulating the interest variables.^[^ ^45^ ^]^

(3) $$Bias=\frac{1}{N}\sum_{i=1}^{n}\left(b_{i}-o_{i}\right)$$

(4) $$RMSE=\sqrt{\sum_{i=1}^{n}\frac{{\left(b_{i}-o_{i}\right)}^{2}}{n}}$$

### 2.7 Energy Demand for Heating‐Cooling Needs and Associated CO_2_ Emissions

The degree‐day method was employed to evaluate heating‐cooling energy requirements, both at the level of individual buildings and the regional housing stock.^[^ ^46^, ^47^ ^]^ Furthermore, an analysis of CO_2_ emissions per unit of energy consumption was conducted, considering the power system configuration in CA countries (https://www.iea.org/data‐and‐statistics). This evaluation relied on the information provided in **Table** **1**.^[^ ^48^ ^]^

**Table 1:** A carbon footprint of 1 kWh of electric energy is produced using a variety of fossil fuels, renewable energy sources, and green energy.^[^ ^48^ ^]^

Fuel Type	CO_2_ Footprint [gr]
Coal‐fired plant	960
Gas‐fired plant	869
Oil‐fired plant	596
Combined‐cycle gas	450
Hydroelectric	4
PV	100
Wind	15

Moreover, considering the variability in building heat loss rates attributed to increasingly stricter building efficiency standards and changing household preferences for thermal comfort, our methodology entails computing energy requirements per unit of heat loss for heating purposes and thermal transmittance per square meter for cooling purposes, irrespective of building categories and efficiency criteria.

#### 2.7.1 Energy Generation for Heating

(5) Q=P×HDD

where HDD and P are heating degree days and heat loss rate (KWh/°C), respectively.

#### 2.7.2 Energy Generation for Cooling

The total cooling load needed is determined using Equation (6):^[^ ^47^ ^]^

(6) Q=CU×CDD

where CDD, U‐value, and C are cooling degree days, thermal transmittance or heat transfer coefficient (KWh/m^2^×°C), and the area of the building (wm^2^), respectively. Following the ASHRAE standard, the reference temperatures for cooling and heating functions were established at 18 and 10 °C, respectively.^[^ ^49^, ^50^ ^]^ Figure S1 (Supporting Information) shows the flowchart outlining the workflow and key steps of our research process.

## 3 Results and Discussion

### 3.1 Statistical Assessment of Bias Correction Performance

**Table** **2** provides a statistical analysis of multi‐ensemble GCMs for both T_max_ and T_min_ in CA from 1962 to 2021. The corrected multi‐ensemble GCMs models for T_max_ and T_max_ show reduced bias and RMSE compared to the uncorrected models indicating a closer alignment with observed interannual T_max_ and T_min_ variation across CA (Table 2).

**Table 2:** The statistical assessment of multi‐ensembled GCM models of T_max_ and T_min_ in CA between 1962 and 2021.

*Tmax*		
	Bias	RMSE
Raw multi‐ensembled GCM models	2.90	17.68
Bias‐corrected multi‐ensembled GCM models	0.00	16.39

*Tmin*		
	Bias	RMSE
Raw multi‐ensembled GCM models	−6.92	17.62
Bias‐corrected multi‐ensembled GCM models	0.00	16.15

### 3.2 Spatial‐Temporal Changes in the Climate Indices in CA

**Figure 2:**
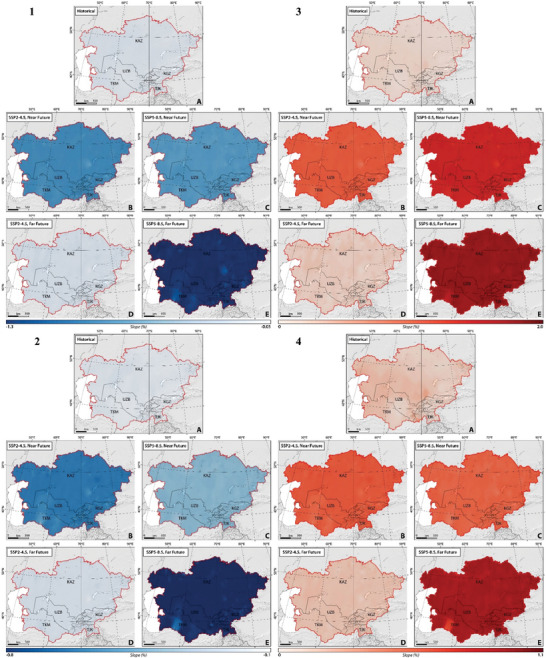
The slope of spatial‐temporal changes of 1) cool days (TX10p), 2) cool nights (TN10p), 3) warm days (TX90p), and 4) warm nights (TN90p) in CA, considering SSP2–4.5 and SSP5–8.5 climate projections between 1962 and 2100.

**Figure 3:**
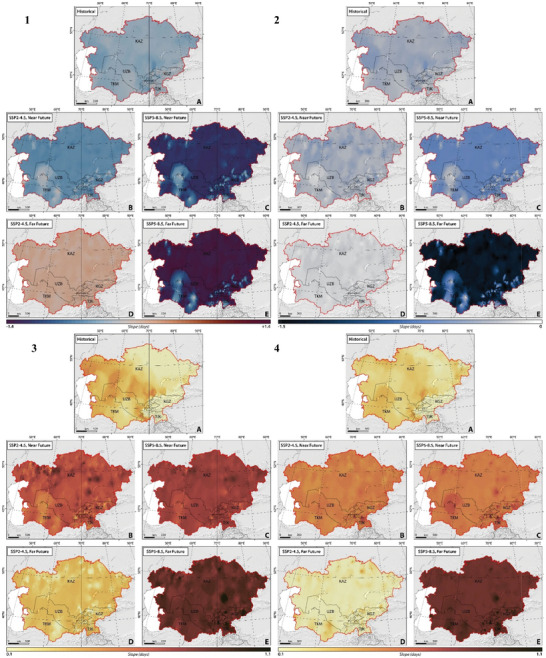
The slope of spatial‐temporal changes of 1) FD, 2) ID, 3) SU, and 4) TR in CA, considering SSP2–4.5 and SSP5–8.5 climate projections between 1962 and 2100.

**Figure 4:**
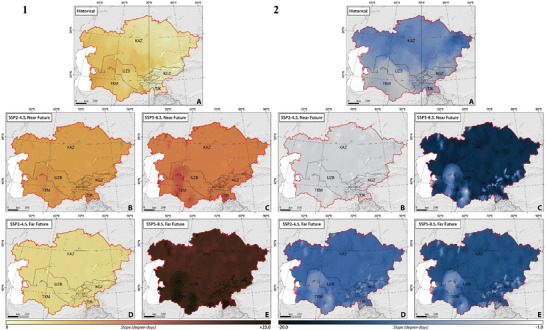
The slope of spatial‐temporal changes of 1) CDDcold18 and 2) HDDheat10 in CA, considering SSP2–4.5 and SSP5–8.5 climate projections between 1962 and 2100.

To analyze projected variations of energy generation and carbon footprint, we examined the statistical significance of both historical and future climate indices of TX10p, TN10p, TX90p, TN90p, FD, ID, SU, TP, CDDcold18, and HDDheat10 (**Figures** 2, 3, 4).

#### 3.2.1 Changing Patterns in the Percentage of Warm‐Cold Days and Nights

Figure 2 illustrates the spatial‐temporal distribution of the trend of changes in TX10p, TN10p, TX90p, and TN90p between 1962 and 2100 under two climate scenarios of SSP2–4.5 and SSP5–8.5. During the historical period, there was a significant rise (*p*‐value < 0.05) in TX90p and TN90p, with the highest mean values recorded in Tajikistan at 0.19 and 0.20, respectively, and the lowest in Kyrgyzstan at 0.13 and 0.14, respectively (Figure 2). TN10p and TX10p experienced a significant reduction, ranging from −0.20 to −0.06 and −0.06 to −0.14, respectively, in Kyrgyzstan (lowest changes), and from −0.20 to −0.08 and −0.20 to −0.08, respectively, in Kazakhstan (highest changes) (Figure 2). The trends classify CA into two halves regarding the magnitude of climate change impact. The western regions show a notable increase in TN90p and TX90p, while the northeastern and southern areas exhibit a less marked trend. Northwestern parts demonstrate a significant decrease in TN90p and TX90p compared to the rest of the region.

Future projections under SSP2–4.5 suggest a significant increase (*p*‐value < 0.05) in TX90p and TN90p, relatively consistent across the studied countries, with slopes ranging from 0.60 ± 0.02 (Kazakhstan) to 0.56 ± 0.05 (Turkmenistan) and from 1.10 ± 0.04 (Tajikistan) to 1.07 ± 0.04 (Turkmenistan), respectively (Figure 2). TX10p and TN10p are expected to decrease significantly (*p*‐value < 0.05), with the highest values being −0.60 ± 0.02 and −0.84 ± 0.03 in Kazakhstan, respectively. In the far‐future, TX90p and TN90p are projected to increase by smaller, non‐significant slopes, and TX10p and TN10p will also show a similar non‐significant decreasing trend. The SSP5–8.5 scenario projects comparable shifts in TX90p, TN90p, TX10p, and TN10p, with higher slopes, reflecting a high‐emission future with significant climate challenges (Figure 2).

Figures S2–S6 (Supporting Information) show the temporal changes in the statistics of averaged TX10p, TX90p, TN10p, and TN90p, separately in studied countries between 1962 and 2100 under both climate scenarios. Figure S7 (Supporting Information) shows the spatial‐temporal distribution of TX10p, TN10pTX90p, and TN90p in CA, between 1962 and 2100 under climate scenarios of SSP2–4.5 and SSP5–8.5. Moreover, Table S3 (Supporting Information) illustrates the statistics of averaged TX10p, TX90p, TN10p, and TN90p, separately in studied countries between 1962 and 2100 under both climate scenarios. To examine the possible variation in the response of different regions of the studied countries to climate change, Tables S4 and S5 (Supporting Information) show the statistics of averaged TX10p, TX90p, TN10p, and TN90p, separately in each cluster between 1962 and 2100 under both climate scenarios.

Notably, there was an increase in TN90p and TX90p from 1962–1991 to 1992–2021 across all countries. The SSP2–4.5 scenario projects a decline in TN90p and TX90p in the near‐future, with slight reductions in the far‐future. TN10p and TX10p decreased between 1992–2021 compared to 1962–1991, with SSP2–4.5 projecting an increase in the near‐ and far‐future. The greatest anticipated rise in the TN10p and TX10p in Tajikistan is projected from 7% to 10% and 7% to 12%, respectively, in the near‐future. Similarly, the most significant decrease in the TN90p and TX90p in Tajikistan is expected to be from 17% to 13% and 17% to 15%, respectively, in the near‐future. Additionally, the shifts from the near‐future to the far‐future are nearly consistent across all countries (Table S3, Supporting Information).

Conversely, the SSP5–8.5 scenario predicts a different trend in TX90p, TN90p, TX10p, and TN10p for both the near‐ and far‐future. Contrary to the SSP2–4.5 scenario, TN90p is anticipated to rise from 1992–2021 to 2022–2051 (near‐future) and is projected to remain constant through the end of the century. Meanwhile, TX90p is projected to decrease between 1992–2021 and 2022–2051 (near‐future) but slightly continue to increase until the end of the century (Table S3, Supporting Information).

By clustering the analyzed countries, to examine the possible variation in the response of different parts of the studied countries to climate change, our multi‐ensemble GCMs analysis suggests that all regions within these countries responded uniformly to climate change under both scenarios (Figures S2–S7 and Tables S4 and S5, Supporting Information).

Global trends reported by the IPCC (2021) show a likely decrease in TX10p and TN10p and an increase in TX90p and TN90p, with robust evidence of extreme temperature increases. However, confidence in these trends varies regionally due to data limitations.^[^ ^51^ ^]^ Similar studies in the Jhelum River Basin and Ethiopia reported significant increases in TX90p and TN90p and decreases in TX10p and TN10p, with more pronounced trends under RCP8.5 compared to RCP4.5.^[^ ^52^, ^53^ ^]^ These findings align with observed patterns in Iran.^[^ ^54^, ^55^ ^]^

#### 3.2.2 Changing Patterns in Frost‐Ice‐Summer Days, and Tropical Nights

Figure 3 illustrates the spatial‐temporal changes of the trends of changes in FD, ID, SU, and TR between 1962 and 2100 under two climate scenarios of SSP2–4.5 and SSP5–8.5. Our findings show a statistically significant decline (*p*‐value < 0.05) in FD and ID and a non‐consistent increase in SU and TR in CA between 1962 and 2100. The highest reductions in FD and ID were in Tajikistan and Kazakhstan with values of −0.33 and −0.32, respectively. Conversely, Turkmenistan experienced the highest increase in SU and TR, with rates of 0.30 ± 0.09 and 0.30 ± 0.05, respectively (Figure 3). The lowest changes in FD, ID, SU, and TR were recorded in Turkmenistan (−0.27 ± 0.07), Turkmenistan (−0.26 ± 0.07), Kyrgyzstan (0.05 ± 0.09), and Kyrgyzstan (0.10 ± 0.09), respectively. The region can be divided into two halves based on these trends: the western areas show a notable increase in SU and TR, while the eastern and southern areas exhibit lesser increases. Northwestern parts demonstrate a significant decrease in FD and ID compared to the rest of the region.

Future projections indicate a significant decline (*p*‐value < 0.05) in both FD and ID in the near‐future under both SSP2–4.5 and SSP5–8.5 scenarios, with ID decreasing more slowly than FD. SU and TR are expected to increase significantly (*p*‐value < 0.05) under both scenarios, with TR increasing more slowly than SU (Figure 3).

Under SSP2–4.5, FD, ID, SU, and TR are projected to change in the near‐future, relatively consistent across the studied countries, with the highest rate of changes being −0.51 ± 0.08, −0.30 ± 0.09, 0.70 ± 0.09, and 0.54 ± 0.07, respectively. However, FD, ID, SU, and TR are projected to change (increase and/or decrease) by smaller slopes (*p*‐value > 0.05) with the highest rates of 0.13 ± 0.05 (Kazakhstan) to ‐0.10 ± 0.04 (Kazakhstan), 0.30 ± 0.08 (Kazakhstan), and 0.22 ± 0.12 (Turkmenistan), respectively during the far‐future. Interestingly, ID is expected to increase under SSP2‐4.5 in the far‐future which is likely due to variations in projected ID occurrences caused by variations in climate change mitigation strategies, model uncertainties, and natural climate fluctuations. The SSP5–8.5 scenario shows comparable trends but with higher slopes due to higher emissions and more significant climate change challenges (Figure 3).

Figures S8–S12 (Supporting Information) show the temporal changes in the statistics of averaged FD, ID, SU, and TR, separately in studied countries between 1962 and 2100 under both climate scenarios. Figure S13 (Supporting Information) shows the spatial‐temporal changes of FD, ID, SU, and TR in CA, between 1962 and 2100 under climate scenarios of SSP2–4.5 and SSP5–8.5. Moreover, Table S6 (Supporting Information) illustrates the statistics of averaged FD, ID, SU, and TR, separately in studied countries between 1962 and 2100 under both climate scenarios. To examine the possible variation in the response of different regions of the studied countries to climate change, Tables S7 and S8 (Supporting Information) show the statistics of averaged FD, ID, SU, and TR, separately in each cluster between 1962 and 2100 under both climate scenarios.

Our multi‐ensemble GCMs analysis revealed regional variations within Kazakhstan (under both SSP2–4.5 and SSP5‐8.5 but with different slopes of changes). The northwest, north, and northeastern regions showed homogeneous responses characterized by slight reductions in FD and ID and sharp increases in SU and TR. Conversely, the southern and southwestern regions showed variable trends, with increases in FD and ID in the near‐future and declines in the far‐future, accompanied by decreases in SU and TR in the near‐future and subsequent rises in the far‐future. Southeastern Kazakhstan displayed a continuous decline in FD and ID and an increase in SU and TR in both near‐ and far‐futures (Figure S2; Figures S8–S12, and Table S7, Supporting Information).

In Kyrgyzstan, the western part exhibited different behavior regarding FD and ID with sharp increases in the near‐future followed by declines. This deviation points to potential microclimatic variations or localized factors influencing the climate in western Kyrgyzstan. The country as a whole showed uniform patterns for SU and TR, suggesting a more cohesive climatic response to rising temperatures. Turkmenistan, Tajikistan, and Uzbekistan displayed uniform behavior across all indices, with declines in SU and TR in the near‐future and increases in the far‐future and increases in FD and ID in the near‐future and declines in the far‐future (Figure S2, Figures S8–S12, and Table S8, Supporting Information).

The existing uniformity underscores the importance of regional climatic factors and geographical influences on climate change responses, essential for devising targeted adaptation and mitigation strategies. Similar studies in the Kashmir Himalaya found declining FD and increasing SU and TR trends in the far‐future under RCP4.5 and RCP8.5 scenarios.^[^ ^56^ ^]^ These studies are consistent with our findings and emphasize the broader implications of climate change globally.

#### 3.2.3 Changing Patterns in Heating‐Cooling Degree Days

Figure 4 illustrates the spatial and temporal distribution of the trend of changes in HDDheat10 and CDDcold18 from 1962 to 2100 under two climate scenarios of SSP2–4.5 and SSP5–8.5. Our findings reveal a statistically significant decline (*p*‐value < 0.05) in HDDheat10, except in Uzbekistan, Tajikistan, and Kyrgyzstan, where the decline is not significant (*p*‐value > 0.05). Conversely, there is a significant increase in CDDcold18 across most regions. The highest reduction in HDDheat10 was detected in Kazakhstan (−8.2 ± 1.70 degree days), whereas Turkmenistan experienced the lowest change (−4.0 ± 0.80 degree days). Turkmenistan saw the highest increase (*p*‐value < 0.05) in CDDcold18 (5.00 ± 1.30 degree days), while the smallest increase was in Kyrgyzstan (0.30 ± 0.05 degree days) (*p*‐value > 0.05) (Figure 4). The region can be classified into two halves: western regions with a notable increase in CDDcold18, and eastern and northwestern areas showing a significant decrease in HDDheat10.

Future projections indicate a continued significant decline (*p*‐value < 0.05) in HDDheat10 and an increase (*p*‐value < 0.05) in CDDcold18 in the near‐future under both SSP2–4.5 and SSP5–8.5 scenarios. Under SSP2–4.5, the highest increase in CDDcold18 is expected in Turkmenistan (8.20 ± 1.2 degree days), while Kazakhstan will see the sharpest decline in HDDheat10 (−11.00 ± 0.97 degree days). In the far‐future, changes are projected to be smaller, with significant increases in CDDcold18 and non‐significant changes in HDDheat10. Similarly, Turkmenistan and Kazakhstan are projected to face the highest increase in CDDcold18 (*p*‐value < 0.05) and a decrease in HDDheat10 (*p*‐value > 0.05) with values of 2.80 ± 0.60 and −2.10 ± 0.24 degree days, respectively. The SSP5–8.5 scenario predicts higher slopes of change compared to SSP2–4.5, reflecting higher emissions and significant climate change challenges (Figure 4).

Figures S14–S18 (Supporting Information) show the temporal changes in the statistics of averaged FD, ID, SU, and TR, separately in studied countries between 1962 and 2100 under both climate scenarios. Figure S19 (Supporting Information) shows the spatial‐temporal changes of FD, ID, SU, and TR in CA, between 1962 and 2100 under climate scenarios of SSP2–4.5 and SSP5–8.5. Moreover, Table S9 (Supporting Information) illustrates the statistics of averaged HDDheat10 and CDDcold18 separately in studied countries between 1962 and 2100 under both climate scenarios. To examine the possible variation in the response of different regions of the studied countries to climate change, Table S10 (Supporting Information) shows the statistics of averaged HDDheat10 and CDDcold18 separately in each cluster between 1962 and 2100 under both climate scenarios.

Our multi‐ensemble GCMs analysis shows that the northwest, north, and northeastern regions of Kazakhstan exhibit similar patterns with sharp reductions in HDDheat10 and increases in CDDcold18. In contrast, the southern and southwestern regions showed distinct but internally consistent behavior with an increase in HDDheat10 in the near‐future and declines in the far‐future, accompanied by a reduction in CDDcold18 in the near‐future and subsequent rises in the far‐future, with southeastern Kazakhstan demonstrating unique patterns throughout the studied period a continuous decline in HDDheat10 and an increase in CDDcold18 in both near‐ and far‐futures. This indicates that regional variations within Kazakhstan significantly influence climate responses, necessitating tailored adaptation strategies for each area (Figures S14–S19 and Table S10, Supporting Information).

In Kyrgyzstan, the western part diverges in HDDheat10 trends, showing increases in the near‐future followed by declines, while other regions exhibit uniform patterns. It highlights a localized discrepancy that could be due to specific geographical or climatic factors. The uniform response in other parts of Kyrgyzstan suggests that localized anomalies aside, broader climatic trends are consistent. Turkmenistan, Tajikistan, and Uzbekistan display consistent behavior with declines in CDDcold18 in the near‐future, and the opposite trends in the far‐future. HDDhat10 is expected to increase in the near‐future and decline in the far‐future, indicating that these countries might experience more consistent climatic conditions or lack the geographical diversity that generates varied responses in Kazakhstan and Kyrgyzstan (Figure S3 and Table S10, Supporting Information).

Projections from 2022 to 2100 under SSP2–4.5 indicate the highest percentage decline in HDDheat10 in Kazakhstan (24%) and the highest increase in CDDcold18 in Kyrgyzstan (132%) in the near‐future. However, the highest percentage of increase is expected in Turkmenistan (254%) followed by Uzbekistan (105%). On the other hand, the highest projected increase in the CDDcold18 is predicted in Kyrgyzstan (132%) and the highest decline in Turkmenistan (57%) in the near‐future. In the far‐future, HDDheat10 is expected to reduce by a percentage of 14% in all countries and CDDcold18 is expected to increase varying between 39% and 40% (**Table** **3**). SSP5–8.5 shows more severe impacts. The increase in cooling demand suggests a strain on socio‐economic development and energy networks, highlighting the need for equitable cooling access. Countries traditionally prepared for heating will need immediate and long‐term adaptations for increased heat resilience and sustainable cooling pathways. Future research should incorporate additional socio‐economic, technical, and environmental factors for precise cooling demand projections, considering varied thermal comfort expectations across regions.

**Table 3:** The percent of changes (%) in the mean heating‐cooling degree days across CA in the near‐ and far‐futures compared to the historical period under both climate scenarios.

SSP2–4.5			SSP5–8.5		
*Kazakhstan*					
*Period*	HDDheat10	CDDcold18	*Period*	HDDheat10	CDDcold18
Near‐future	−24	34	Near‐future	−23	52
Far‐future	−14	39	Far‐future	−40	78

*Kyrgyzstan*					
*Period*	HDDheat10	CDDcold18	*Period*	HDDheat10	CDDcold18
Near‐future	−3	132	Near‐future	−2	165
Far‐future	−14	40	Far‐future	−42	85

*Tajikistan*					
*Period*	HDDheat10	CDDcold18	*Period*	HDDheat10	CDDcold18
Near‐future	32	5	Near‐future	34	20
Far‐future	−14	38	Far‐future	−40	77

*Turkmenistan*					
*Period*	HDDheat10	CDDcold18	*Period*	HDDheat10	CDDcold18
Near‐future	254	−57	Near‐future	258	−51
Far‐future	−14	39	Far‐future	−41	79

*Uzbekistan*					
*Period*	HDDheat10	CDDcold18	*Period*	HDDheat10	CDDcold18
Near‐future	105	−33	Near‐future	108	−24
Far‐future	−14	39	Far‐future	−40	80

The trend for HDDheat10 aligns with FDs and IDs, while CDDcold18 corresponds with TRs and SUs, hinting at complex climatic interactions that merit further investigation. Moreover, our study identifies similar trends in neighboring clusters, reflecting regional climate impact patterns. For instance, southeastern Kazakhstan resembles Kyrgyzstan (excluding the west), and western Kyrgyzstan aligns with northern and northeastern Tajikistan. However, western Kyrgyzstan shows different CDDcold18 patterns compared to eastern Uzbekistan, likely due to topography and altitude differences.

A similar study indicated that regions near the Equator, particularly Sub‐Saharan Africa, will see the most significant increase in CDDs, which carries important implications for their climate resilience planning and building strategies.^[^ ^57^ ^]^ There will be a 30% relative variation in cooling demand in Switzerland and the United Kingdom, which is the highest globally. In the Andes mountain ranges of South America, which extend from north to south, and in the Himalayas of CA, which extend into Southwest China, they also identify substantial increases in CDDs.^[^ ^57^ ^]^

### 3.3 Energy Supply Across CA

**Figure 5:**
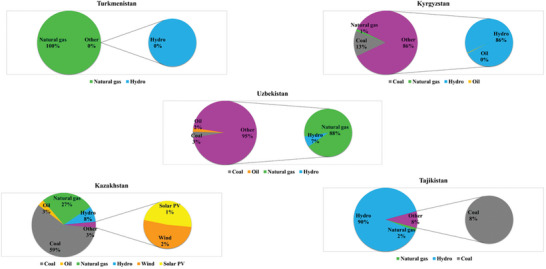
The configuration of the energy supply by source in CA (Data: https://www.iea.org/data‐and‐statistics).

**Figure** **5** shows the distribution of the energy generation system in CA by the end of 2021, representing the end of the historical period. The energy is generated from a diverse mix of traditional fossil fuel, low‐carbon, and renewable energy sources. Coal, abundant in the region, plays a crucial role in electricity production, providing a reliable energy source, mainly in Kazakhstan with 59% (Figure 5). Natural gas, particularly significant in countries like Turkmenistan, Uzbekistan, and Kazakhstan, is essential for various applications, including power generation and heating. Specifically, natural gas is the main and only source of energy generation in Turkmenistan. Kazakhstan and Uzbekistan stand out for their oil production, contributing significantly to the energy sector, with 3 and 2%, respectively. Hydropower, utilized extensively in Kyrgyzstan and Tajikistan, with 86 and 90%, respectively, due to their abundant water resources, serves as a clean and renewable energy source, reducing reliance on fossil fuels. Furthermore, Kazakhstan is increasingly focusing on incorporating renewable energy sources such as solar and wind power into its energy mixed system, showcasing a commitment to sustainable energy practices. At the end of 2021, a total of 3% of energy sources belonged to solar and wind power systems, with the highest percentage of wind farms (2%).

Figures S20 and S21 (Supporting Information) present the temporal changes in energy transition indicators in CA between 1990 and 2021, adopted from (https://www.iea.org/data‐and‐statistics). **Table** **4** shows the observed trend of changes in energy transition indicators over time, using the Man‐Kendall trend test. The transition indicators demonstrate a statistically significant (*p*‐value < 0.05) shift toward cleaner and more sustainable energy sources, particularly in Kyrgyzstan (slope of 40%) and Tajikistan, while Uzbekistan shows a statistically significant decline in using renewable energy sources (slope of −6%). Although Kazakhstan is moving toward renewable energy sources, these changes are not statistically significant (*p*‐value > 0.05). Our analysis reveals that while traditional fossil fuels such as coal, natural gas, and oil historically dominated the region's energy mixed structure, there is a noticeable rise in the share of renewable sources like hydropower. Despite this transition, there is a significant increase in the use of natural gas (observed in Kazakhstan and Uzbekistan) and coal (with an increase of 185%) in Kyrgyzstan. In Kyrgyzstan and Tajikistan, the share of natural gas in power generation is decreasing (*p*‐value < 0.05), with a steeper decline in Kyrgyzstan (slope of −98%). The share of oil in energy generation is decreasing (*p*‐value < 0.05) in Kazakhstan and Uzbekistan with Uzbekistan exhibiting a sharper decline (slope of −39%).

**Table 4:** The observed trend of changes (%) in energy transition indicators using the Man‐Kendall trend test in CA between 1990 and 2021.

Energy Transition Indicator	Share of renewables in power generation		Share of low carbon sources in power generation		Share of coal in power generation		Share of oil in power generation		Share of gas in power generation	
	Slope	*p*‐value	Slope	*p*‐value	Slope	*p*‐value	Slope	*p*‐value	Slope	*p*‐value
***Kazakhstan***	no trend	> 0.05	no trend	> 0.05	no trend	> 0.05	−24	<0.05	30	<0.05
***Kyrgyzstan***	40	<0.05	40	<0.05	43	<0.05	no trend	> 0.05	−98	<0.05
***Tajikistan***	20	<0.05	20	<0.05	185	<0.05	no share	no share	−47	<0.05
***Turkmenistan***	no share	no share	no share	no share	no share	no share	no share	no share	whole share	whole share
***Uzbekistan***	−6	<0.05	−6	<0.05	−1.3	<0.05	−39	<0.05	34	<0.05

Our analysis suggests that CA holds a diverse energy profile across its nations, with considerable reliance on fossil fuels in some areas and a significant dependency on hydropower in others (Figure 5). The region faces considerable challenges due to climate change, such as increased cooling demands and variable energy resources. Despite a growing emphasis on sustainability and cleaner energy options, these countries still heavily rely on traditional fossil fuels like gas and coal. Factors such as existing infrastructure, economic considerations, and energy security concerns contribute to this continued reliance. This complexity underscores the challenge of balancing sustainable energy practices with meeting immediate energy needs in this region.^[^ ^58^, ^59^, ^60^, ^61^, ^62^, ^63^, ^64^, ^65^ ^]^

While each Central Asian country faces unique challenges due to its current energy generation profile, it is feasible for the region to combat and mitigate climate change through strategic investments, policy support, and regional cooperation. Emphasizing renewable energy expansion, enhancing energy efficiency, and modernizing infrastructure is key to ensuring a sustainable and reliable energy future for CA. By leveraging their renewable energy potential and addressing specific local challenges, these countries can collectively contribute to global climate change mitigation efforts.^[^ ^58^ ^]^

### 3.4 Energy Generation for Heating‐Cooling Purposes in CA

Given the differences in countries' energy generation structures during the historical period, we used data from 2021—the end of our historical period and the latest available energy supply configuration—as the baseline for calculating energy generation in both the near‐ and far‐future. Moreover, due to the variability in building heat loss rates attributed to increasingly stricter building efficiency standards and changing household preferences for thermal comfort, our methodology entails computing energy requirements per unit of heat loss for heating purposes and thermal transmittance per square meter for cooling purposes, irrespective of building categories and efficiency criteria.

**Figure 6:**
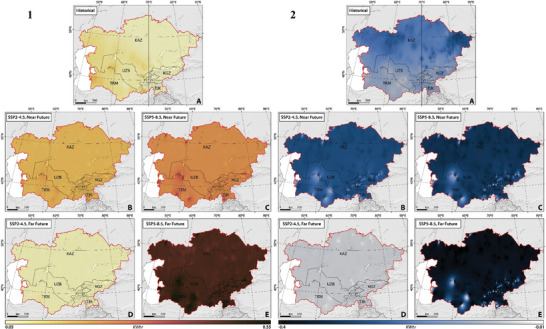
The trend of changes in 1) cooling energy generation (kWh) and 2) heating energy generation (kWh) in CA, considering SSP2–4.5 and SSP5–8.5 climate projections between 1962 and 2100.

Aligned with Equations 5 and 6, our calculations reflect the distribution of CDDcold18 and HDDheat10, forecasting that the spatial and temporal variations in energy generation for heating‐cooling will align with the projected spatial‐temporal distribution of CDDcold18 and HDDheat10, respectively, in both the near‐ and far‐future under the studied climate scenarios. **Figure** **6** illustrates the spatial and temporal distribution of the trend of changes in heating‐cooling energy generation from 1962 to 2100 under two climate scenarios of SSP2–4.5 and SSP5–8.5. The results indicate a statistically significant decline (*p*‐value < 0.05) in heating energy generation, despite a significant increase in cooling energy generation, except in Uzbekistan, Tajikistan, and Kyrgyzstan, where declines were not statistically significant (*p*‐value > 0.05), within CA between 1962 and 2021 (Figure 6).

Future projections (SSP2–4.5) indicate a significant decline (*p*‐value < 0.05) in heating energy generation and a significant increase (*p*‐value < 0.05) in cooling energy generation during the near‐future under both examined scenarios (Figure 6). However, both heating‐cooling energy generations are projected to change (increase and/or decrease) by smaller slopes, significant changes in cooling energy generation, and non‐significant changes in heating energy generation in the far‐future. As expected, under the SSP2‐4.5 scenario, the situation is projected to be better in terms of mitigating the most severe impacts of climate change in the far‐future. The fossil‐fueled development scenario (SSP5–8.5) projected comparable shifts in heating‐cooling energy generations with higher slopes compared to the middle‐of‐the‐road scenario (SSP2–4.5) (Figure 6), which is expected since SSP5‐8.5 depicts a future with high emissions and significant climate change challenges.

Table S11 (Supporting Information) presents the energy generation for heating‐cooling purposes between 2022 and 2100 across CA under both climate scenarios. According to our analysis, in all studied countries there will be a decline in heating energy generation in the far‐future compared to the near‐future despite the estimated increase in cooling energy generation, which aligns with our findings about energy demand indices (HDDheat10 and CDDcold18) (Table S11, Supporting Information). Transitioning from SSP2‐4.5 to the warmer SSP5‐8.5 scenario would significantly increase heat exposure and energy demand for cooling across CA. However, despite these projected increases in cooling demands, the total energy generation is expected to remain nearly constant in both the near‐ and far‐future. This stability reflects the shift from meeting heating demands to fulfilling cooling demands as a result of global warming.^[^ ^57^, ^66^, ^67^ ^]^

Our findings indicate that increases in cooling energy generation are associated with reductions in heating energy generation needs. Projections show that from 2022 to 2100, under the SSP2‐4.5 scenario, heating energy generation is expected to decrease by 15%, while cooling energy generation is predicted to rise by ≈38% (**Table** **5**). In contrast, the SSP5–8.5 scenario forecasts a more severe impact with a 38% reduction in heating energy generation and an 87% increase in cooling energy generation (Table 5). Our findings raise critical questions about prioritizing sustainable cooling access and developing heat resilience strategies in these countries.

**Table 5:** The percent of changes (%) in the mean heating‐cooling energy generation separately in the studied countries in the near‐ and far‐futures under both climate scenarios.

*SSP2–4.5*		
Kazakhstan		
	Heating energy generation	Cooling energy generation
The percentage of changes (%)	−15	38
Kyrgyzstan		
	Heating energy generation	Cooling energy generation
The percentage of changes (%)	−15	39
Tajikistan		
	Heating energy generation	Cooling energy generation
The percentage of changes (%)	−15	36
Turkmenistan		
	Heating energy generation	Cooling energy generation
The percentage of changes (%)	−15	38
Uzbekistan		
	Heating energy generation	Cooling energy generation
The percentage of changes (%)	−15	38

*SSP5–8.5*		
Kazakhstan		
	Heating energy generation	Cooling energy generation
The percentage of changes (%)	−38	86
Kyrgyzstan		
	Heating energy generation	Cooling energy generation
The percentage of changes (%)	−40	92
Tajikistan		
	Heating energy generation	Cooling energy generation
The percentage of changes (%)	−38	82
Turkmenistan		
	Heating energy generation	Cooling energy generation
The percentage of changes (%)	−38	87
Uzbekistan		
	Heating energy generation	Cooling energy generation
The percentage of changes (%)	−38	87

These trends raise crucial concerns about the need for sustainable cooling access and robust heat resilience strategies. The significant increase in cooling energy generation alongside the decline in heating demands underscores the necessity for adaptive energy policies and investments in modern infrastructure to handle these evolving energy requirements. Furthermore, these changes highlight the broader implications of climate change on energy systems and emphasize the importance of sustainable development paths to mitigate adverse effects and ensure stable, resilient energy supplies.^[^ ^57^, ^66^, ^67^ ^]^

To ensure resilient cities to heat and stable power generation systems in CA, countries must diversify and expand renewable energy sources, modernize energy infrastructure, implement energy efficiency measures, adopt adaptive policies, and foster regional cooperation and knowledge sharing. Sustainable cooling solutions and regular monitoring and evaluation of energy demands are also essential. Moreover, enhanced construction techniques, the utilization of new materials, improved thermal quality standards for both new and existing buildings and considerations for energy end‐use aspects, such as regular inspections of boilers and central air conditioning systems, are indeed crucial. These strategic actions will help manage increased cooling demands, reduce greenhouse gas emissions, and enhance climate resilience, contributing to successful climate change mitigation efforts in the region.^[^ ^2^, ^68^, ^69^, ^70^, ^71^, ^72^, ^73^, ^74^ ^]^

### 3.5 Carbon Footprint Caused by Power Generation for Heating‐Cooling Purposes in CA

**Figure 7:**
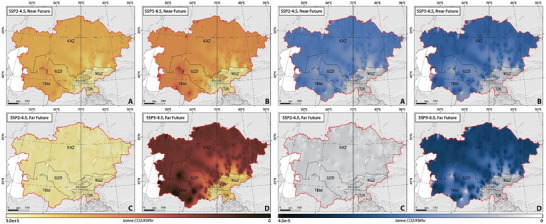
The trend of changes in (**Left**) CO_2_ emission for cooling needs (tonne CO_2_/kWh) and (**Right**) CO_2_ emission for heating needs (tonne CO_2_/kWh) in CA, considering SSP2–4.5 and SSP5–8.5 climate projections between 1962 and 2100.

**Figure** **7** shows the spatial and temporal CO_2_ emissions per unit of energy generated for heating‐cooling from 1962 to 2100 under two climate scenarios of SSP2–4.5 and SSP5–8.5. Given the varying energy generation structures over historical periods, we used data from 2021—the latest and most comprehensive energy supply configuration—as a baseline for future calculations. This approach helps avoid inaccuracies due to historical changes in the energy systems of the studied countries.

Our carbon footprint calculations for CA, spanning 2022 to 2100, show how energy generation for cooling and heating varies spatially and temporally, with notable differences in Kyrgyzstan and Tajikistan owing to their predominant use of hydropower. Future projections under SSP2‐4.5 indicate a significant reduction (*p*‐value < 0.05) in CO_2_ emissions from heating and a significant increase (*p*‐value < 0.05) for cooling in the future across both scenarios (Figure 7). Nonetheless, CO_2_ emissions are projected to shift modestly, with pronounced changes in cooling and minor changes in heating in the far‐future. Kyrgyzstan and Tajikistan's low‐carbon energy sources keep their CO_2_ emission increases lower compared to other Central Asian countries (Figure 7).

Expectations under SSP2‐4.5 suggest improved outcomes for minimizing severe climate impacts in the far‐future. Conversely, the SSP5‐8.5 scenario predicts steeper increases in CO_2_ emissions for both heating and cooling, illustrating a high‐emission, high‐climate‐risk future.

Table S12 (Supporting Information) presents the CO_2_ emissions for heating‐cooling purposes between 2022 and 2100 across CA under both climate scenarios. According to our analysis, in all studied countries there will be a decline in CO_2_ emissions for heating needs in the far‐future compared to the near‐future despite the estimated increase in CO_2_ emissions for cooling needs, which aligns with our findings about energy demand indices (HDDheat10 and CDDcold18) and consequently heating‐cooling energy generation (Table S12, Supporting Information). Transitioning from SSP2‐4.5 to the warmer SSP5‐8.5 scenario would significantly increase CO_2_ emissions for cooling purposes across CA. However, despite these projected increases in CO_2_ emissions for cooling demands, the total CO_2_ emissions are expected to remain nearly constant in both the near‐ and far‐future. This stability reflects the shift from meeting heating demands to fulfilling cooling demands as a result of global warming.^[^ ^57^, ^66^, ^67^ ^]^ According to our results, it is worth mentioning that CO_2_ emissions for heating‐cooling needs have a small order of magnitude in Tajikistan and Kyrgyzstan compared to the results of Central Asian countries under both climate scenarios, despite the nearly similar heating‐cooling energy generations across CA (Table S11, Supporting Information). The emitted CO_2_ is estimated to be ≈10 times higher in the rest of the countries in the future, with the highest projected values in Turkmenistan, under the SSP2‐4.5 scenario, with the value of 1.56 × 10^−2^ and 4.06 × 10^−2^ tonne kWh^−1^ for cooling and heating purposes, respectively in the near‐future. In the far‐future, CO_2_ emissions will increase to 2.18 × 10^−2^ tonne kWh^−1^ and decrease to 3.49 × 10^−2^ tonne kWh^−1^ for cooling and heating needs, respectively (Table S12, Supporting Information). The lowest values are projected in Tajikistan in the future under both climate scenarios.

Our findings reveal that increases in CO_2_ emissions for cooling are typically counterbalanced by reductions in emissions for heating. From 2022 to 2100, under SSP2‐4.5, heating‐related CO_2_ emissions are projected to decrease by 14%, while cooling‐related emissions are expected to increase by ≈41% (**Table** **6**). Under SSP5‐8.5, these numbers become more severe, with a 39% reduction in heating emissions and an 80% increase in cooling emissions across CA (Table 6).

**Table 6:** The percent of changes (%) in the mean CO_2_ emission for heating‐cooling purposes separately in the studied countries in the near‐ and far‐futures under both climate scenarios.

SSP2–4.5		
Period	Kazakhstan	
	CO_2_ emission for heating (tonne CO_2_/kWh)	CO_2_ emission for cooling (tonne CO_2_/kWh)
The percentage of changes (%)	−14	39
Period	Kyrgyzstan	
	CO_2_ emission for heating (tonne CO_2_/kWh)	CO_2_ emission for cooling (tonne CO_2_/kWh)
The percentage of changes (%)	−14	40
Period	Tajikistan	
	CO_2_ emission for heating (tonne CO_2_/kWh)	CO_2_ emission for cooling (tonne CO_2_/kWh)
The percentage of changes (%)	−14	38
Period	Turkmenistan	
	CO_2_ emission for heating (tonne CO_2_/kWh)	CO_2_ emission for cooling (tonne CO_2_/kWh)
The percentage of changes (%)	−14	39
Period	Uzbekistan	
	CO_2_ emission for heating (tonne CO_2_/kWh)	CO_2_ emission for cooling (tonne CO_2_/kWh)
The percentage of changes (%)	−14	39

SSP5–8.5		
Period	Kazakhstan	
	CO_2_ emission for heating (tonne CO_2_/kWh)	CO_2_ emission for cooling (tonne CO_2_/kWh)
The percentage of changes (%)	−40	78
Period	Kyrgyzstan	
	CO_2_ emission for heating (tonne CO_2_/kWh)	CO_2_ emission for cooling (tonne CO_2_/kWh)
The percentage of changes (%)	−42	85
Period	Tajikistan	
	CO_2_ emission for heating (tonne CO_2_/kWh)	CO_2_ emission for cooling (tonne CO_2_/kWh)
The percentage of changes (%)	−40	77
Period	Turkmenistan	
	CO_2_ emission for heating (tonne CO_2_/kWh)	CO_2_ emission for cooling (tonne CO_2_/kWh)
The percentage of changes (%)	−41	79
Period	Uzbekistan	
	CO_2_ emission for heating (tonne CO_2_/kWh)	CO_2_ emission for cooling (tonne CO_2_/kWh)
The percentage of changes (%)	−40	80

Fossil fuels as the primary source of national energy pose significant threats to the environment due to the emission of CO_2_ gas during combustion. This contributes to the accumulation of greenhouse gases and an increase in the carbon footprint. Energy consumption is directly linked to climate change, leading to higher greenhouse gas emissions and its associated consequences.^[^ ^75^, ^76^, ^77^, ^78^ ^]^ To meet the target of restricting global warming to below 1.5 °C, as outlined in the Paris Agreement, it is imperative to reduce annual GHG emissions by half within the next few years. Additionally, implementing net‐zero emissions pledges could help in reaching the Paris Agreement's target of remaining well below 2 °C of warming.^[^ ^75^, ^76^, ^77^, ^78^ ^]^

To achieve this aim, if all Central Asian countries adopted renewable energy sources for heating and cooling, following the examples of Tajikistan and Kyrgyzstan, the region would see a significant reduction in CO_2_ emissions and a considerably lower carbon footprint in the future. Tajikistan and Kyrgyzstan's extensive use of hydropower has enabled them to maintain low emissions levels, demonstrating that a transition to renewable sources like solar, wind, and optimized hydropower can minimize carbon emissions across the region. This shift not only reduces air pollution and associated health risks but also ensures greater energy security and economic benefits through job creation and long‐term cost savings.^[^ ^63^, ^64^ ^]^ Nevertheless, it is important to emphasize that the construction of hydroelectric dams has substantial environmental consequences. The construction of these dams can have adverse impacts on animals and ecosystems by modifying the migration patterns of species such as salmon and decreasing the levels of dissolved oxygen in water. In addition, they have the ability to influence the regional climate through alterations in water cycles and the release of GHG, such as methane and CO_2_, from their reservoirs.^[^ ^79^, ^80^, ^81^ ^]^ The environmental alterations can cause the destruction of habitats and displacement of species, leading to a significant reduction of up to 80% in fish populations within specific river systems. Despite the benefits of hydroelectric power plants, such as their ability to decrease GHG emissions and reduce reliance on fossil fuels, their potential negative impacts on local ecosystems and communities have resulted in increased attention and the requirement for environmental assessments prior to construction. Despite these challenges, hydroelectric power is crucial for fulfilling worldwide electricity demands and promoting sustainable energy development.^[^ ^79^, ^80^, ^81^ ^]^

However, the transition presents challenges that need to be addressed, such as substantial investments in renewable energy infrastructure, the development of strong policy frameworks, and the need for technology and skills development. By overcoming these challenges, Central Asian countries can protect their ecosystems, mitigate climate change impacts, and achieve a sustainable and resilient energy future. The region's commitment to renewable energy, inspired by Tajikistan and Kyrgyzstan's success, will ensure long‐term environmental and economic health, contributing significantly to global climate change mitigation efforts.

## 4 Summary and Conclusion

We explored the adverse impacts of climate change on heating‐cooling energy generation and the resultant CO_2_ emissions, using an extensive dataset (ECMWF Reanalysis v5) covering extreme air temperatures from 1962 to 2021. To assess climate change impacts, we selected and downloaded GCMs from CMIP6 with various spatial resolutions from Copernicus, covering two future periods: near‐future (2022–2051) and far‐future (2071‐2100) under both moderate (SSP2–4.5) and high‐emission (SSP5–8.5) scenarios across CA. Through spatial and temporal evaluations of climatic indices, we systematically analyzed the associated trends.

The bias correction method enhanced the accuracy of multi‐ensemble GCMs in estimating mean maximum and minimum temperatures, resulting in more precise predictions. Under SSP2‐4.5, future projections indicate a significant rise (*p*‐value < 0.05) in TX90p and TN90p across the studied countries, with slopes ranging from 0.60 ± 0.02 (Kazakhstan) to 0.56 ± 0.05 (Turkmenistan) and from 1.10 ± 0.04 (Tajikistan) to 1.07 ± 0.04 (Turkmenistan), respectively. TX10p and TN10p are expected to decrease significantly (*p*‐value < 0.05), notably in Kazakhstan. In the far‐future, these trends are projected to continue but with smaller, non‐significant slopes. The SSP5‐8.5 scenario predicts similar but more pronounced changes, reflecting a higher‐emission future. Additionally, significant declines (*p*‐value < 0.05) in FDs and IDs are expected under both scenarios, with IDs decreasing more slowly than FDs, while Sus and TRs increase substantially, with TRs increasing more gradually.

In the near‐future, under both scenarios, HDDheat10 is projected to decline significantly (*p*‐value < 0.05), and CDDcold18 is expected to rise significantly (*p*‐value < 0.05), with Turkmenistan and Kazakhstan showing the most notable changes. The far‐future shows continued significant increases in CDDcold18 and smaller, non‐significant changes in HDDheat10.

Regional assessments using multi‐ensemble GCMs suggest uniform responses to climate change across CA in terms of TX90p, TN90p, TX10p, and TN10p. However, regional variations include homogeneous responses in the northwest, north, and northeastern regions, characterized by reductions in FD and ID and increases in SU and TR. The southern and southwestern regions displayed variable trends in FD, ID, SU, and TR, with southeastern Kazakhstan showing consistent declines in FD and ID and increases in SU and TR. Additionally, in Kyrgyzstan, the western part exhibited different behavior regarding FD and ID, with sharp increases in the near‐future followed by declines. The trend for HDDheat10 aligns with FDs and IDs, while CDDcold18 corresponds with TRs and SUs, hinting at complex climatic interactions that merit further investigation.

For energy generation, SSP2‐4.5 projects a significant drop (*p*‐value < 0.05) in heating energy generation and a substantial rise (*p*‐value < 0.05) in cooling energy generation in the near‐future. From 2022 to 2100, SSP2‐4.5 forecasts a 15% decrease in heating energy generation and a 38% increase in cooling energy generation, while SSP5‐8.5 predicts a 38% reduction in heating and an 87% rise in cooling energy generation. CO_2_ emissions for cooling are expected to rise while heating emissions decline, with the emissions being significantly lower in Tajikistan and Kyrgyzstan due to their reliance on low‐carbon energy sources. Under SSP2‐4.5, heating‐related CO_2_ emissions are projected to decrease by 14%, and cooling‐related emissions to increase by 41%. Under SSP5‐8.5, the decline in heating emissions is expected to be 39%, with an 80% increase in cooling emissions. The SSP5‐8.5 scenario indicates steeper changes in energy generation and carbon footprint values, illustrating a high‐emission, high‐climate‐risk future.

The following recommendations are suggested in the light of findings for Central Asian countries to mitigate climate change impacts on heating‐cooling energy generation and reduce CO_2_ emissions:

1. Expanding renewable energy through investing in solar and wind energy infrastructure and optimizing hydropower resources in countries like Tajikistan and Kyrgyzstan.
2. Diversifying energy mix by ensuring a balanced mix of renewable energy sources and exploring emerging technologies like geothermal and bioenergy.

## Conflict of Interest

The authors declare no conflict of interest.

## Author Contributions

P.B. and M.B. contributed equally to this work. P.B. wrote, reviewed, and edited the original draft, conceptualization, methodology, validation, formal analysis, investigation, data curation, and data analysis. M.B. wrote, reviewed, and edited the original draft, conceptualization, methodology, validation, resources, project administration, and funding acquisition. A.M.F. and A.R. performed formal analysis, data curation, and data analysis. S.S. performed formal analysis and data Curation. M.L. performed an investigation and reviewed and edited the original draft. J.R.K. performed resources, data curation, project administration, and funding acquisition, and reviewed and edited the original draft.

## Supporting information



Supporting Information

## Data Availability

The data that support the findings of this study are available from the corresponding author upon reasonable request.
