# Supplementary material for: Energy Generation and Carbon Footprint under Future Projections (2022–2100) of Central Asian Temperature Extremes
Source: Glob Chall. 2025 Mar 31;9(5):2400356. doi: 10.1002/gch2.202400356 (PMC12065095; doi:10.1002/gch2.202400356)
Supplement: Supplementary file 1 — Supporting Information [file GCH2-9-2400356-s001.docx]

**Energy generation and carbon footprint under Future Projections (2022-2100) of Central Asian Temperature Extremes**

Parya Broomandi^1,2#^, Mehdi Bagheri^2#*^, Ali Mozhdehi Fard^3^, Aram Fathian^4,5,6^, Mohammad Abdoli^7^, Adib Roshani^3^, Sadjad Shafiei^2^, Michael Leuchner^7^, Jong Ryeol Kim^1*^

^1^ Department of Civil and Environmental Engineering, School of Engineering and Digital Sciences, Nazarbayev University, Kabanbay Batyr Ave. 53, Astana 010000 Kazakhstan.

^2^Department of Electrical and Computer Engineering, School of Engineering and Digital Sciences, Nazarbayev University, Kabanbay Batyr Ave. 53, Astana 010000 Kazakhstan.

^3^Faculty of Civil Engineering, Babol Noshirvani University of Technology, 484, Babol, Iran.

^4^Neotectonics and Natural Hazards Institute, RWTH Aachen University, Aachen 52056, Germany.

^5^UNESCO Chair on Coastal Geo-Hazard Analysis, Research Institute for Earth Sciences, Tehran 13185-1494, Iran.

^6^Water, Sediment, Hazards, and Earth-surface Dynamics (waterSHED) Lab, Department of Geoscience, University of Calgary, Calgary Alberta T2N 1N4, Canada.

^7^Physical Geography and Climatology, Department of Geography, RWTH Aachen University, Wüllnerstr. 5b, 52062 Aachen, Germany.

**#**As the first authors with the same authorship contribution.

* Corresponding authors. Email: jong.kim@nu.edu.kz, and Phone: +7 (7172) 70-91-36.

Email: mehdi.bagheri@nu.edu.kz, and Phone: +7 (7172) 70-92-51.

| **Table S1.** The list of GCM models used in the current study ([https://www.ipcc.ch/report/ar6](https://www.ipcc.ch/report/ar6/wg1/downloads/report/IPCC_AR6_WGI_AnnexII.pdf)). | | |
| --- | --- | --- |
| MODELS | Resolution(km) and Number of Levels (L) | Institution/Country |
| ACCESS-CM2 | 140 km, 85 L | csiro-access csiro and Australia. res. council center of excellence for climate system science, Australia |
| BCC-CSM2-MR | 100 km, 46 L | BCC, Beijing Climate Centre, China |
| CESM2 | 100 km | NCAR, National Center for Atmospheric Research, USA |
| FGOALS-f3-L | 90 km, 32 L | CAS, Chinese Academy of Sciences, China |
| GFDL-ESM4 | 100 km, 49 L | NOAA-GFDL, National Oceanic and Atmospheric Administration, Geophysical Fluid Dynamics Laboratory, USA |
| IPSL-CM6A-LR | 160 km, 79 L | IPSL, Institute Pierre- Simon Laplace, France |
| MIROC6 | 250 km, 40 L | MIROC Consortium JAMSTEC, AORI, NIES, R-CCS, Japan |
| MIROC-ES2L | 250 km, 40 L | MIROC Consortium JAMSTEC, AORI, NIES, R-CCS, Japan |
| MPI-ESM1-2-LR | 170 km, 47 L | MPI-M, Max Planck Institute for Meteorology, Germany |
| MRI-ESM2-0 | 100 km, 80 L | MRI, Meteorological Research Institute, Japan |

| **Table S2.** Climate indexes with their definitions and units used in the current study. | | | | |
| --- | --- | --- | --- | --- |
| **ID** | **Index Name** | **Definition** | **Unit** | **Sectors of Economics** |
| TX10p | Amount of cool days. | Percentage of days when TX < 10th percentile. | % | Energy |
| TN90p | Amount of warm nights. | Percentage of days when TN > 90th percentile. | % | Energy |
| TN10p | Amount of cool nights. | Percentage of days when TN < 10th percentile. | % | Energy |
| TX90p | Amount of warm days. | Percentage of days when TX > 90th percentile. | % | Energy |
| FD | Frost Days | Number of days when TN < 0°C | days | Health and Agriculture and Food Security |
| ID | Ice Days | Number of days when TX < 0°C | days | Health and Agriculture and Food Security |
| SU | Summer Days | Number of days when TX > 25°C | days | Health |
| TR | Tropical nights | Number of days when TN > 25°C | days | Health and Agriculture and Food Security |
| CDDcold18 | Cooling Degree Days. | Annual sum of TM - *n* (where *n* is a user-defined location-specific base temperature and TM > *n*). | degree-days | Health |
| HDDheat10 | Heating Degree Days. | Annual sum of TM - *n* (where *n* is a user-defined location-specific base temperature and TM <*n*) | degree-days | Health |


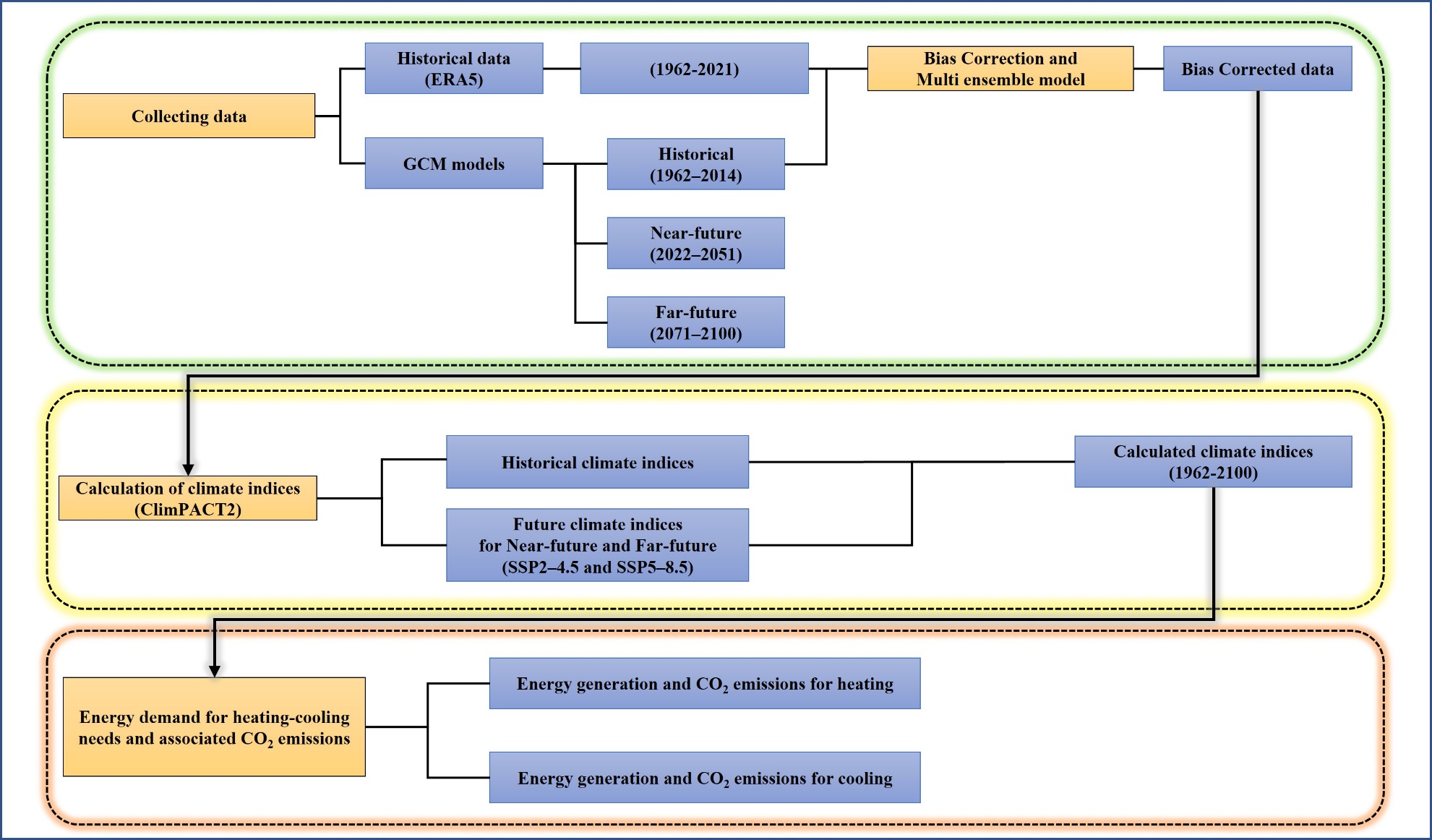
 **Fig. S1.**The workflow and key steps of the current research study.

|  | **Table S3.** The statistics of calculated climate indices (averaged TX10p, TX90p, TN10p, and TN90p) separately in studied countries between 1962 and 2100 under both climate scenarios. | | | | | | | | | | |
| --- | --- | --- | --- | --- | --- | --- | --- | --- | --- | --- | --- |
| ***SSP2–4.5*** | Time Period | ***Kazakhstan*** | | | | ***SSP5–8.5*** | Time Period | ***Kazakhstan*** | | | |
|  |  | **TX10p** | **TX90p** | **TN10p** | **TN90p** |  |  | **TX10p** | **TX90p** | **TN10p** | **TN90p** |
|  | Historical 1 | 11 | 10 | 11 | 10 |  | Historical 1 | 11 | 10 | 11 | 10 |
|  | Historical 2 | 8 | 16 | 8 | 16 |  | Historical 2 | 8 | 16 | 8 | 16 |
|  | Near-future | 10 | 13 | 12 | 15 |  | Near-future | 10 | 14 | 11 | 18 |
|  | Far-future | 11 | 11 | 10 | 14 |  | Far-future | 13 | 14 | 14 | 18 |
|  | Time Period | ***Kyrgyzstan*** | | | |  | Time Period | ***Kyrgyzstan*** | | | |
|  |  | **TX10p** | **TX90p** | **TN10p** | **TN90p** |  |  | **TX10p** | **TX90p** | **TN10p** | **TN90p** |
|  | Historical 1 | 10 | 11 | 11 | 11 |  | Historical 1 | 10 | 11 | 11 | 11 |
|  | Historical 2 | 8 | 15 | 8 | 15 |  | Historical 2 | 8 | 15 | 8 | 15 |
|  | Near-future | 10 | 13 | 12 | 15 |  | Near-future | 10 | 14 | 11 | 18 |
|  | Far-future | 11 | 11 | 10 | 13 |  | Far-future | 13 | 14 | 14 | 18 |
|  | Time Period | ***Tajikistan*** | | | |  | Time Period | ***Tajikistan*** | | | |
|  |  | **TX10p** | **TX90p** | **TN10p** | **TN90p** |  |  | **TX10p** | **TX90p** | **TN10p** | **TN90p** |
|  | Historical 1 | 11 | 10 | 11 | 10 |  | Historical 1 | 11 | 10 | 11 | 10 |
|  | Historical 2 | 7 | 17 | 7 | 17 |  | Historical 2 | 7 | 17 | 7 | 17 |
|  | Near-future | 10 | 13 | 12 | 15 |  | Near-future | 10 | 14 | 11 | 18 |
|  | Far-future | 11 | 11 | 10 | 13 |  | Far-future | 13 | 14 | 14 | 18 |
|  | Time Period | ***Turkmenistan*** | | | |  | Time Period | ***Turkmenistan*** | | | |
|  |  | **TX10p** | **TX90p** | **TN10p** | **TN90p** |  |  | **TX10p** | **TX90p** | **TN10p** | **TN90p** |
|  | Historical 1 | 11 | 10 | 11 | 10 |  | Historical 1 | 11 | 10 | 11 | 10 |
|  | Historical 2 | 8 | 16 | 8 | 16 |  | Historical 2 | 8 | 16 | 8 | 16 |
|  | Near-future | 10 | 13 | 12 | 15 |  | Near-future | 10 | 13 | 11 | 18 |
|  | Far-future | 11 | 11 | 10 | 14 |  | Far-future | 13 | 14 | 14 | 18 |
|  | Time Period | ***Uzbekistan*** | | | |  | Time Period | ***Uzbekistan*** | | | |
|  |  | **TX10p** | **TX90p** | **TN10p** | **TN90p** |  |  | **TX10p** | **TX90p** | **TN10p** | **TN90p** |
|  | Historical 1 | 11 | 11 | 11 | 10 |  | Historical 1 | 11 | 11 | 11 | 10 |
|  | Historical 2 | 8 | 15 | 8 | 15 |  | Historical 2 | 8 | 15 | 8 | 15 |
|  | Near-future | 10 | 13 | 12 | 15 |  | Near-future | 10 | 14 | 11 | 18 |
|  | Far-future | 11 | 11 | 10 | 14 |  | Far-future | 13 | 14 | 14 | 18 |

| **Table S4.** The statistics of calculated climate indices (averaged TX10p, TX90p, TN10p, and TN90p) separately in each cluster of Kazakhstan between 1962 and 2100 under both climate scenarios. | | | | | | | | | | | | | |
| --- | --- | --- | --- | --- | --- | --- | --- | --- | --- | --- | --- | --- | --- |
| **SPSS 2-4.5** |  | ***Kazakhstan*** | | | | | **SPSS 5-8.5** |  | ***Kazakhstan*** | | | | |
|  | **Climate Index** | | **Historical 1** | **Historical 2** | **Near-Future** | **Far-Future** |  | **Climate Index** | | **Historical 1** | **Historical 2** | **Near-Future** | **Far-Future** |
|  | **TX10p** | *Cluster 1* | 11 | 7 | 10 | 11 |  | **TX10p** | *Cluster 1* | 11 | 7 | 10 | 13 |
|  |  | *Cluster 2* | 11 | 9 | 10 | 11 |  |  | *Cluster 2* | 11 | 9 | 10 | 13 |
|  |  | *Cluster 3* | 11 | 8 | 10 | 11 |  |  | *Cluster 3* | 11 | 8 | 10 | 13 |
|  |  | *Cluster 4* | 11 | 8 | 10 | 11 |  |  | *Cluster 4* | 11 | 8 | 10 | 13 |
|  |  | *Cluster 5* | 11 | 9 | 10 | 11 |  |  | *Cluster 5* | 11 | 9 | 10 | 13 |
|  |  | *Cluster 6* | 11 | 7 | 10 | 11 |  |  | *Cluster 6* | 11 | 7 | 10 | 13 |
|  | **TX90p** | *Cluster 1* | 10 | 18 | 13 | 11 |  | **TX90p** | *Cluster 1* | 10 | 18 | 14 | 14 |
|  |  | *Cluster 2* | 10 | 15 | 13 | 11 |  |  | *Cluster 2* | 10 | 15 | 14 | 14 |
|  |  | *Cluster 3* | 10 | 17 | 13 | 11 |  |  | *Cluster 3* | 10 | 17 | 14 | 14 |
|  |  | *Cluster 4* | 10 | 15 | 13 | 11 |  |  | *Cluster 4* | 10 | 15 | 14 | 14 |
|  |  | *Cluster 5* | 10 | 14 | 13 | 11 |  |  | *Cluster 5* | 10 | 14 | 14 | 14 |
|  |  | *Cluster 6* | 10 | 16 | 13 | 11 |  |  | *Cluster 6* | 10 | 16 | 14 | 14 |
|  | **TN10p** | *Cluster 1* | 11 | 7 | 12 | 10 |  | **TN10p** | *Cluster 1* | 11 | 7 | 11 | 14 |
|  |  | *Cluster 2* | 11 | 9 | 12 | 10 |  |  | *Cluster 2* | 11 | 9 | 11 | 14 |
|  |  | *Cluster 3* | 11 | 8 | 12 | 10 |  |  | *Cluster 3* | 11 | 8 | 10 | 14 |
|  |  | *Cluster 4* | 11 | 8 | 12 | 10 |  |  | *Cluster 4* | 11 | 8 | 11 | 14 |
|  |  | *Cluster 5* | 11 | 9 | 12 | 10 |  |  | *Cluster 5* | 11 | 9 | 11 | 14 |
|  |  | *Cluster 6* | 11 | 7 | 12 | 10 |  |  | *Cluster 6* | 11 | 7 | 10 | 14 |
|  | **TN90p** | *Cluster 1* | 10 | 18 | 15 | 14 |  | **TN90p** | *Cluster 1* | 10 | 18 | 18 | 18 |
|  |  | *Cluster 2* | 10 | 15 | 15 | 14 |  |  | *Cluster 2* | 10 | 15 | 18 | 18 |
|  |  | *Cluster 3* | 10 | 17 | 15 | 14 |  |  | *Cluster 3* | 10 | 17 | 18 | 18 |
|  |  | *Cluster 4* | 10 | 15 | 15 | 13 |  |  | *Cluster 4* | 10 | 15 | 18 | 18 |
|  |  | *Cluster 5* | 10 | 14 | 15 | 14 |  |  | *Cluster 5* | 10 | 14 | 18 | 18 |
|  |  | *Cluster 6* | 10 | 16 | 15 | 14 |  |  | *Cluster 6* | 10 | 16 | 18 | 18 |

| **Table S5.** The statistics of calculated climate indices (averaged TX10p, TX90p, TN10p, and TN90p) separately in each cluster of studied countries (except Kazakhstan) between 1962 and 2100 under both climate scenarios. | | | | | | | | | | | | | |
| --- | --- | --- | --- | --- | --- | --- | --- | --- | --- | --- | --- | --- | --- |
| **SPPS 2-4.5** |  | ***Kyrgyzstan*** | | | | | **SPPS 5-8.5** |  | ***Kyrgyzstan*** | | | | |
|  | **Climate Index** | | **Historical 1** | **Historical 2** | **Near-future** | **Far-future** |  | **Climate Index** | | **Historical 1** | **Historical 2** | **Near-future** | **Far-future** |
|  | **TX10p** | *Cluster 1* | 10 | 8 | 10 | 11 |  | **TX10p** | *Cluster 1* | 10 | 8 | 10 | 13 |
|  |  | *Cluster 2* | 11 | 8 | 10 | 11 |  |  | *Cluster 2* | 11 | 8 | 10 | 13 |
|  | **TX90p** | *Cluster 1* | 11 | 15 | 13 | 11 |  | **TX90p** | *Cluster 1* | 11 | 15 | 14 | 14 |
|  |  | *Cluster 2* | 10 | 15 | 13 | 11 |  |  | *Cluster 2* | 10 | 15 | 14 | 14 |
|  | **TN10p** | *Cluster 1* | 10 | 8 | 12 | 10 |  | **TN10p** | *Cluster 1* | 10 | 8 | 11 | 14 |
|  |  | *Cluster 2* | 11 | 8 | 12 | 10 |  |  | *Cluster 2* | 11 | 8 | 11 | 14 |
|  | **TN90p** | *Cluster 1* | 11 | 15 | 15 | 14 |  | **TN90p** | *Cluster 1* | 11 | 15 | 18 | 18 |
|  |  | *Cluster 2* | 10 | 15 | 15 | 13 |  |  | *Cluster 2* | 10 | 15 | 18 | 18 |
|  |  | ***Tajikistan*** | | | | |  |  | ***Tajikistan*** | | | | |
|  | **Climate Index** | | **Historical 1** | **Historical 2** | **Near-future** | **Far-future** |  | **Climate Index** | | **Historical 1** | **Historical 2** | **Near-future** | **Far-future** |
|  | **TX10p** | *Cluster 1* | 11 | 7 | 10 | 11 |  | **TX10p** | *Cluster 1* | 11 | 7 | 10 | 13 |
|  |  | *Cluster 2* | 11 | 8 | 10 | 11 |  |  | *Cluster 2* | 11 | 8 | 10 | 13 |
|  | **TX90p** | *Cluster 1* | 10 | 17 | 13 | 11 |  | **TX90p** | *Cluster 1* | 10 | 17 | 14 | 14 |
|  |  | *Cluster 2* | 11 | 16 | 13 | 11 |  |  | *Cluster 2* | 11 | 16 | 14 | 14 |
|  | **TN10p** | *Cluster 1* | 11 | 7 | 12 | 10 |  | **TN10p** | *Cluster 1* | 11 | 7 | 11 | 14 |
|  |  | *Cluster 2* | 11 | 8 | 12 | 10 |  |  | *Cluster 2* | 11 | 8 | 11 | 14 |
|  | **TN90p** | *Cluster 1* | 10 | 17 | 15 | 13 |  | **TN90p** | *Cluster 1* | 10 | 17 | 18 | 18 |
|  |  | *Cluster 2* | 11 | 16 | 15 | 13 |  |  | *Cluster 2* | 11 | 16 | 19 | 18 |
|  |  | ***Turkmenistan*** | | | | |  |  | ***Turkmenistan*** | | | | |
|  | **Climate Index** | | **Historical 1** | **Historical 2** | **Near-future** | **Far-future** |  | **Climate Index** | | **Historical 1** | **Historical 2** | **Near-future** | **Far-future** |
|  | **TX10p** | *Cluster 1* | 12 | 7 | 10 | 11 |  | **TX10p** | *Cluster 1* | 12 | 7 | 10 | 13 |
|  |  | *Cluster 2* | 11 | 8 | 10 | 11 |  |  | *Cluster 2* | 11 | 8 | 10 | 13 |
|  | **TX90p** | *Cluster 1* | 10 | 17 | 13 | 11 |  | **TX90p** | *Cluster 1* | 10 | 17 | 14 | 14 |
|  |  | *Cluster 2* | 10 | 15 | 13 | 11 |  |  | *Cluster 2* | 10 | 15 | 13 | 14 |
|  | **TN10p** | *Cluster 1* | 12 | 7 | 12 | 10 |  | **TN10p** | *Cluster 1* | 12 | 7 | 11 | 14 |
|  |  | *Cluster 2* | 11 | 8 | 12 | 10 |  |  | *Cluster 2* | 11 | 8 | 10 | 14 |
|  | **TN90p** | *Cluster 1* | 10 | 17 | 15 | 14 |  | **TN90p** | *Cluster 1* | 10 | 17 | 18 | 18 |
|  |  | *Cluster 2* | 10 | 15 | 15 | 14 |  |  | *Cluster 2* | 10 | 15 | 18 | 18 |
|  |  | ***Uzbekistan*** | | | | |  |  | ***Uzbekistan*** | | | | |
|  | **Climate Index** | | **Historical 1** | **Historical 2** | **Near-future** | **Far-future** |  | **Climate Index** | | **Historical 1** | **Historical 2** | **Near-future** | **Far-future** |
|  | **TX10p** | *Cluster 1* | 11 | 8 | 10 | 11 |  | **TX10p** | *Cluster 1* | 11 | 8 | 10 | 13 |
|  |  | *Cluster 2* | 11 | 8 | 10 | 11 |  |  | *Cluster 2* | 11 | 8 | 10 | 13 |
|  | **TX90p** | *Cluster 1* | 11 | 16 | 13 | 11 |  | **TX90p** | *Cluster 1* | 11 | 16 | 14 | 14 |
|  |  | *Cluster 2* | 10 | 16 | 13 | 11 |  |  | *Cluster 2* | 10 | 16 | 14 | 14 |
|  | **TN10p** | *Cluster 1* | 11 | 7 | 12 | 10 |  | **TN10p** | *Cluster 1* | 11 | 7 | 11 | 14 |
|  |  | *Cluster 2* | 11 | 8 | 12 | 10 |  |  | *Cluster 2* | 11 | 8 | 11 | 14 |
|  | **TN90p** | *Cluster 1* | 11 | 16 | 15 | 14 |  | **TN90p** | *Cluster 1* | 11 | 16 | 18 | 18 |
|  |  | *Cluster 2* | 10 | 16 | 15 | 14 |  |  | *Cluster 2* | 10 | 16 | 18 | 19 |

**
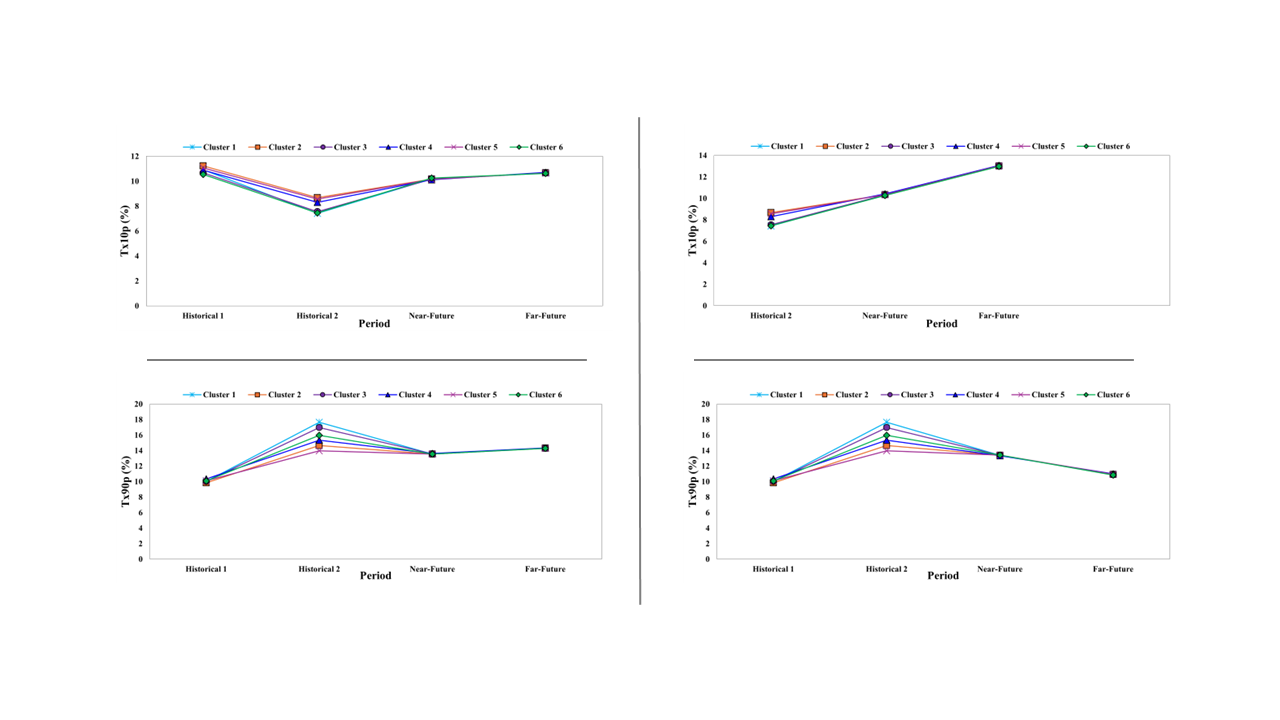

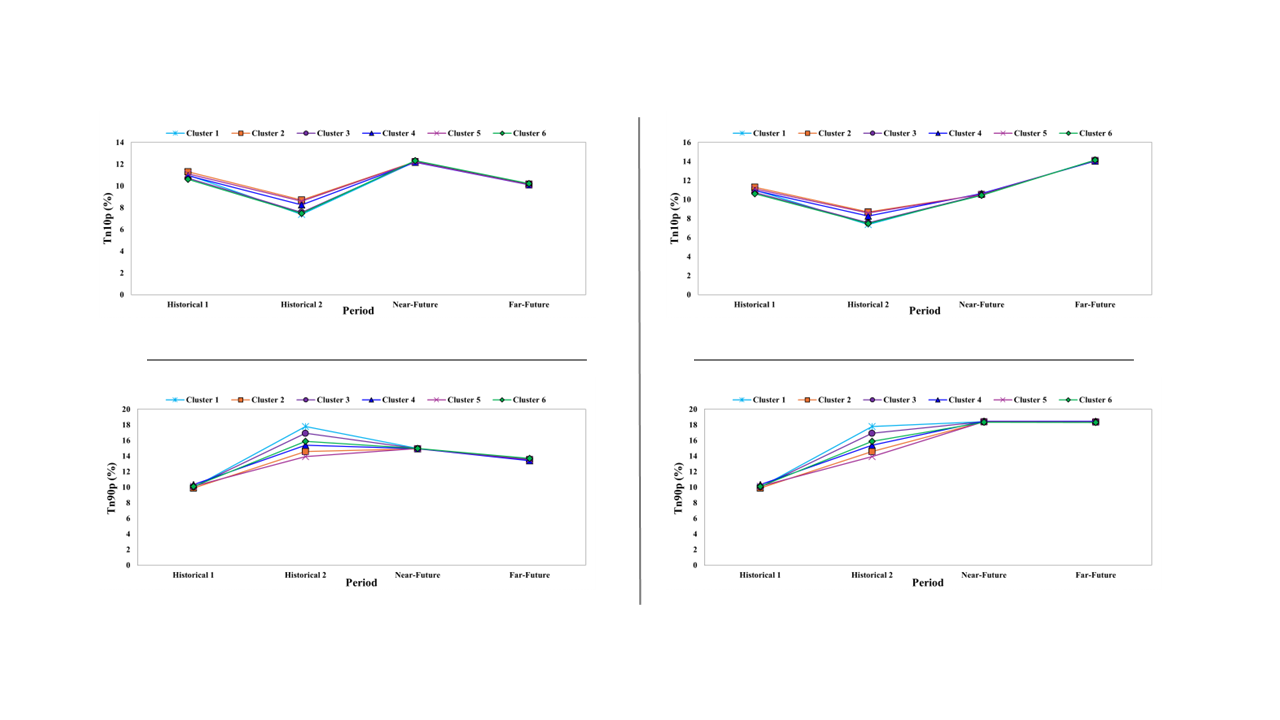
**

**Fig. S2.** The temporal changes in the statistics of averaged TN10p, TN90p, TX10p, and TX90p, separately in Kazakhstan between 1962 and 2100 under (**Left**) SSP2–4.5 and (**Right**) SSP5–8.5.


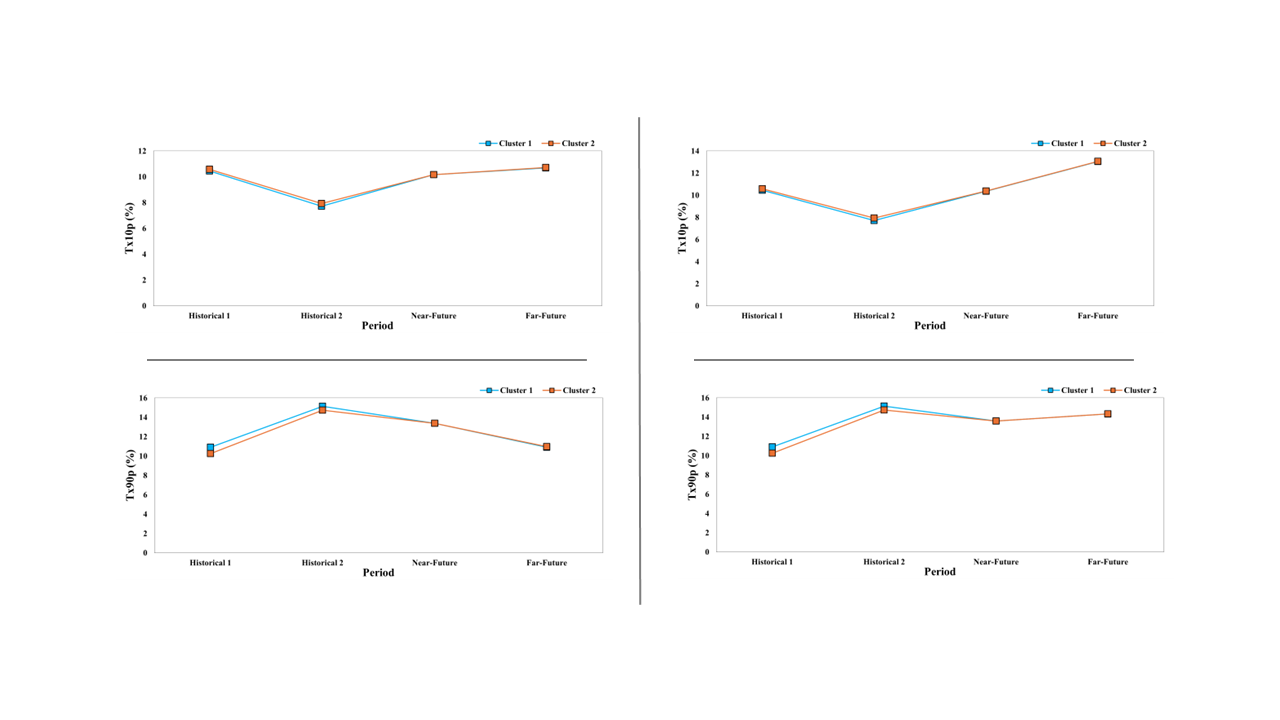

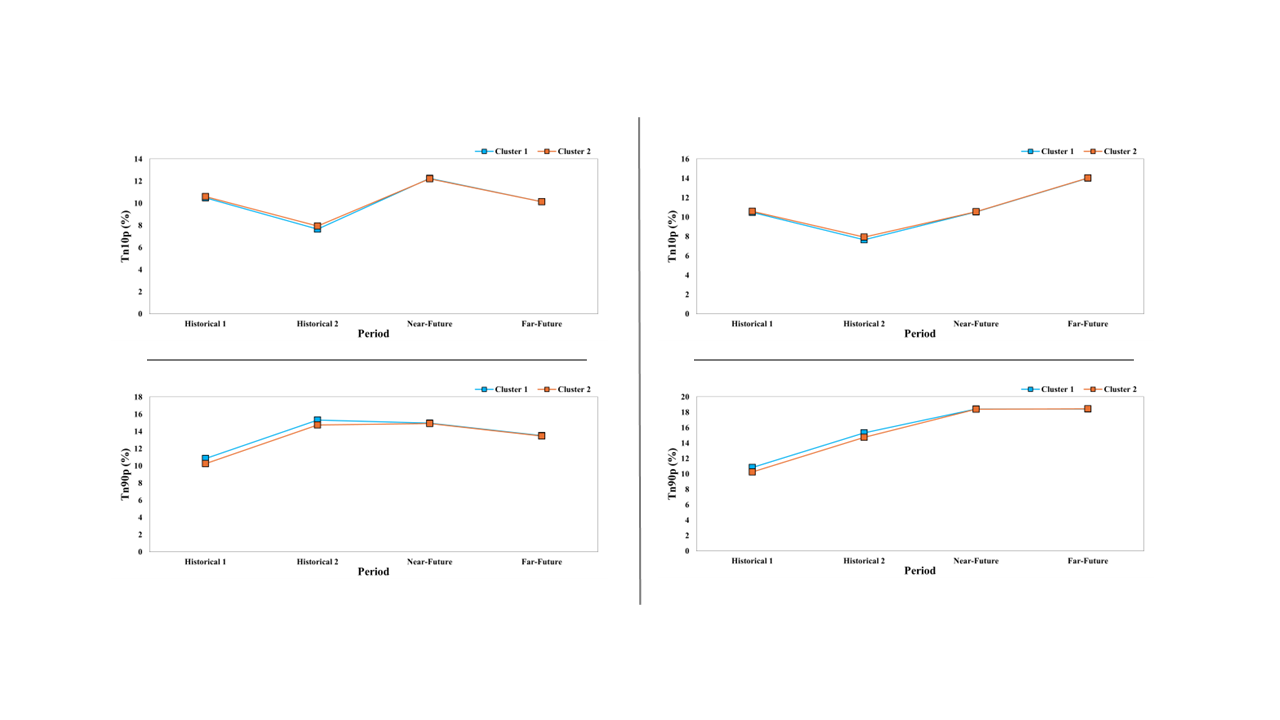


**Fig. S3.** The temporal changes in the statistics of averaged TN10p, TN90p, TX10p, and TX90p, separately in Kyrgystan between 1962 and 2100 under (**Left**) SSP2–4.5 and (**Right**) SSP5–8.5.


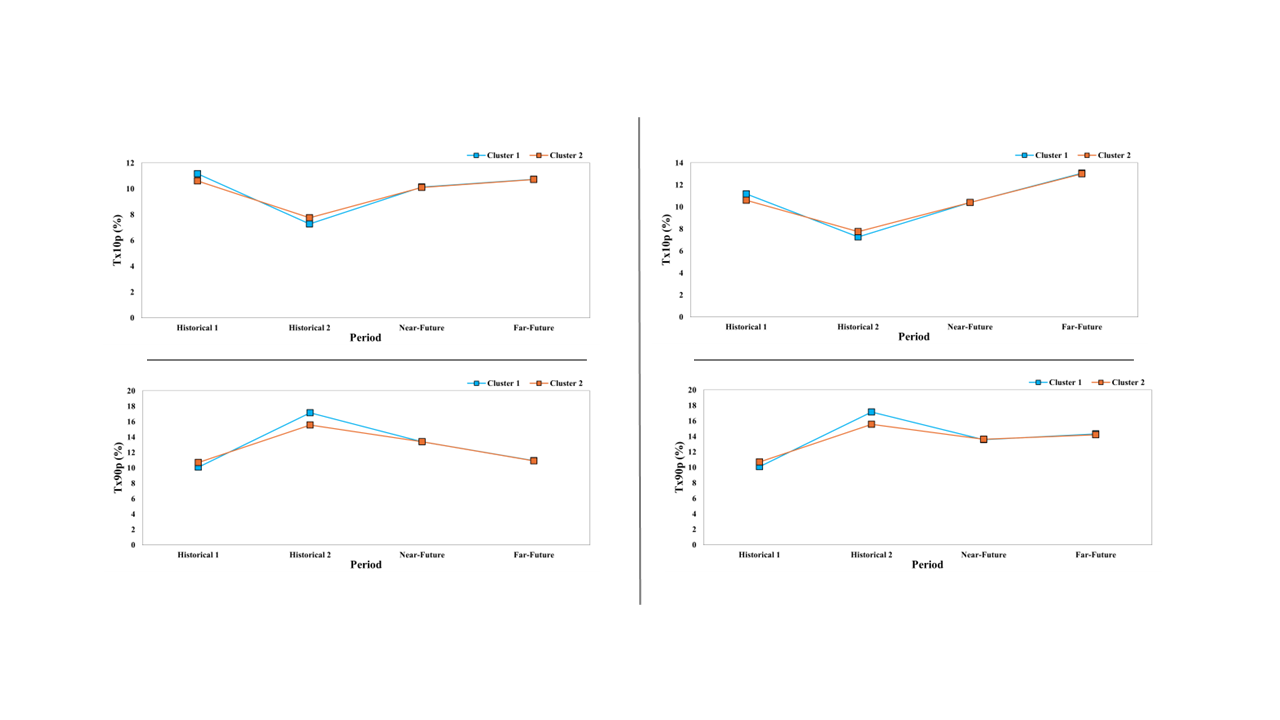

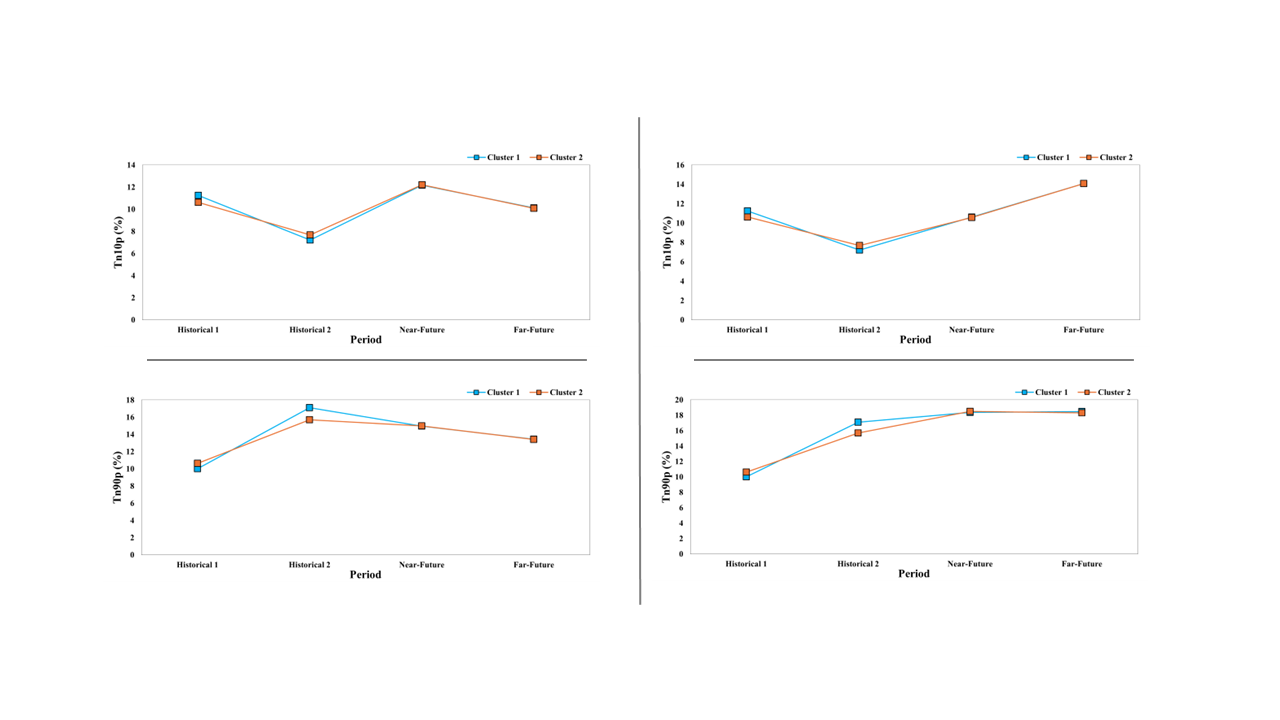
**Fig. S4.** The temporal changes in the statistics of averaged TN10p, TN90p, TX10p, and TX90p, separately in Tajikistan between 1962 and 2100 under (**Left**) SSP2–4.5 and (**Right**) SSP5–8.5.


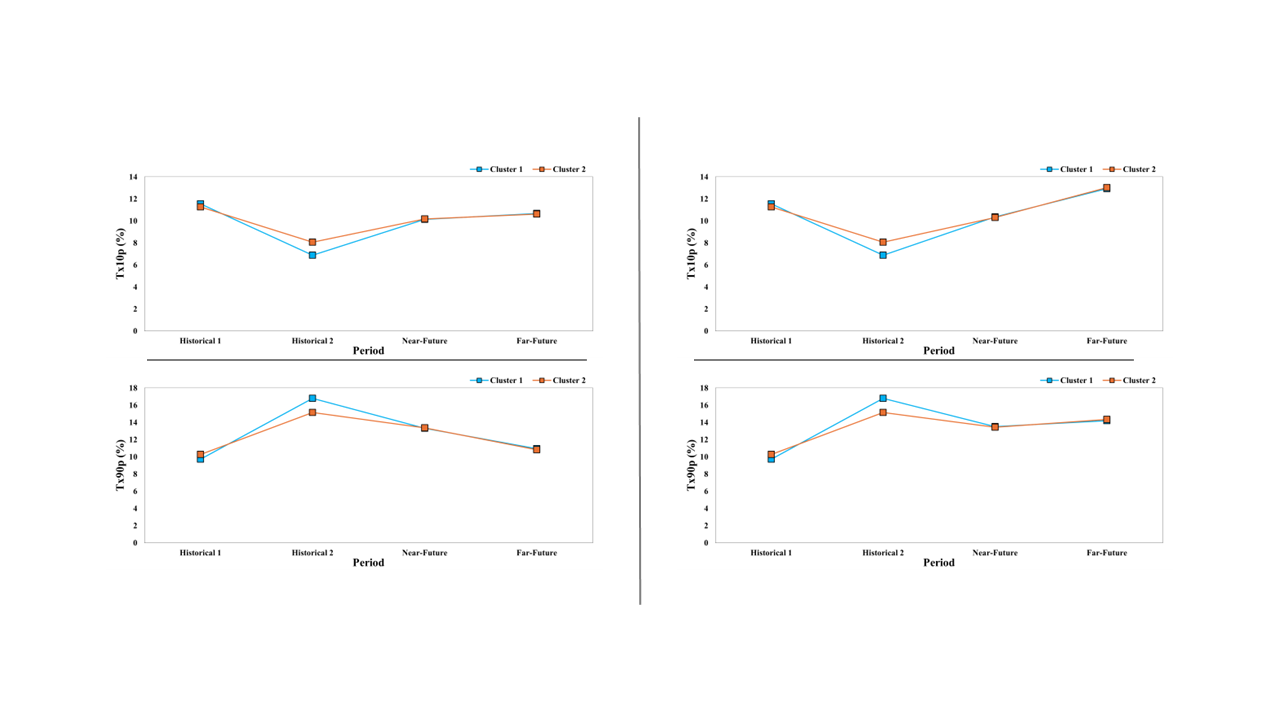


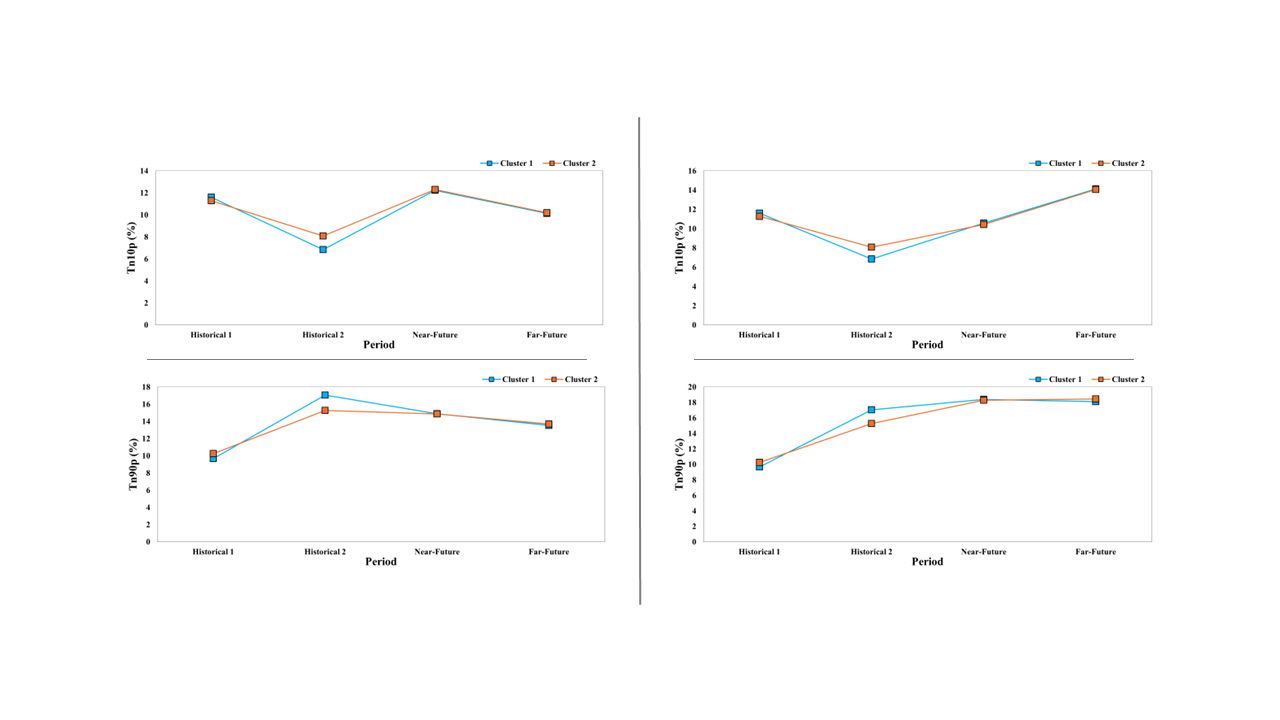
**Fig. S5.** The temporal changes in the statistics of averaged TN10p, TN90p, TX10p, and TX90p, separately in Turkmenistan between 1962 and 2100 under (**Left**) SSP2–4.5 and (**Right**) SSP5–8.5.


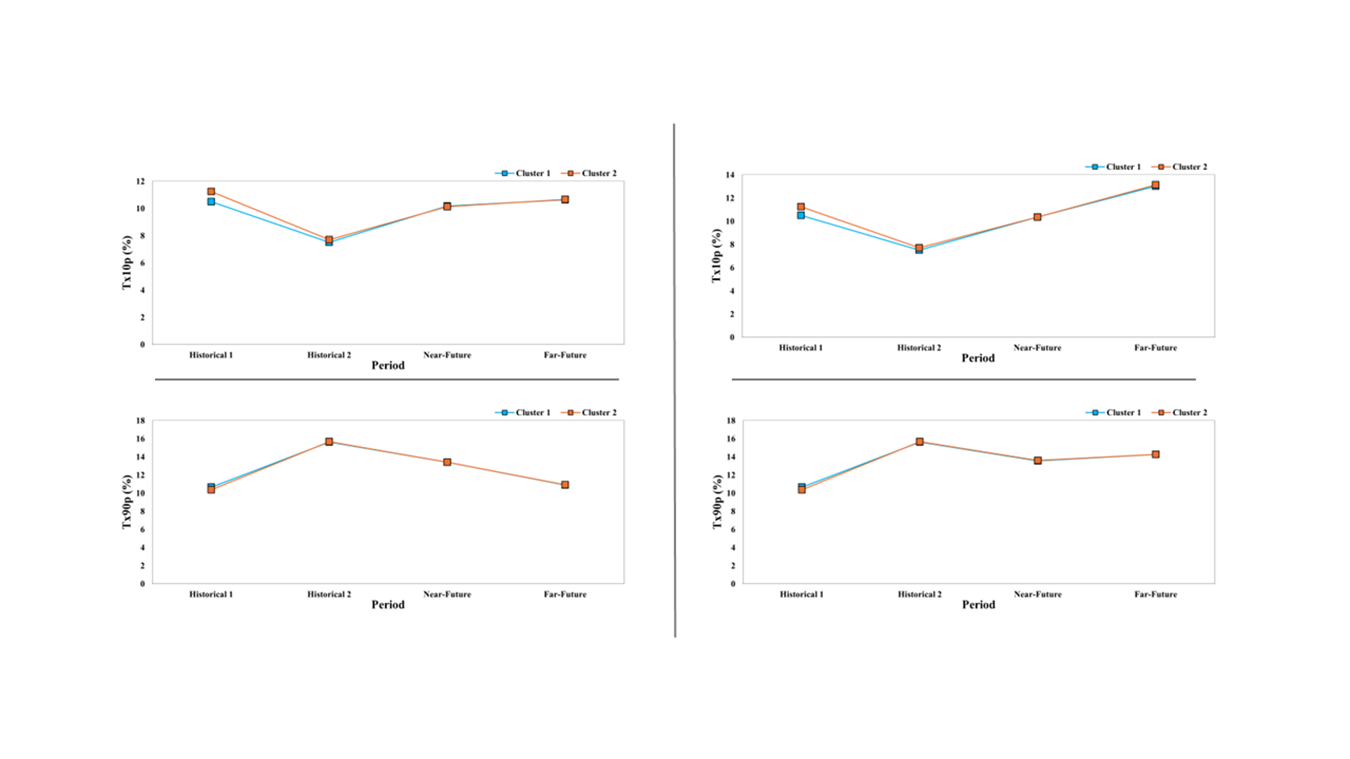

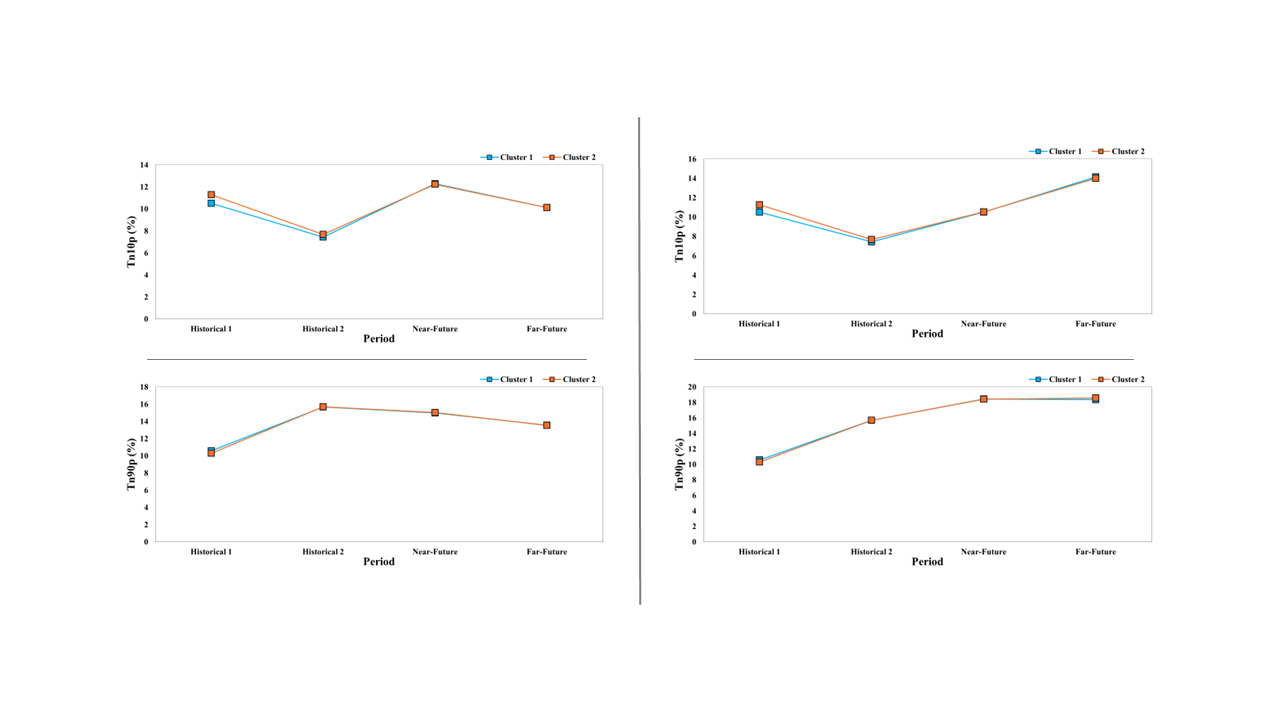


**Fig. S6.** The temporal changes in the statistics of averaged TN10p, TN90p, TX10p, and TX90p, separately in Uzbekistan between 1962 and 2100 under (**Left**) SSP2–4.5 and (**Right**) SSP5–8.5.

**Fig. S7.** The slope of spatial-temporal changes of (**1**) cool days (TX10p), (**2**) cool nights (TN10p), (**3**) warm days (TX90p), and (**4**) warm nights (TN90p) in CA, considering SSP2–4.5 and SSP5–8.5 climate projections between 1962 and 2100.
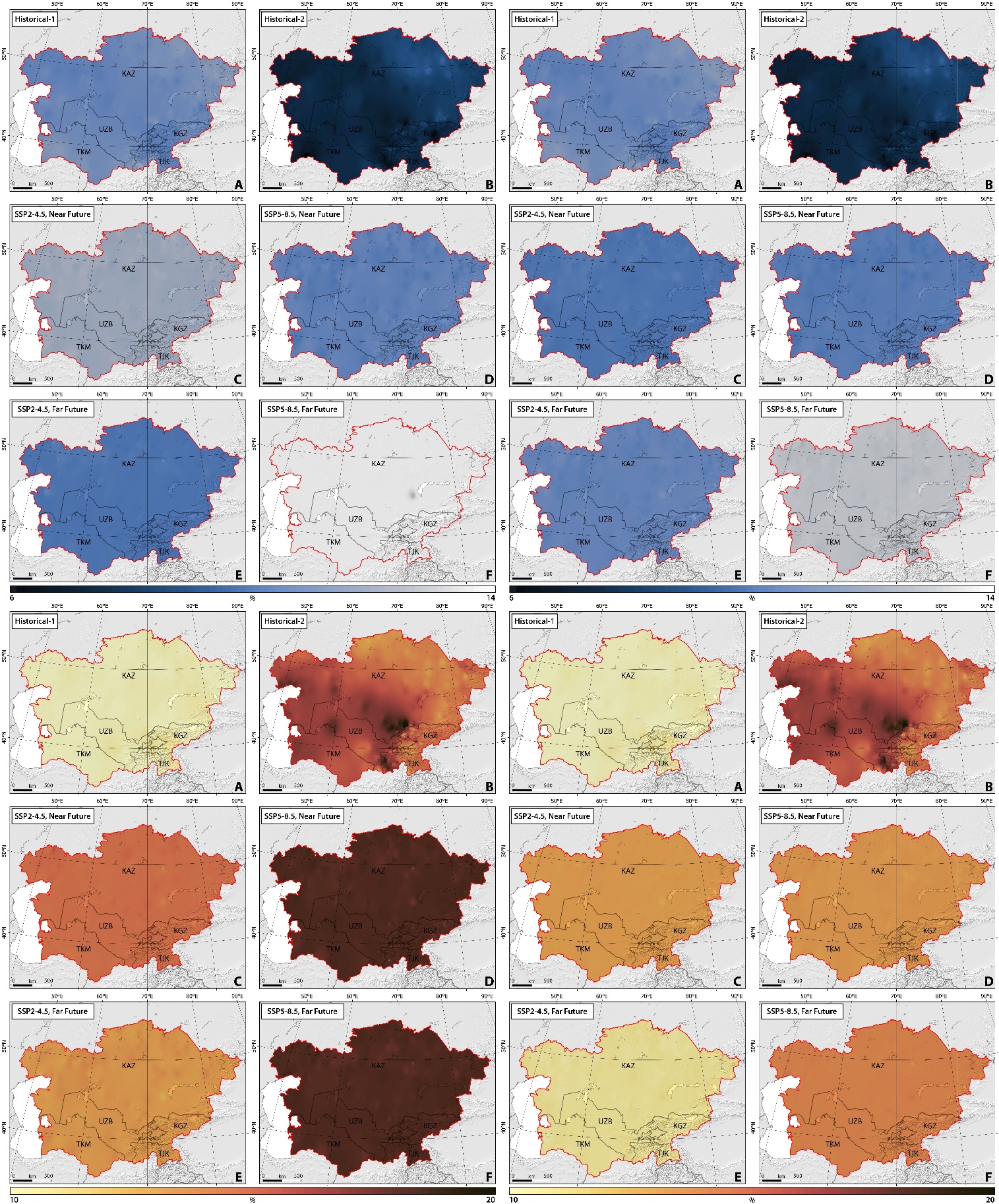


**4**

**3**

**2**

**1**

| **Table S6.** The statistics of calculated climate indices (summed FD, ID, SU, and TR) separately in studied countries between 1962 and 2100 under both climate scenarios. | | | | | | | | | | | |
| --- | --- | --- | --- | --- | --- | --- | --- | --- | --- | --- | --- |
| SS**P2–4.5** | *Time Period* | ***Kazakhstan*** | | | | **SSP5–8.5** | *Time Period* | ***Kazakhstan*** | | | |
|  |  | **FD** | **ID** | **SU** | **TR** |  |  | **FD** | **ID** | **SU** | **TR** |
|  | *Historical 1* | 1.26×10^6^ | 1.22×10^6^ | 3.20×10^5^ | 7.11×10^5^ |  | *Historical 1* | 1.26×10^6^ | 1.22×10^6^ | 3.20×10^5^ | 7.11×10^5^ |
|  | *Historical 2* | 1.16×10^6^ | 1.12×10^6^ | 3.71×10^5^ | 7.60×10^5^ |  | *Historical 2* | 1.16×10^6^ | 1.12×10^6^ | 3.71×10^5^ | 7.60×10^5^ |
|  | *Near-future* | 7.61×10^5^ | 8.35×10^5^ | 4.68×10^5^ | 8.02×10^5^ |  | *Near-future* | 7.78×10^5^ | 8.33×10^5^ | 5.70×10^5^ | 8.18×10^5^ |
|  | *Far-future* | 6.23×10^5^ | 7.48×10^5^ | 6.50×10^5^ | 9.53×10^5^ |  | *Far-future* | 2.87×10^5^ | 5.17×10^5^ | 7.98×10^5^ | 1.08×10^6^ |
|  | *Time Period* | ***Kyrgyzstan*** | | | |  | *Time Period* | ***Kyrgyzstan*** | | | |
|  |  | **FD** | **ID** | **SU** | **TR** |  |  | **FD** | **ID** | **SU** | **TR** |
|  | *Historical 1* | 6.27×10^5^ | 5.83×10^5^ | 9.66×10^4^ | 2.79×10^5^ |  | *Historical 1* | 6.27×10^5^ | 5.83×10^5^ | 9.66×10^4^ | 2.79×10^5^ |
|  | *Historical 2* | 5.72×10^5^ | 5.25×10^5^ | 1.01×10^5^ | 2.94×10^5^ |  | *Historical 2* | 5.72×10^5^ | 5.25×10^5^ | 1.01×10^5^ | 2.94×10^5^ |
|  | *Near-future* | 5.41×10^5^ | 5.89×10^5^ | 3.26×10^5^ | 5.61×10^5^ |  | *Near-future* | 5.54×10^5^ | 5.87×10^5^ | 3.99×10^5^ | 5.72×10^5^ |
|  | *Far-future* | 4.44×10^5^ | 5.26×10^5^ | 4.56×10^5^ | 6.69×10^5^ |  | *Far-future* | 2.10×10^5^ | 3.73×10^5^ | 6.04×10^5^ | 8.19×10^5^ |
|  | *Time Period* | ***Tajikistan*** | | | |  | *Time Period* | ***Tajikistan*** | | | |
|  |  | **FD** | **ID** | **SU** | **TR** |  |  | **FD** | **ID** | **SU** | **TR** |
|  | *Historical 1* | 1.57×10^5^ | 1.43×10^5^ | 1.00×10^5^ | 1.87×10^5^ |  | *Historical 1* | 1.57×10^5^ | 1.43×10^5^ | 1.00×10^5^ | 1.87×10^5^ |
|  | *Historical 2* | 1.32×10^5^ | 1.19×10^5^ | 1.14×10^5^ | 2.03×10^5^ |  | *Historical 2* | 1.32×10^5^ | 1.19×10^5^ | 1.14×10^5^ | 2.03×10^5^ |
|  | *Near-future* | 1.61×10^5^ | 1.78×10^5^ | 1.05×10^5^ | 1.80×10^5^ |  | *Near-future* | 1.65×10^5^ | 1.78×10^5^ | 1.26×10^5^ | 1.83×10^5^ |
|  | *Far-future* | 1.31×10^5^ | 1.59×10^5^ | 1.44×10^5^ | 2.13×10^5^ |  | *Far-future* | 5.83×10^4^ | 1.09×10^5^ | 1.75×10^5^ | 2.37×10^5^ |
|  | *Time Period* | ***Turkmenistan*** | | | |  | *Time Period* | ***Turkmenistan*** | | | |
|  |  | **FD** | **ID** | **SU** | **TR** |  |  | **FD** | **ID** | **SU** | **TR** |
|  | *Historical 1* | 5.43×10^3^ | 4.74×10^3^ | 2.86×10^4^ | 3.77×10^4^ |  | *Historical 1* | 5.43×10^3^ | 4.74×10^3^ | 2.86×10^4^ | 3.77×10^4^ |
|  | *Historical 2* | 3.01×10^3^ | 2.45×10^3^ | 3.06×10^4^ | 3.98×10^4^ |  | *Historical 2* | 3.01×10^3^ | 2.45×10^3^ | 3.06×10^4^ | 3.98×10^4^ |
|  | *Near-future* | 2.35×10^4^ | 2.59×10^4^ | 1.43×10^4^ | 2.45×10^4^ |  | *Near-future* | 2.41×10^4^ | 2.58×10^4^ | 1.75×10^4^ | 2.50×10^4^ |
|  | *Far-future* | 1.93×10^4^ | 2.32×10^4^ | 1.99×10^4^ | 2.91×10^4^ |  | *Far-future* | 7.86×10^3^ | 1.44×10^4^ | 2.20×10^4^ | 2.98×10^4^ |
|  | *Time Period* | ***Uzbekistan*** | | | |  | *Time Period* | ***Uzbekistan*** | | | |
|  |  | **FD** | **ID** | **SU** | **TR** |  |  | **FD** | **ID** | **SU** | **TR** |
|  | *Historical 1* | 3.13×10^4^ | 2.76×10^4^ | 5.44×10^4^ | 8.60×10^4^ |  | *Historical 1* | 3.13×10^4^ | 2.76×10^4^ | 5.44×10^4^ | 8.60×10^4^ |
|  | *Historical 2* | 2.39×10^4^ | 1.98×10^4^ | 5.91×10^4^ | 9.03×10^4^ |  | *Historical 2* | 2.39×10^4^ | 1.98×10^4^ | 5.91×10^4^ | 9.03×10^4^ |
|  | *Near-future* | 6.72×10^4^ | 7.41×10^4^ | 4.16×10^4^ | 7.07×10^4^ |  | *Near-future* | 6.87×10^4^ | 7.39×10^4^ | 5.05×10^4^ | 7.21×10^4^ |
|  | *Far-future* | 5.49×10^4^ | 6.63×10^4^ | 5.74×10^4^ | 8.40×10^4^ |  | *Far-future* | 3.14×10^4^ | 5.74×10^4^ | 8.83×10^4^ | 1.20×10^5^ |

**
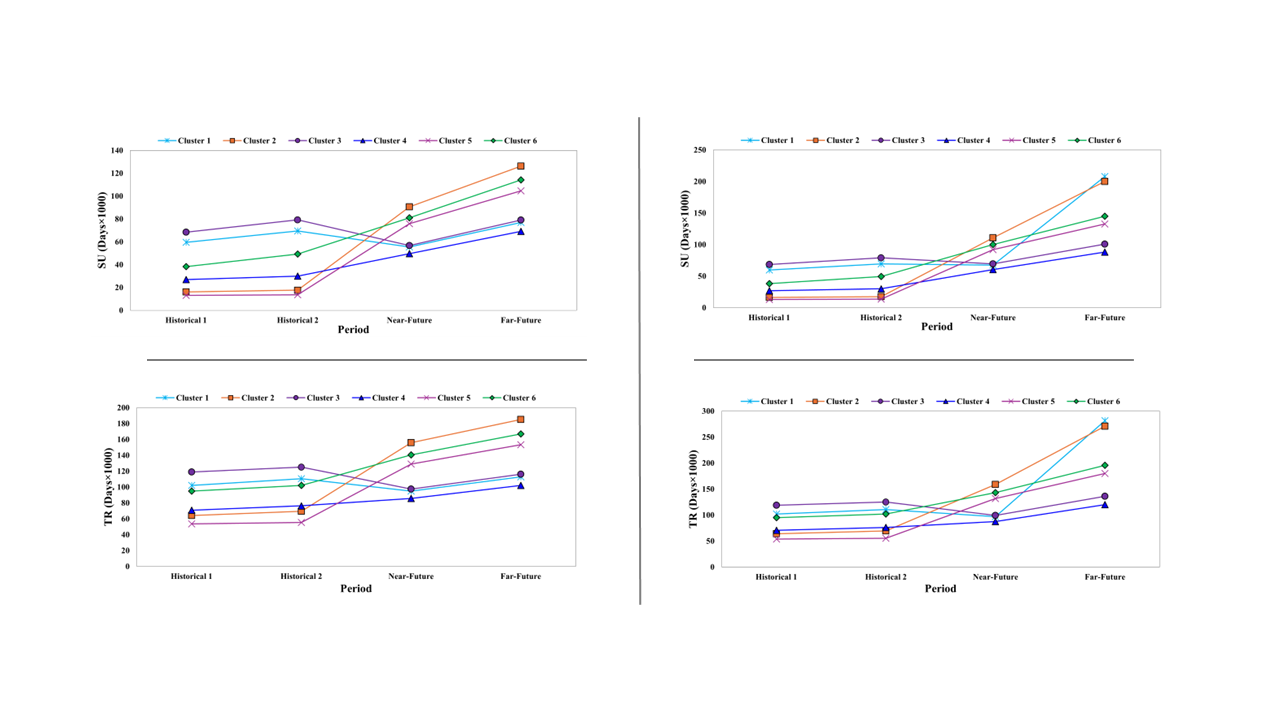
**
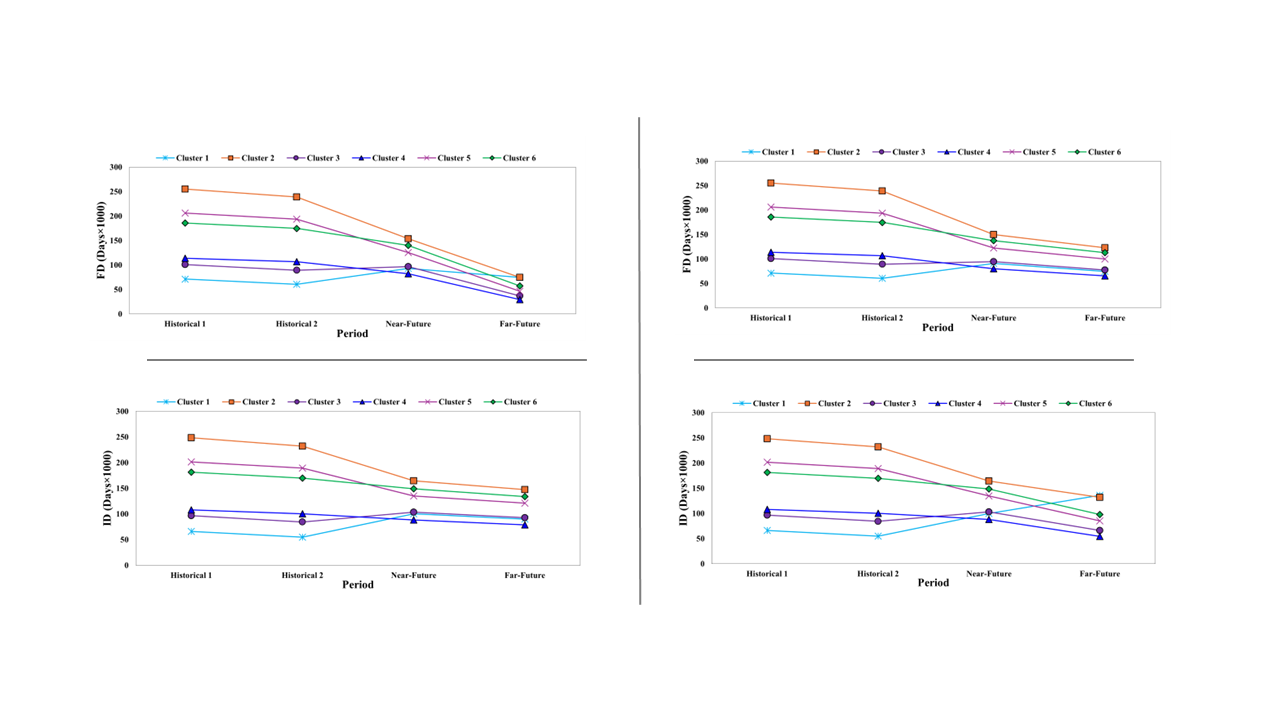


**Fig. S8.** The temporal changes in the statistics of averaged FD, ID, SU, and TR, separately in Kazakhstan between 1962 and 2100 under (**Left**) SSP2–4.5 and (**Right**) SSP5–8.5.


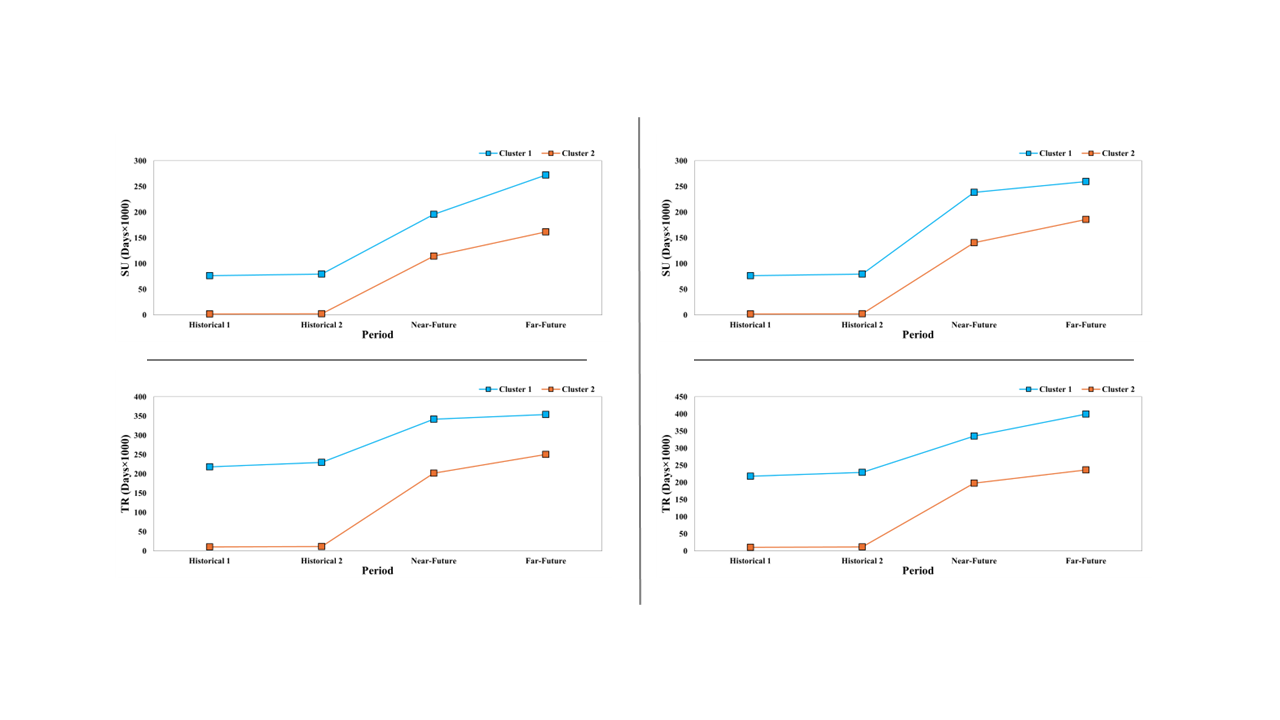
**Fig. S9.** The temporal changes in the statistics of averaged FD, ID, SU, and TR, separately in Kyrgystan between 1962 and 2100 under (**Left**) SSP2–4.5 and (**Right**) SSP5–8.5.
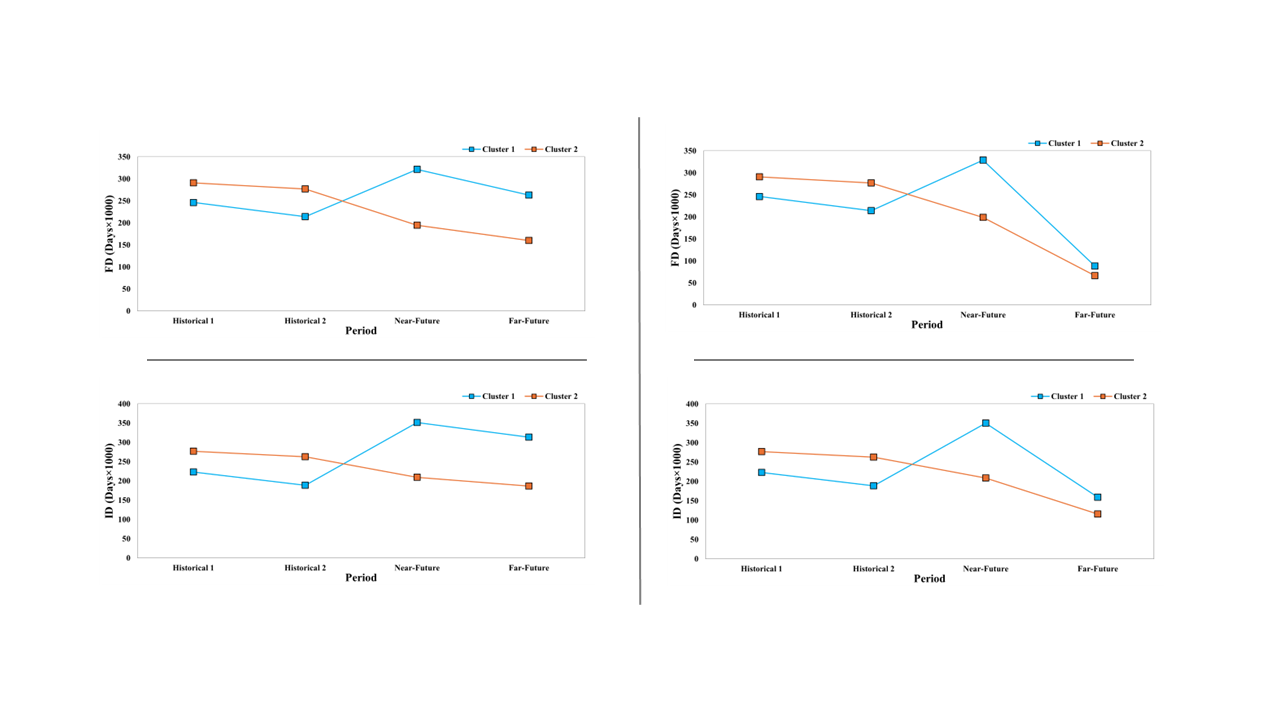


**Fig. S10.** The temporal changes in the statistics of averaged FD, ID, SU, and TR, separately in Tajikistan between 1962 and 2100 under (**Left**) SSP2–4.5 and (**Right**) SSP5–8.5
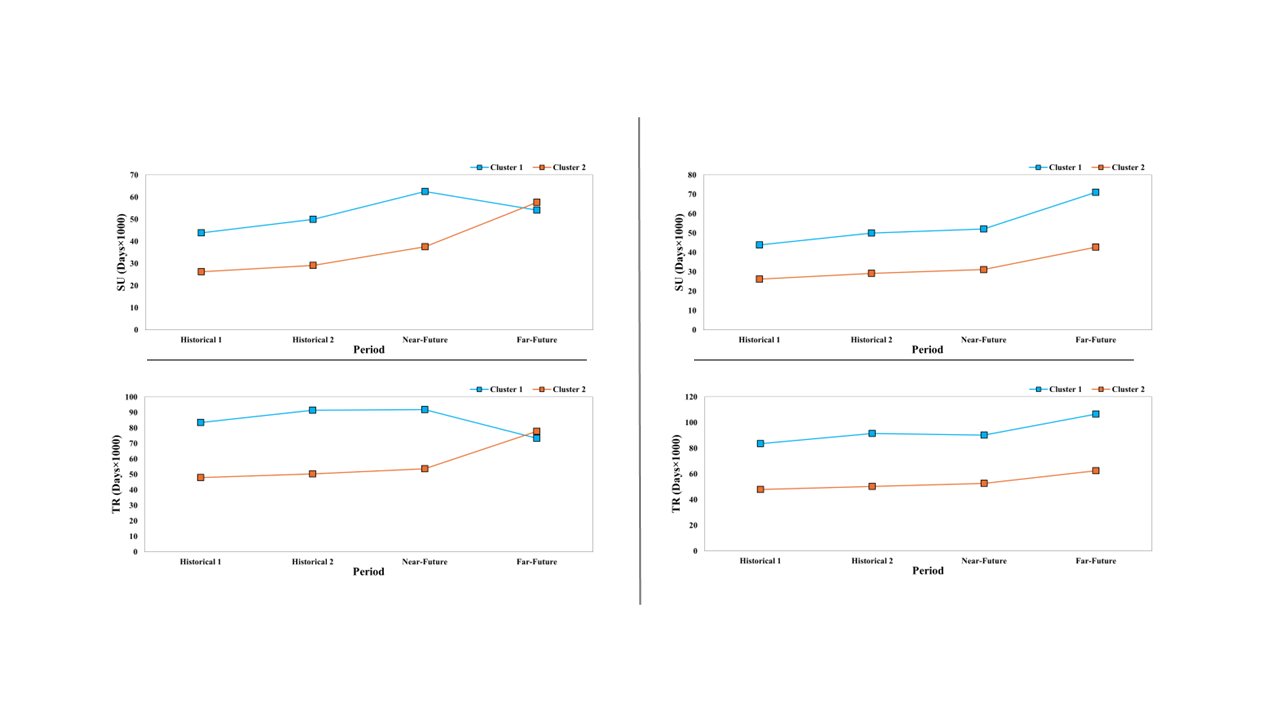

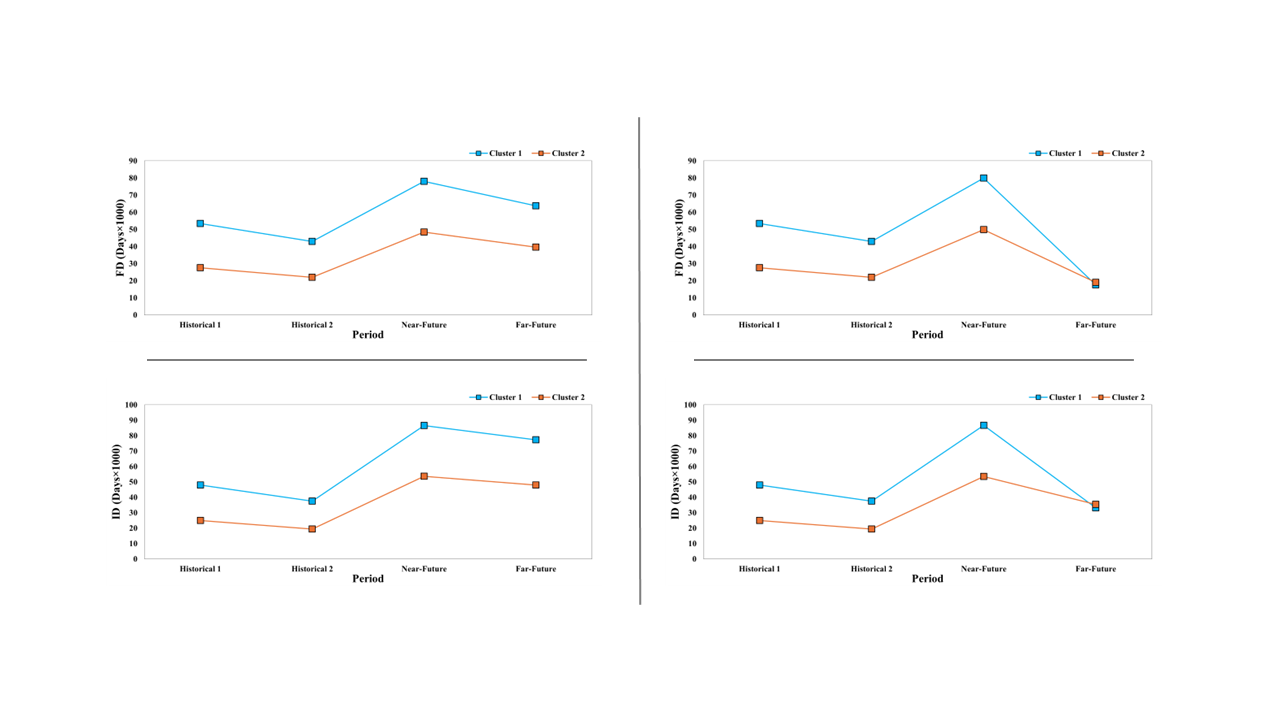
.

**Fig. S11.** The temporal changes in the statistics of averaged FD, ID, SU, and TR, separately in Turkmenistan between 1962 and 2100 under (**Left**) SSP2–4.5 and (**Right**) SSP5–8.5
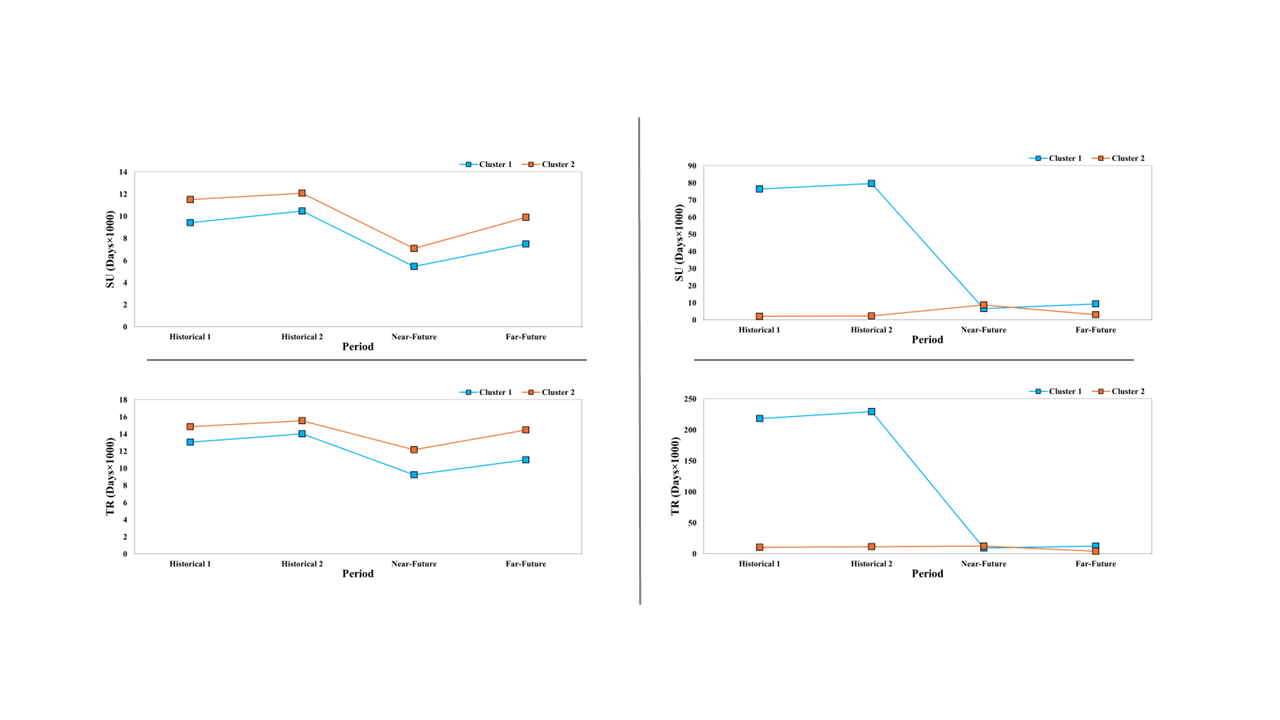

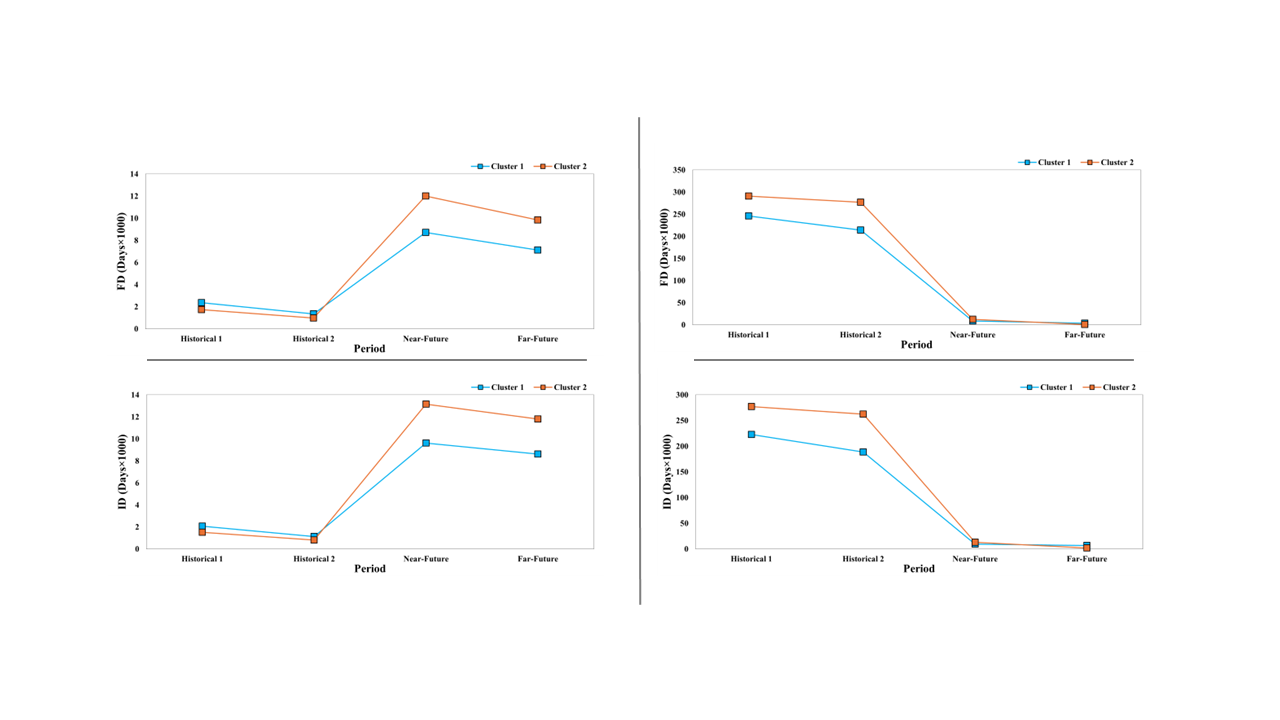
.


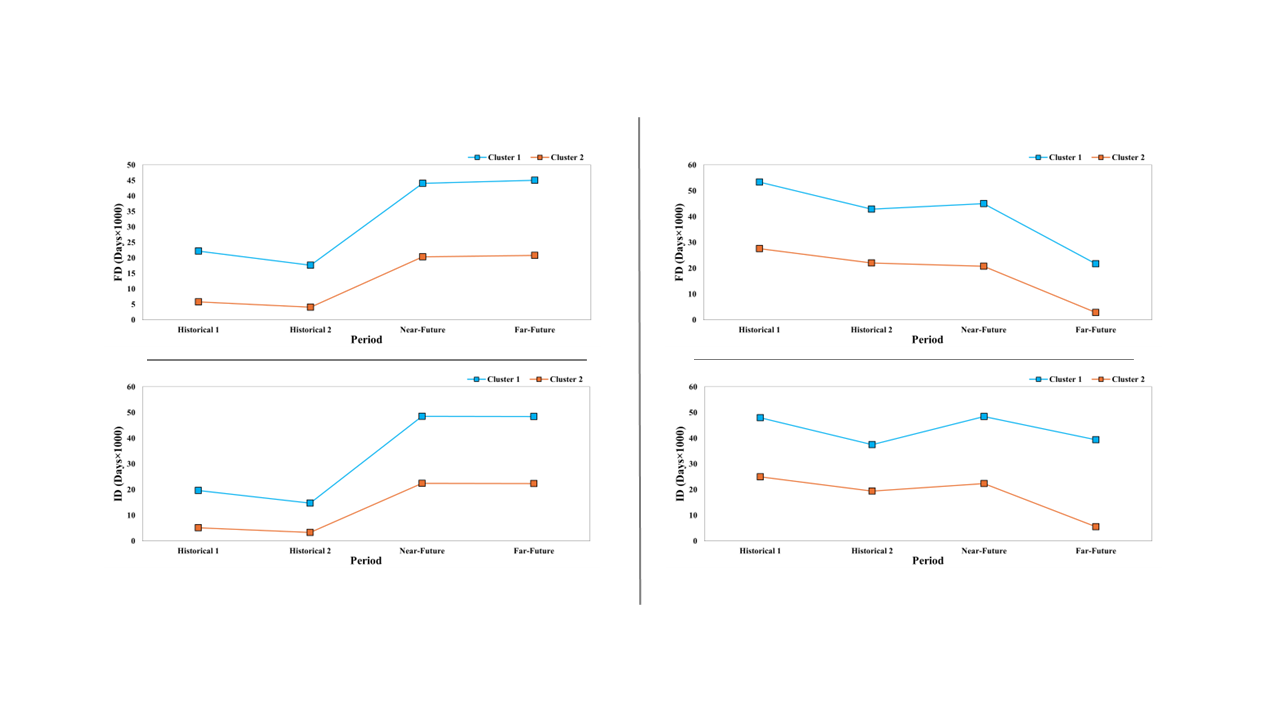

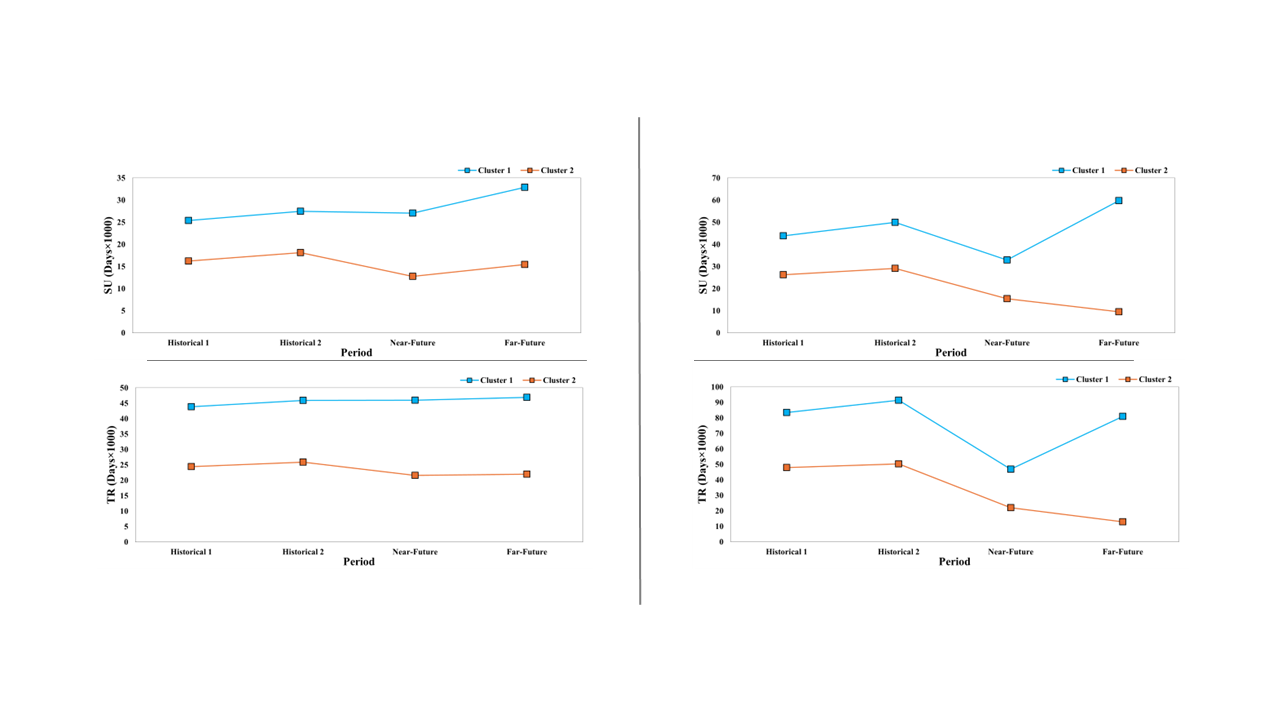

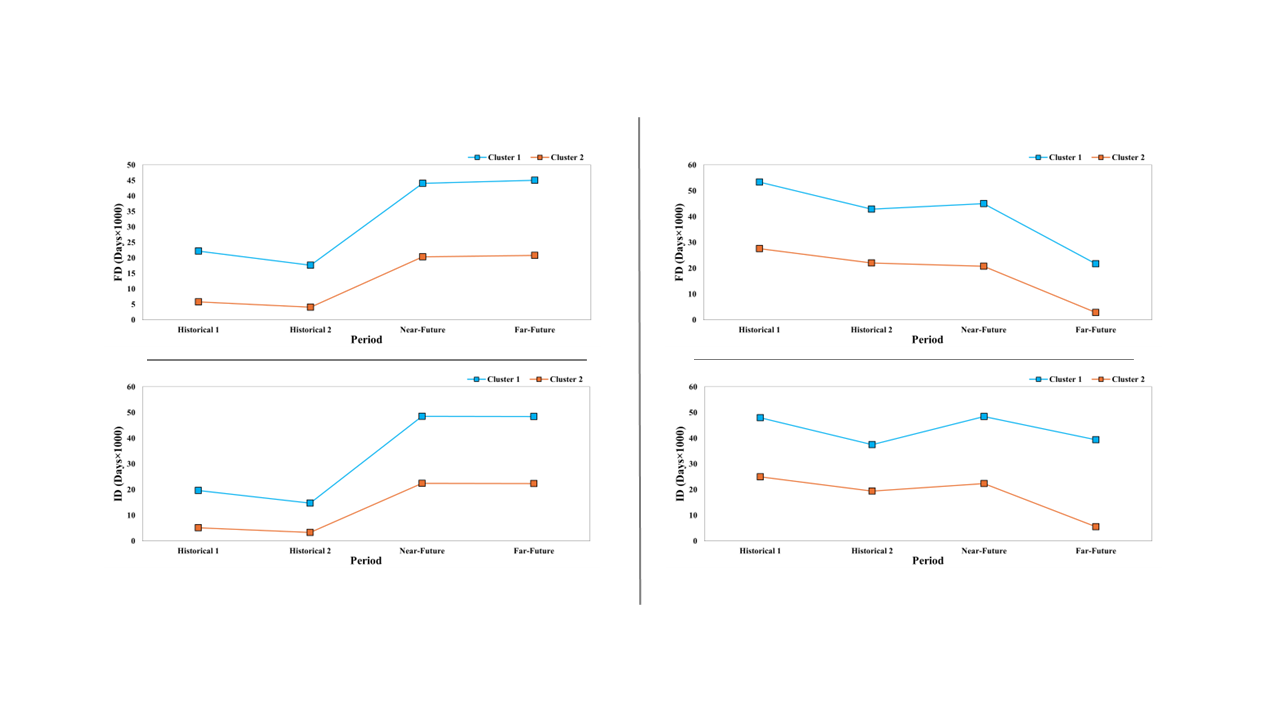


**Fig. S12.** The temporal changes in the statistics of averaged FD, ID, SU, and TR, separately in Uzbekistan between 1962 and 2100 under (**Left**) SSP2–4.5 and (**Right**) SSP5–8.5.


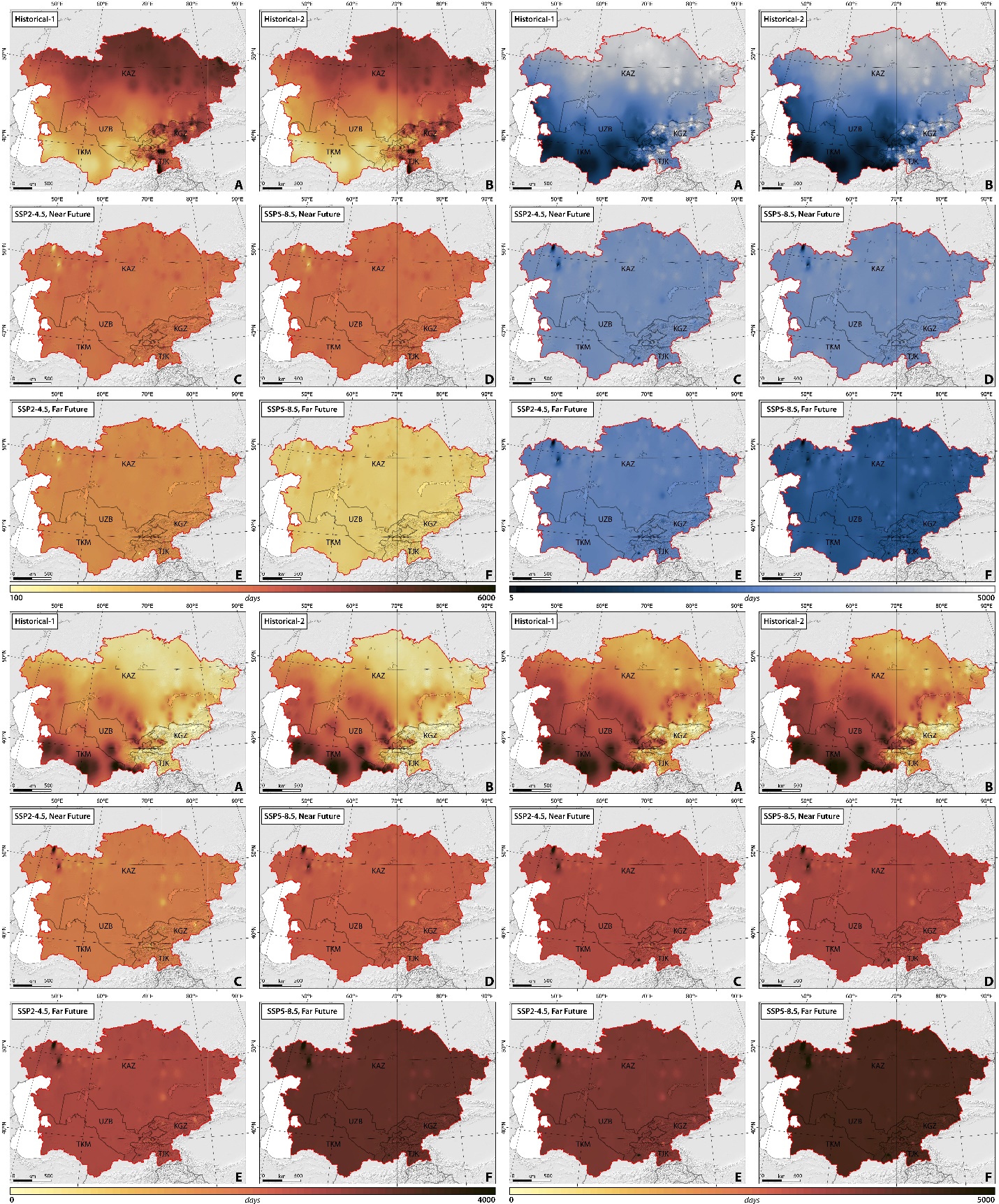


4

3

2

1

**Fig. S13.** The slope of spatial-temporal changes of (**1**) FD, (**2**) ID, (**3**) SU, and (**4**) TR in CA, considering SSP2–4.5 and SSP5–8.5 climate projections between 1962 and 2100.

| **Table S7.** The statistics of calculated climate indices (averaged FD, ID, SU, and TR) separately in each cluster of Kazakhstan between 1962 and 2100 under both climate scenarios. | | | | | | | | | | | | | |
| --- | --- | --- | --- | --- | --- | --- | --- | --- | --- | --- | --- | --- | --- |
| **SPSS 2-4.5** |  | ***Kazakhstan*** | | | | | **SPSS 5-8.5** |  | ***Kazakhstan*** | | | | |
|  | **Climate Index** | | **Historical 1** | **Historical 2** | **Near-future** | **Far-future** |  | **Climate Index** | | **Historical 1** | **Historical 2** | **Near-future** | **Far-future** |
|  | **FD** | *Cluster 1* | 7.12×10^4^ | 6.05×10^4^ | 9.12×10^4^ | 7.46×10^4^ |  | **FD** | *Cluster 1* | 7.12×10^4^ | 6.05×10^4^ | 9.32×10^4^ | 7.49×10^4^ |
|  |  | *Cluster 2* | 2.55×10^5^ | 2.39×10^5^ | 1.50×10^5^ | 1.23×10^5^ |  |  | *Cluster 2* | 2.55×10^5^ | 2.39×10^5^ | 1.54×10^5^ | 7.47×10^4^ |
|  |  | *Cluster 3* | 1.01×10^5^ | 8.94×10^4^ | 9.46×10^4^ | 7.75×10^4^ |  |  | *Cluster 3* | 1.01×10^5^ | 8.94×10^4^ | 9.67×10^4^ | 3.71×10^4^ |
|  |  | *Cluster 4* | 1.14×10^5^ | 1.07×10^5^ | 8.02×10^4^ | 6.54×10^4^ |  |  | *Cluster 4* | 1.14×10^5^ | 1.07×10^5^ | 8.23×10^4^ | 2.94×10^4^ |
|  |  | *Cluster 5* | 2.06×10^5^ | 1.93×10^5^ | 1.23×10^5^ | 1.00×10^5^ |  |  | *Cluster 5* | 2.06×10^5^ | 1.93×10^5^ | 1.25×10^5^ | 4.65×10^4^ |
|  |  | *Cluster 6* | 1.86×10^5^ | 1.75×10^5^ | 1.38×10^5^ | 1.13×10^5^ |  |  | *Cluster 6* | 1.86×10^5^ | 1.75×10^5^ | 1.40×10^5^ | 5.74×10^4^ |
|  | **ID** | *Cluster 1* | 6.61×10^4^ | 5.49×10^4^ | 1.00×10^5^ | 8.98×10^4^ |  | **ID** | *Cluster 1* | 6.61×10^4^ | 5.49×10^4^ | 1.00×10^5^ | 1.36×10^5^ |
|  |  | *Cluster 2* | 2.48×10^5^ | 2.32×10^5^ | 1.65×10^5^ | 1.47×10^5^ |  |  | *Cluster 2* | 2.48×10^5^ | 2.32×10^5^ | 1.64×10^5^ | 1.32×10^5^ |
|  |  | *Cluster 3* | 9.68×10^4^ | 8.45×10^4^ | 1.04×10^5^ | 9.27×10^4^ |  |  | *Cluster 3* | 9.68×10^4^ | 8.45×10^4^ | 1.03×10^5^ | 6.61×10^4^ |
|  |  | *Cluster 4* | 1.08×10^5^ | 1.00×10^5^ | 8.83×10^4^ | 7.89×10^4^ |  |  | *Cluster 4* | 1.08×10^5^ | 1.00×10^5^ | 8.81×10^4^ | 5.44×10^4^ |
|  |  | *Cluster 5* | 2.02×10^5^ | 1.89×10^5^ | 1.35×10^5^ | 1.21×10^5^ |  |  | *Cluster 5* | 2.02×10^5^ | 1.89×10^5^ | 1.35×10^5^ | 8.55×10^4^ |
|  |  | *Cluster 6* | 1.81×10^5^ | 1.70×10^5^ | 1.49×10^5^ | 1.34×10^5^ |  |  | *Cluster 6* | 1.81×10^5^ | 1.70×10^5^ | 1.49×10^5^ | 9.77×10^4^ |
|  | **SU** | *Cluster 1* | 5.96×10^4^ | 6.96×10^4^ | 5.55×10^4^ | 7.69×10^4^ |  | **SU** | *Cluster 1* | 5.96×10^4^ | 6.96×10^4^ | 6.76×10^4^ | 2.08×10^5^ |
|  |  | *Cluster 2* | 1.61×10^4^ | 1.76×10^4^ | 9.06×10^4^ | 1.26×10^5^ |  |  | *Cluster 2* | 1.61×10^4^ | 1.76×10^4^ | 1.11×10^5^ | 2.00×10^5^ |
|  |  | *Cluster 3* | 6.85×10^4^ | 7.92×10^4^ | 5.67×10^4^ | 7.91×10^4^ |  |  | *Cluster 3* | 6.85×10^4^ | 7.92×10^4^ | 6.94×10^4^ | 1.01×10^5^ |
|  |  | *Cluster 4* | 2.69×10^4^ | 2.99×10^4^ | 4.96×10^4^ | 6.92×10^4^ |  |  | *Cluster 4* | 2.69×10^4^ | 2.99×10^4^ | 6.04×10^4^ | 8.83×10^4^ |
|  |  | *Cluster 5* | 1.32×10^4^ | 1.35×10^4^ | 7.58×10^4^ | 1.05×10^5^ |  |  | *Cluster 5* | 1.32×10^4^ | 1.35×10^4^ | 9.20×10^4^ | 1.33×10^5^ |
|  |  | *Cluster 6* | 3.83×10^4^ | 4.93×10^4^ | 8.10×10^4^ | 1.14×10^5^ |  |  | *Cluster 6* | 3.83×10^4^ | 4.93×10^4^ | 1.00×10^5^ | 1.45×10^5^ |
|  | **TR** | *Cluster 1* | 1.02×10^5^ | 1.11×10^5^ | 9.49×10^4^ | 1.13×10^5^ |  | **TR** | *Cluster 1* | 1.02×10^5^ | 1.11×10^5^ | 9.68×10^4^ | 2.82×10^5^ |
|  |  | *Cluster 2* | 6.41×10^4^ | 6.95×10^4^ | 1.56×10^5^ | 1.85×10^5^ |  |  | *Cluster 2* | 6.41×10^4^ | 6.95×10^4^ | 1.59×10^5^ | 2.71×10^5^ |
|  |  | *Cluster 3* | 1.19×10^5^ | 1.25×10^5^ | 9.75×10^4^ | 1.16×10^5^ |  |  | *Cluster 3* | 1.19×10^5^ | 1.25×10^5^ | 9.94×10^4^ | 1.36×10^5^ |
|  |  | *Cluster 4* | 7.08×10^4^ | 7.63×10^4^ | 8.57×10^4^ | 1.02×10^5^ |  |  | *Cluster 4* | 7.08×10^4^ | 7.63×10^4^ | 8.73×10^4^ | 1.20×10^5^ |
|  |  | *Cluster 5* | 5.36×10^4^ | 5.54×10^4^ | 1.29×10^5^ | 1.53×10^5^ |  |  | *Cluster 5* | 5.36×10^4^ | 5.54×10^4^ | 1.32×10^5^ | 1.80×10^5^ |
|  |  | *Cluster 6* | 9.50×10^4^ | 1.02×10^5^ | 1.40×10^5^ | 1.67×10^5^ |  |  | *Cluster 6* | 9.50×10^4^ | 1.02×10^5^ | 1.43×10^5^ | 1.96×10^5^ |

| **Table S8.** The statistics of calculated climate indices (averaged FD, ID, SU, and TR) separately in each cluster of studied countries (except Kazakhstan) between 1962 and 2100 under both climate scenarios. | | | | | | | | | | | | | |
| --- | --- | --- | --- | --- | --- | --- | --- | --- | --- | --- | --- | --- | --- |
| **SPSS 2-4.5** |  | ***Kyrgyzstan*** | | | | | **SPSS 5-8.5** |  | ***Kyrgyzstan*** | | | | |
|  | **Climate index** | | **Historical 1** | **Historical 2** | **Near-future** | **Far-future** |  | **Climate index** | | **Historical 1** | **Historical 2** | **Near-future** | **Far-future** |
|  | **FD** | *Cluster 1* | 2.46×10^5^ | 2.14×10^5^ | 3.21×10^5^ | 2.63×10^5^ |  | **FD** | *Cluster 1* | 2.46×10^5^ | 2.14×10^5^ | 3.29×10^5^ | 8.85×10^4^ |
|  |  | *Cluster 2* | 2.91×10^5^ | 2.77×10^5^ | 1.95×10^5^ | 1.60×10^5^ |  |  | *Cluster 2* | 2.91×10^5^ | 2.77×10^5^ | 1.99×10^5^ | 6.68×10^4^ |
|  | **ID** | *Cluster 1* | 2.23×10^5^ | 1.89×10^5^ | 3.51×10^5^ | 3.14×10^5^ |  | **ID** | *Cluster 1* | 2.23×10^5^ | 1.89×10^5^ | 3.50×10^5^ | 1.59×10^5^ |
|  |  | *Cluster 2* | 2.77×10^5^ | 2.63×10^5^ | 2.09×10^5^ | 1.87×10^5^ |  |  | *Cluster 2* | 2.77×10^5^ | 2.63×10^5^ | 2.09×10^5^ | 1.16×10^5^ |
|  | **SU** | *Cluster 1* | 7.65×10^4^ | 7.96×10^4^ | 1.96×10^5^ | 2.72×10^5^ |  | **SU** | *Cluster 1* | 7.65×10^4^ | 7.96×10^4^ | 2.38×10^5^ | 2.60×10^5^ |
|  |  | *Cluster 2* | 2.12×10^3^ | 2.38×10^3^ | 1.15×10^5^ | 1.62×10^5^ |  |  | *Cluster 2* | 2.12×10^3^ | 2.38×10^3^ | 1.41×10^5^ | 1.86×10^5^ |
|  | **TR** | *Cluster 1* | 2.18×10^5^ | 2.30×10^5^ | 3.35×10^5^ | 3.99×10^5^ |  | **TR** | *Cluster 1* | 2.18×10^5^ | 2.30×10^5^ | 3.42×10^5^ | 3.54×10^5^ |
|  |  | *Cluster 2* | 1.05×10^4^ | 1.16×10^4^ | 1.98×10^5^ | 2.37×10^5^ |  |  | *Cluster 2* | 1.05×10^4^ | 1.16×10^4^ | 2.02×10^5^ | 2.51×10^5^ |
|  |  | ***Tajikistan*** | | | | |  |  | ***Tajikistan*** | | | | |
|  | **Climate index** | | **Historical 1** | **Historical 2** | **Near-future** | **Far-future** |  | **Climate index** | | **Historical 1** | **Historical 2** | **Near-future** | **Far-future** |
|  | **FD** | *Cluster 1* | 5.34×10^4^ | 4.29×10^4^ | 7.80×10^4^ | 6.37×10^4^ |  | **FD** | *Cluster 1* | 5.34×10^4^ | 4.29×10^4^ | 7.99×10^4^ | 1.76×10^4^ |
|  |  | *Cluster 2* | 2.76×10^4^ | 2.20×10^4^ | 4.84×10^4^ | 3.95×10^4^ |  |  | *Cluster 2* | 2.76×10^4^ | 2.20×10^4^ | 4.98×10^4^ | 1.90×10^4^ |
|  | **ID** | *Cluster 1* | 4.80×10^4^ | 3.75×10^4^ | 8.65×10^4^ | 7.73×10^4^ |  | **ID** | *Cluster 1* | 4.80×10^4^ | 3.75×10^4^ | 8.66×10^4^ | 3.32×10^4^ |
|  |  | *Cluster 2* | 2.49×10^4^ | 1.94×10^4^ | 5.37×10^4^ | 4.79×10^4^ |  |  | *Cluster 2* | 2.49×10^4^ | 1.94×10^4^ | 5.36×10^4^ | 3.54×10^4^ |
|  | **SU** | *Cluster 1* | 4.38×10^4^ | 5.00×10^4^ | 5.21×10^4^ | 7.11×10^4^ |  | **SU** | *Cluster 1* | 4.38×10^4^ | 5.00×10^4^ | 6.26×10^4^ | 5.41×10^4^ |
|  |  | *Cluster 2* | 2.62×10^4^ | 2.92×10^4^ | 3.11×10^4^ | 4.27×10^4^ |  |  | *Cluster 2* | 2.62×10^4^ | 2.92×10^4^ | 3.75×10^4^ | 5.76×10^4^ |
|  | **TR** | *Cluster 1* | 8.36×10^4^ | 9.14×10^4^ | 9.02×10^4^ | 1.06×10^5^ |  | **TR** | *Cluster 1* | 8.36×10^4^ | 9.14×10^4^ | 9.18×10^4^ | 7.33×10^4^ |
|  |  | *Cluster 2* | 4.79×10^4^ | 5.03×10^4^ | 5.26×10^4^ | 6.25×10^4^ |  |  | *Cluster 2* | 4.79×10^4^ | 5.03×10^4^ | 5.36×10^4^ | 7.79×10^4^ |
|  |  | ***Turkmenistan*** | | | | |  |  | ***Turkmenistan*** | | | | |
|  | **Climate index** | | **Historical 1** | **Historical 2** | **Near-future** | **Far-future** |  | **Climate index** | | **Historical 1** | **Historical 2** | **Near-future** | **Far-future** |
|  | **FD** | *Cluster 1* | 2.37×10^3^ | 1.34×10^3^ | 8.71×10^3^ | 7.13×10^3^ |  | **FD** | *Cluster 1* | 2.46×10^5^ | 2.14×10^5^ | 8.92×10^3^ | 3.84×10^3^ |
|  |  | *Cluster 2* | 1.73×10^3^ | 9.77×10^2^ | 1.20×10^4^ | 9.85×10^3^ |  |  | *Cluster 2* | 2.91×10^5^ | 2.77×10^5^ | 1.23×10^4^ | 1.06×10^3^ |
|  | **ID** | *Cluster 1* | 2.08×10^3^ | 1.12×10^3^ | 9.62×10^3^ | 8.61×10^3^ |  | **ID** | *Cluster 1* | 2.23×10^5^ | 1.89×10^5^ | 9.57×10^3^ | 6.71×10^3^ |
|  |  | *Cluster 2* | 1.52×10^3^ | 8.05×10^2^ | 1.32×10^4^ | 1.18×10^4^ |  |  | *Cluster 2* | 2.77×10^5^ | 2.63×10^5^ | 1.31×10^4^ | 1.99×10^3^ |
|  | **SU** | *Cluster 1* | 9.42×10^3^ | 1.05×10^4^ | 5.45×10^3^ | 7.50×10^3^ |  | **SU** | *Cluster 1* | 7.65×10^4^ | 7.96×10^4^ | 6.60×10^3^ | 9.33×10^3^ |
|  |  | *Cluster 2* | 1.15×10^4^ | 1.21×10^4^ | 7.09×10^3^ | 9.89×10^3^ |  |  | *Cluster 2* | 2.12×10^3^ | 2.38×10^3^ | 8.73×10^3^ | 3.13×10^3^ |
|  | **TR** | *Cluster 1* | 1.31×10^4^ | 1.40×10^4^ | 9.24×10^3^ | 1.10×10^4^ |  | **TR** | *Cluster 1* | 2.18×10^5^ | 2.30×10^5^ | 9.42×10^3^ | 1.26×10^4^ |
|  |  | *Cluster 2* | 1.49×10^4^ | 1.56×10^4^ | 1.22×10^4^ | 1.45×10^4^ |  |  | *Cluster 2* | 1.05×10^4^ | 1.16×10^4^ | 1.24×10^4^ | 4.26×10^3^ |
|  |  | ***Uzbekistan*** | | | | |  |  | ***Uzbekistan*** | | | | |
|  | **Climate index** | | **Historical 1** | **Historical 2** | **Near-future** | **Far-future** |  | **Climate index** | | **Historical 1** | **Historical 2** | **Near-future** | **Far-future** |
|  | **FD** | *Cluster 1* | 2.22×10^4^ | 1.77×10^4^ | 4.41×10^4^ | 4.51×10^4^ |  | **FD** | *Cluster 1* | 5.34×10^4^ | 4.29×10^4^ | 4.51×10^4^ | 2.17×10^4^ |
|  |  | *Cluster 2* | 5.82×10^3^ | 4.07×10^3^ | 2.03×10^4^ | 2.08×10^4^ |  |  | *Cluster 2* | 2.76×10^4^ | 2.20×10^4^ | 2.08×10^4^ | 2.88×10^3^ |
|  | **ID** | *Cluster 1* | 1.97×10^4^ | 1.48×10^4^ | 4.86×10^4^ | 4.84×10^4^ |  | **ID** | *Cluster 1* | 4.80×10^4^ | 3.75×10^4^ | 4.84×10^4^ | 3.94×10^4^ |
|  |  | *Cluster 2* | 5.12×10^3^ | 3.34×10^3^ | 2.24×10^4^ | 2.24×10^4^ |  |  | *Cluster 2* | 2.49×10^4^ | 1.94×10^4^ | 2.24×10^4^ | 5.57×10^3^ |
|  | **SU** | *Cluster 1* | 2.54×10^4^ | 2.75×10^4^ | 2.70×10^4^ | 3.29×10^4^ |  | **SU** | *Cluster 1* | 4.38×10^4^ | 5.00×10^4^ | 3.29×10^4^ | 5.98×10^4^ |
|  |  | *Cluster 2* | 1.62×10^4^ | 1.81×10^4^ | 1.28×10^4^ | 1.54×10^4^ |  |  | *Cluster 2* | 2.62×10^4^ | 2.92×10^4^ | 1.54×10^4^ | 9.56×10^3^ |
|  | **TR** | *Cluster 1* | 4.38×10^4^ | 4.59×10^4^ | 4.60×10^4^ | 4.69×10^4^ |  | **TR** | *Cluster 1* | 8.36×10^4^ | 9.14×10^4^ | 4.69×10^4^ | 8.10×10^4^ |
|  |  | *Cluster 2* | 2.44×10^4^ | 2.59×10^4^ | 2.16×10^4^ | 2.20×10^4^ |  |  | *Cluster 2* | 4.79×10^4^ | 5.03×10^4^ | 2.20×10^4^ | 1.30×10^4^ |


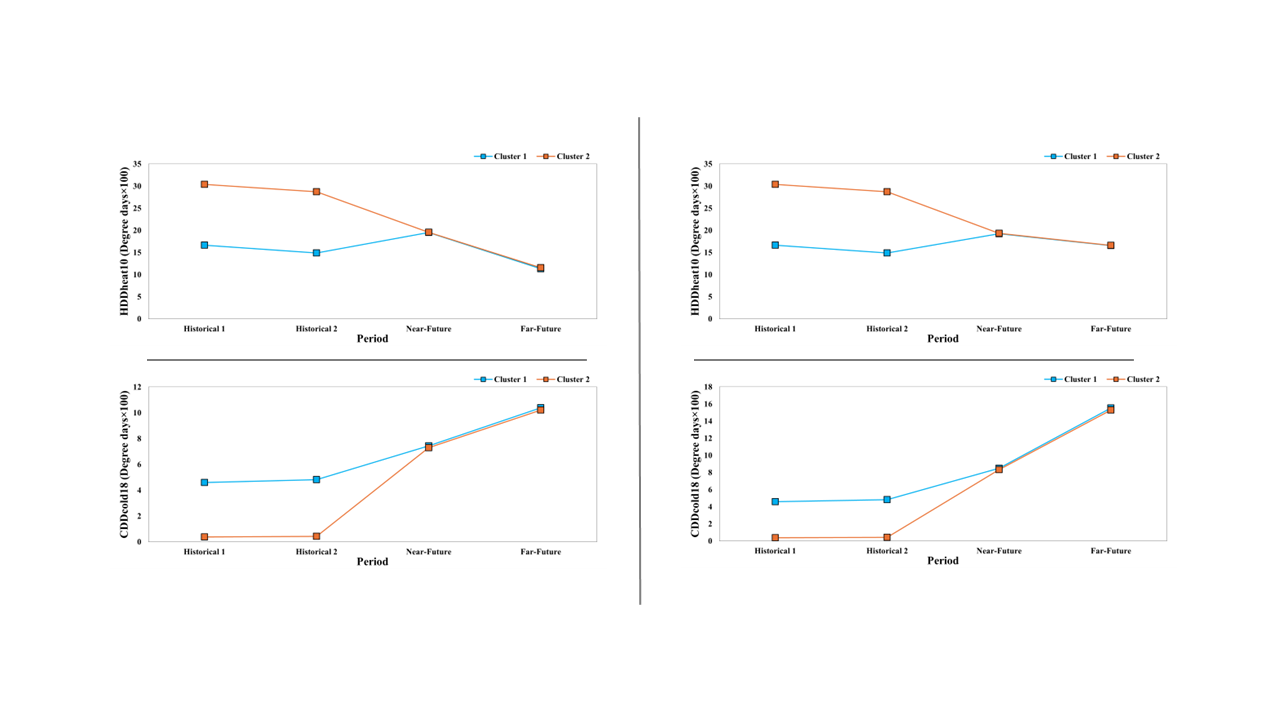


**Fig. S14.** The temporal changes in the statistics of averaged HDDheat10 and CDDcold18 separately in Kazakhstan between 1962 and 2100 under (**Left**) SSP2–4.5 and (**Right**) SSP5–8.5.


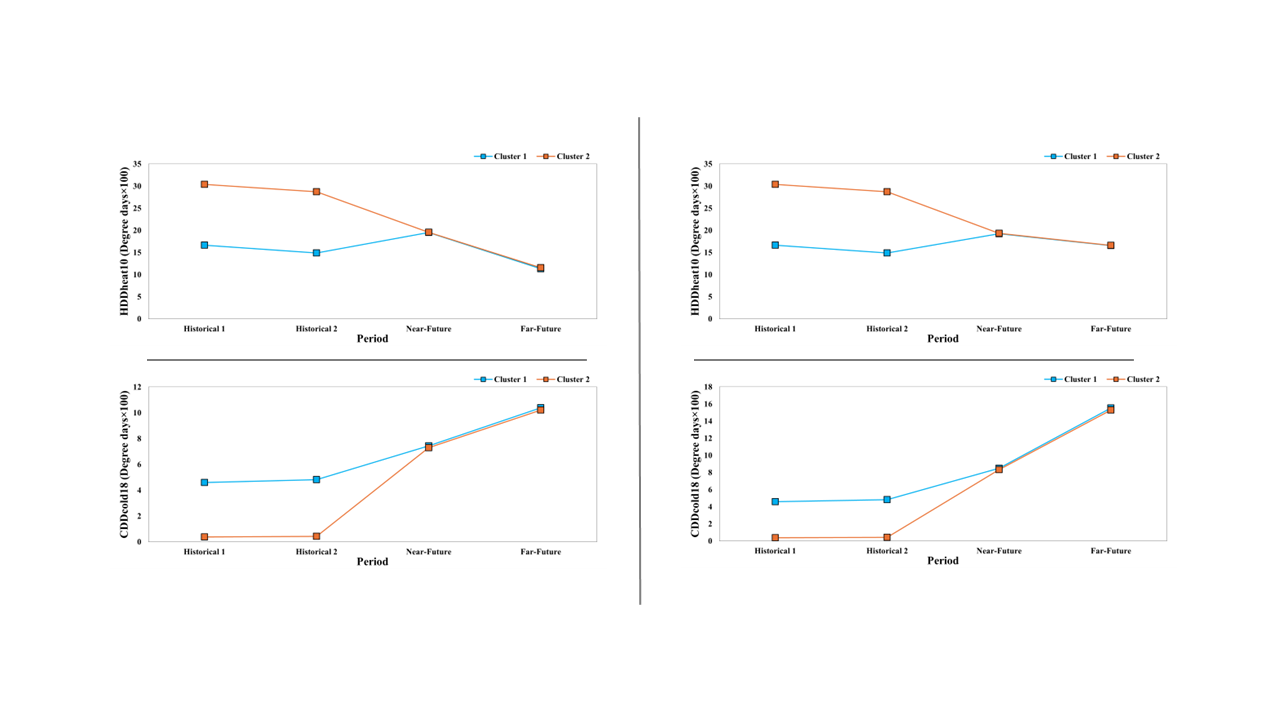
**Fig. S15.** The temporal changes in the statistics of averaged HDDheat10 and CDDcold18 separately in Kyrgystan between 1962 and 2100 under (**Left**) SSP2–4.5 and (**Right**) SSP5–8.5.


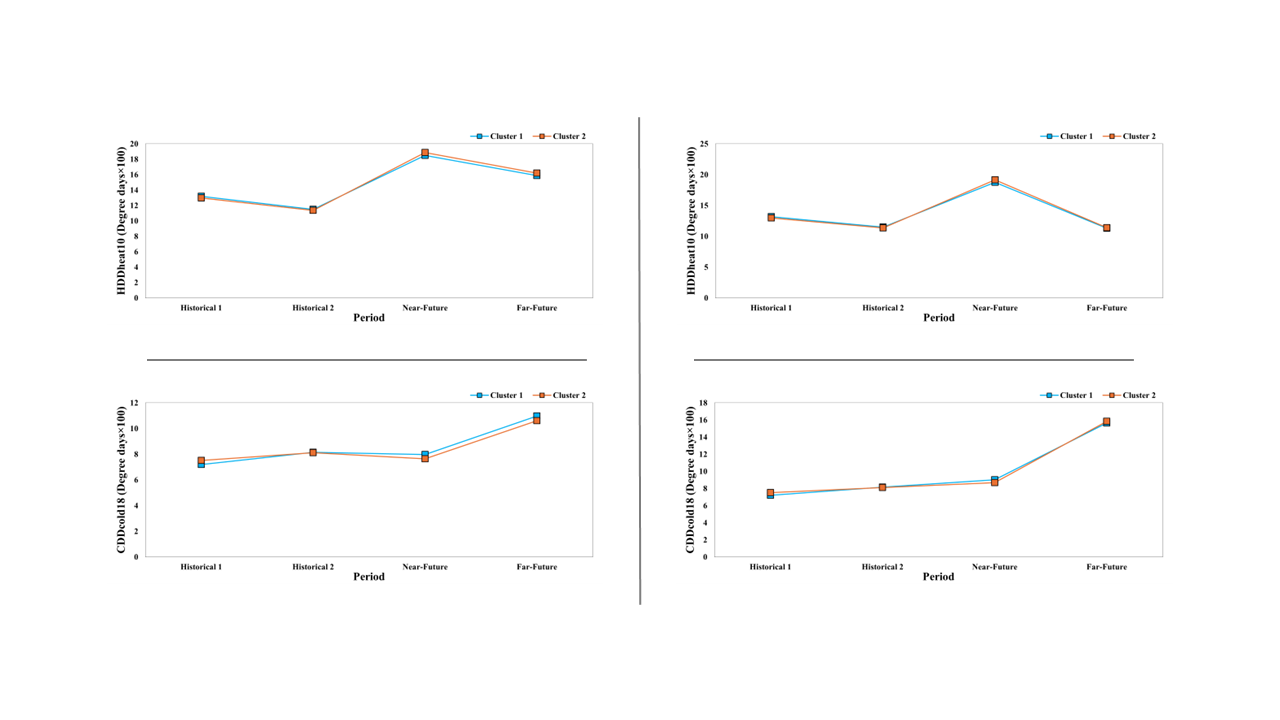


**Fig. S16.** The temporal changes in the statistics of averaged HDDheat10 and CDDcold18 separately in Tajikistan between 1962 and 2100 under (**Left**) SSP2–4.5 and (**Right**) SSP5–8.5.


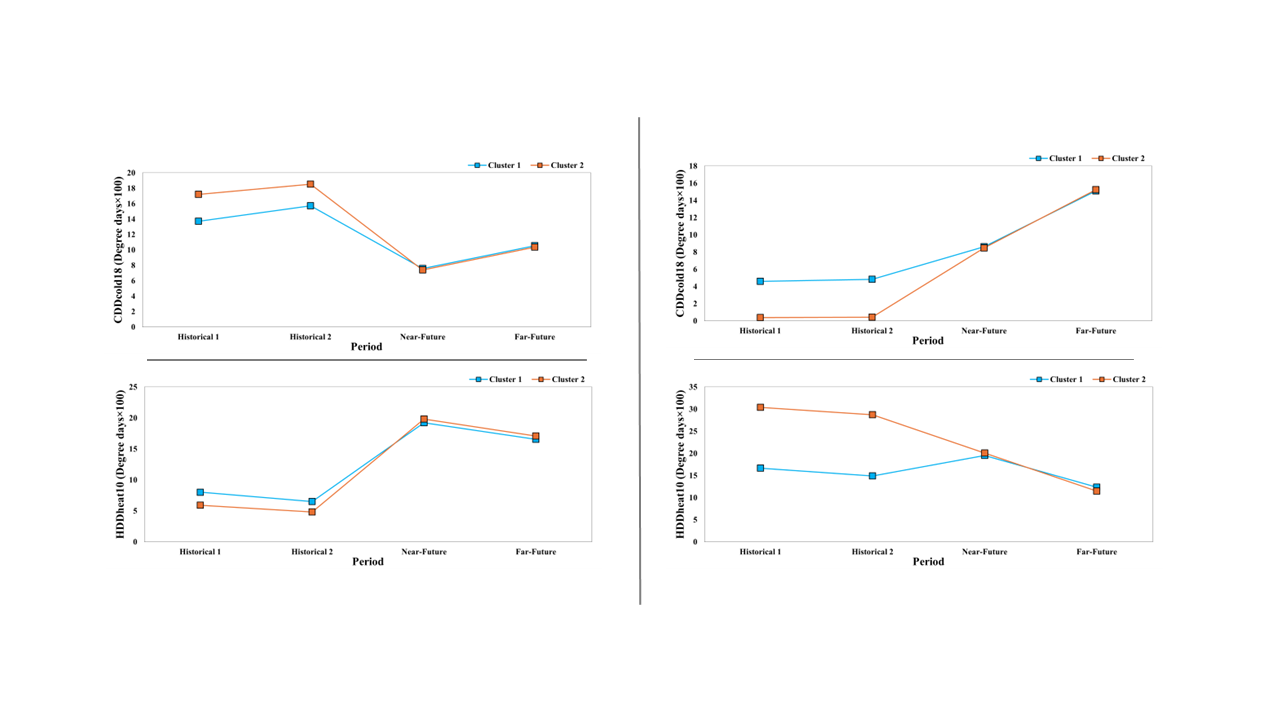


**Fig. S17.** The temporal changes in the statistics of averaged HDDheat10 and CDDcold18 separately in Turkmenistan between 1962 and 2100 under (**Left**) SSP2–4.5 and (**Right**) SSP5–8.5.


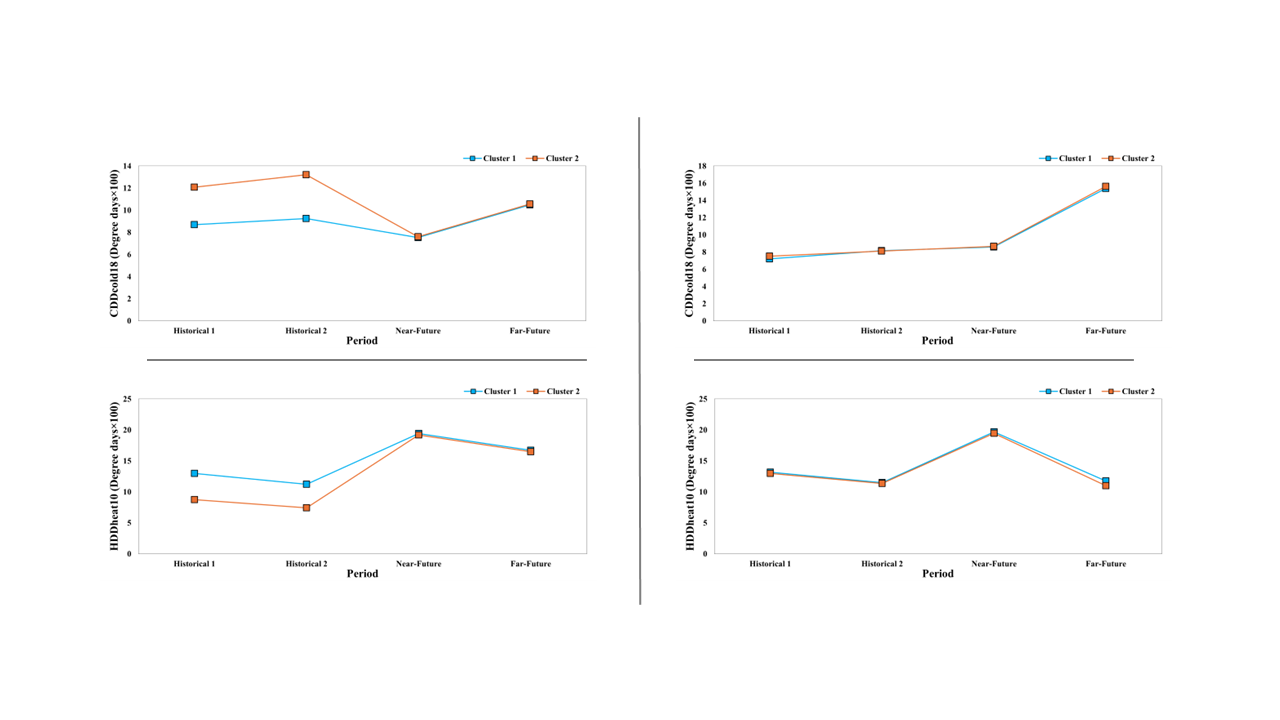


**Fig. S18.** The temporal changes in the statistics of averaged HDDheat10 and CDDcold18 separately in Uzbekistan between 1962 and 2100 under (**Left**) SSP2–4.5 and (**Right**) SSP5–8.5.

| **Table S9.** The statistics of calculated climate indices (averaged HDDheat10 and CDDcold18) separately in studied countries between 1962 and 2100 under both climate scenarios. | | | | | | | |
| --- | --- | --- | --- | --- | --- | --- | --- |
| **SSP2–4.5** | *Time Period* | ***Kazakhstan*** | | **SSP5–8.5** | *Time Period* | ***Kazakhstan*** | |
|  |  | **HDDheat10** | **CDDcold18** |  |  | **HDDheat10** | **CDDcold18** |
|  | *Historical 1* | 2773 | 503 |  | *Historical 1* | 2773 | 503 |
|  | *Historical 2* | 2530 | 564 |  | *Historical 2* | 2530 | 564 |
|  | *Near-future* | 1928 | 756 |  | *Near-future* | 1953 | 860 |
|  | *Far-future* | 1658 | 1052 |  | *Far-future* | 1167 | 1532 |
|  | *Time Period* | ***Kyrgyzstan*** | |  | *Time Period* | ***Kyrgyzstan*** | |
|  |  | **HDDheat10** | **CDDcold18** |  |  | **HDDheat10** | **CDDcold18** |
|  | *Historical 1* | 2163 | 303 |  | *Historical 1* | 2163 | 303 |
|  | *Historical 2* | 1993 | 318 |  | *Historical 2* | 1993 | 318 |
|  | *Near-future* | 1924 | 739 |  | *Near-future* | 1950 | 843 |
|  | *Far-future* | 1654 | 1033 |  | *Far-future* | 1140 | 1560 |
|  | *Time Period* | ***Tajikistan*** | |  | *Time Period* | ***Tajikistan*** | |
|  |  | **HDDheat10** | **CDDcold18** |  |  | **HDDheat10** | **CDDcold18** |
|  | *Historical 1* | 1584 | 660 |  | *Historical 1* | 1584 | 660 |
|  | *Historical 2* | 1416 | 739 |  | *Historical 2* | 1416 | 739 |
|  | *Near-future* | 1869 | 780 |  | *Near-future* | 1896 | 884 |
|  | *Far-future* | 1604 | 1078 |  | *Far-future* | 1141 | 1562 |
|  | *Time Period* | ***Turkmenistan*** | |  | *Time Period* | ***Turkmenistan*** | |
|  |  | **HDDheat10** | **CDDcold18** |  |  | **HDDheat10** | **CDDcold18** |
|  | *Historical 1* | 688 | 1590 |  | *Historical 1* | 688 | 1590 |
|  | *Historical 2* | 550 | 1746 |  | *Historical 2* | 550 | 1746 |
|  | *Near-future* | 1946 | 749 |  | *Near-future* | 1971 | 854 |
|  | *Far-future* | 1674 | 1044 |  | *Far-future* | 1171 | 1532 |
|  | *Time Period* | ***Uzbekistan*** | |  | *Time Period* | ***Uzbekistan*** | |
|  |  | **HDDheat10** | **CDDcold18** |  |  | **HDDheat10** | **CDDcold18** |
|  | *Historical 1* | 1098 | 1057 |  | *Historical 1* | 1098 | 1057 |
|  | *Historical 2* | 942 | 1132 |  | *Historical 2* | 942 | 1132 |
|  | *Near-future* | 1933 | 755 |  | *Near-future* | 1958 | 860 |
|  | *Far-future* | 1662 | 1051 |  | *Far-future* | 1167 | 1546 |

**Fig. S19.** The slope of spatial-temporal changes of (**1**) CDDcold18 and (**2**) HDDheat10 in CA, considering SSP2–4.5 and SSP5–8.5 climate projections between 1962 and 2100
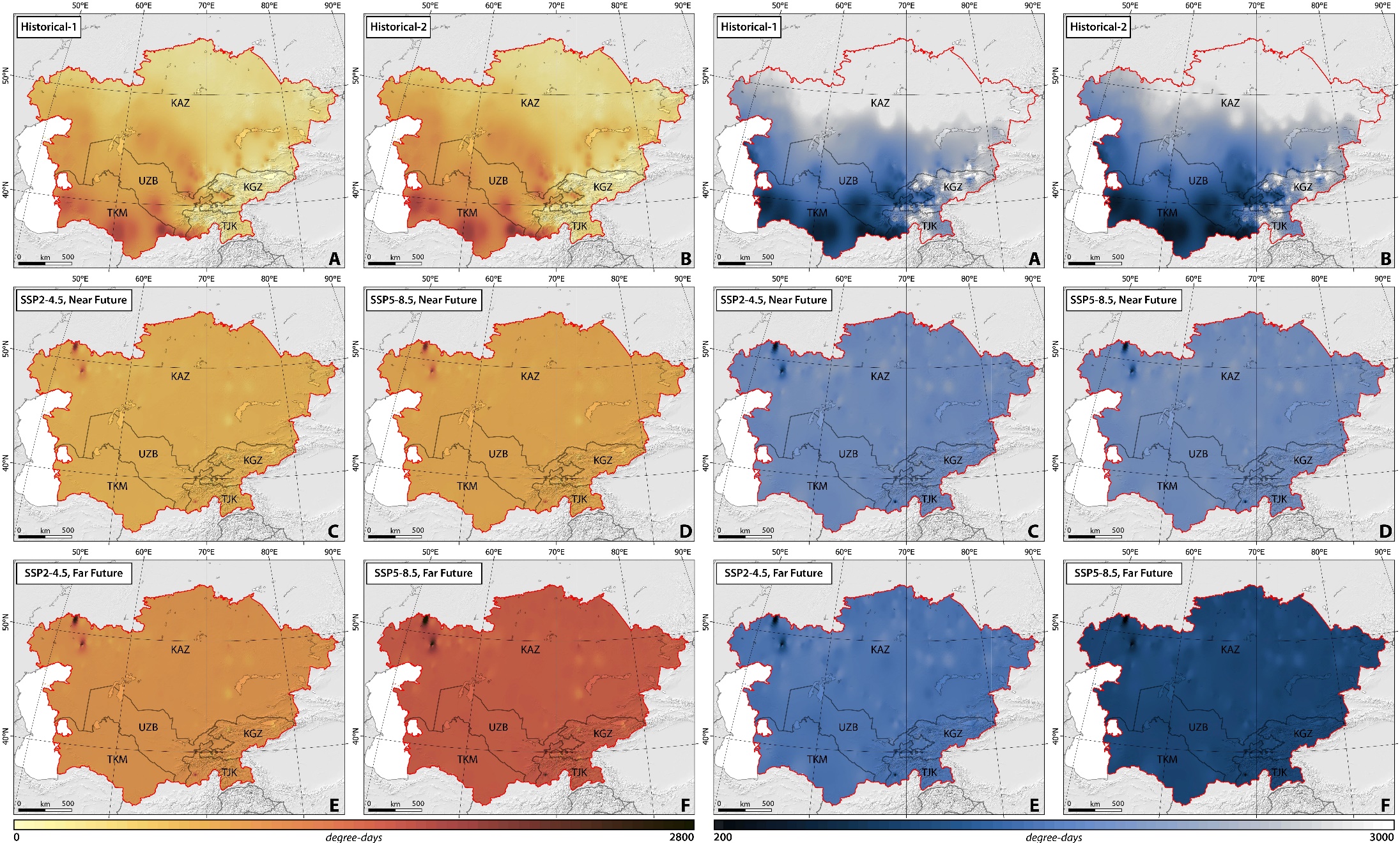
.

**1**

**2**

| **Table S10.** The statistics of calculated climate indices (averaged HDDheat10 and CDDcold18) separately in each cluster of studied countries between 1962 and 2100 under both climate scenarios. | | | | | | | | | | | | | |
| --- | --- | --- | --- | --- | --- | --- | --- | --- | --- | --- | --- | --- | --- |
| **SPSS 2-4.5** |  | ***Kazakhstan*** | | | | | **SPSS 5-8.5** |  | ***Kazakhstan*** | | | | |
|  | **Climate Index** | | **Historical 1** | **Historical 2** | **Near-future** | **Far-future** |  | **Climate Index** | | **Historical 1** | **Historical 2** | **Near-future** | **Far-future** |
|  | **HDDheat10** | *Cluster 1* | 1666 | 1440 | 1945 | 1673 |  | **HDDheat10** | *Cluster 1* | 1666 | 1440 | 1970 | 1172 |
|  |  | *Cluster 2* | 3557 | 3289 | 1945 | 1674 |  |  | *Cluster 2* | 3557 | 3289 | 1969 | 1180 |
|  |  | *Cluster 3* | 1959 | 1743 | 1950 | 1677 |  |  | *Cluster 3* | 1959 | 1743 | 1975 | 1178 |
|  |  | *Cluster 4* | 2377 | 2159 | 1894 | 1626 |  |  | *Cluster 4* | 2377 | 2159 | 1921 | 1134 |
|  |  | *Cluster 5* | 3638 | 3389 | 1932 | 1661 |  |  | *Cluster 5* | 3638 | 3389 | 1958 | 1162 |
|  |  | *Cluster 6* | 3056 | 2769 | 1972 | 1699 |  |  | *Cluster 6* | 3056 | 2769 | 1996 | 1196 |
|  | **CDDcold18** | *Cluster 1* | 861 | 986 | 748 | 1044 |  | **CDDcold18** | *Cluster 1* | 861 | 986 | 853 | 1541 |
|  |  | *Cluster 2* | 237 | 256 | 747 | 1042 |  |  | *Cluster 2* | 237 | 256 | 851 | 1518 |
|  |  | *Cluster 3* | 849 | 969 | 743 | 1038 |  |  | *Cluster 3* | 849 | 969 | 848 | 1535 |
|  |  | *Cluster 4* | 468 | 509 | 746 | 1041 |  |  | *Cluster 4* | 468 | 509 | 849 | 1540 |
|  |  | *Cluster 5* | 247 | 253 | 754 | 1050 |  |  | *Cluster 5* | 247 | 253 | 859 | 1549 |
|  |  | *Cluster 6* | 480 | 555 | 753 | 1047 |  |  | *Cluster 6* | 480 | 555 | 856 | 1538 |
|  |  | ***Kyrgyzstan*** | | | | |  |  | ***Kyrgyzstan*** | | | | |
|  | **Climate Index** | | **Historical 1** | **Historical 2** | **Near-future** | **Far-future** |  | **Climate Index** | | **Historical 1** | **Historical 2** | **Near-future** | **Far-future** |
|  | **HDDheat10** | *Cluster 1* | 1663 | 1489 | 1925 | 1654 |  | **HDDheat10** | *Cluster 1* | 1663 | 1489 | 1951 | 1132 |
|  |  | *Cluster 2* | 3037 | 2870 | 1931 | 1660 |  |  | *Cluster 2* | 3037 | 2870 | 1956 | 1154 |
|  | **CDDcold18** | *Cluster 1* | 460 | 482 | 744 | 1038 |  | **CDDcold18** | *Cluster 1* | 460 | 482 | 848 | 1553 |
|  |  | *Cluster 2* | 38 | 43 | 729 | 1021 |  |  | *Cluster 2* | 38 | 43 | 831 | 1530 |
|  |  | ***Tajikistan*** | | | | |  |  | ***Tajikistan*** | | | | |
|  | **Climate Index** | | **Historical 1** | **Historical 2** | **Near-future** | **Far-future** |  | **Climate Index** | | **Historical 1** | **Historical 2** | **Near-future** | **Far-future** |
|  | **HDDheat10** | *Cluster 1* | 1319 | 1149 | 1851 | 1589 |  | **HDDheat10** | *Cluster 1* | 1319 | 1149 | 1877 | 1131 |
|  |  | *Cluster 2* | 1298 | 1137 | 1888 | 1620 |  |  | *Cluster 2* | 1298 | 1137 | 1915 | 1139 |
|  | **CDDcold18** | *Cluster 1* | 720 | 815 | 798 | 1098 |  | **CDDcold18** | *Cluster 1* | 720 | 815 | 902 | 1565 |
|  |  | *Cluster 2* | 752 | 812 | 763 | 1061 |  |  | *Cluster 2* | 752 | 812 | 868 | 1585 |
|  |  | ***Turkmenistan*** | | | | |  |  | ***Turkmenistan*** | | | | |
|  | **Climate Index** | | **Historical 1** | **Historical 2** | **Near-future** | **Far-future** |  | **Climate Index** | | **Historical 1** | **Historical 2** | **Near-future** | **Far-future** |
|  | **HDDheat10** | *Cluster 1* | 801 | 651 | 1925 | 1654 |  | **HDDheat10** | *Cluster 1* | 1663 | 1489 | 1949 | 1236 |
|  |  | *Cluster 2* | 590 | 482 | 1931 | 1708 |  |  | *Cluster 2* | 3037 | 2870 | 2007 | 1146 |
|  | **CDDcold18** | *Cluster 1* | 1372 | 1573 | 744 | 1054 |  | **CDDcold18** | *Cluster 1* | 460 | 482 | 862 | 1511 |
|  |  | *Cluster 2* | 1721 | 1852 | 729 | 1035 |  |  | *Cluster 2* | 38 | 43 | 846 | 1525 |
|  |  | ***Uzbekistan*** | | | | |  |  | ***Uzbekistan*** | | | | |
|  | **Climate Index** | | **Historical 1** | **Historical 2** | **Near-future** | **Far-future** |  | **Climate Index** | | **Historical 1** | **Historical 2** | **Near-future** | **Far-future** |
|  | **HDDheat10** | *Cluster 1* | 1297 | 1123 | 1851 | 1672 |  | **HDDheat10** | *Cluster 1* | 1319 | 1149 | 1969 | 1176 |
|  |  | *Cluster 2* | 876 | 743 | 1888 | 1649 |  |  | *Cluster 2* | 1298 | 1137 | 1945 | 1099 |
|  | **CDDcold18** | *Cluster 1* | 870 | 926 | 798 | 1049 |  | **CDDcold18** | *Cluster 1* | 720 | 815 | 858 | 1540 |
|  |  | *Cluster 2* | 1208 | 1321 | 763 | 1057 |  |  | *Cluster 2* | 752 | 812 | 865 | 1562 |

**
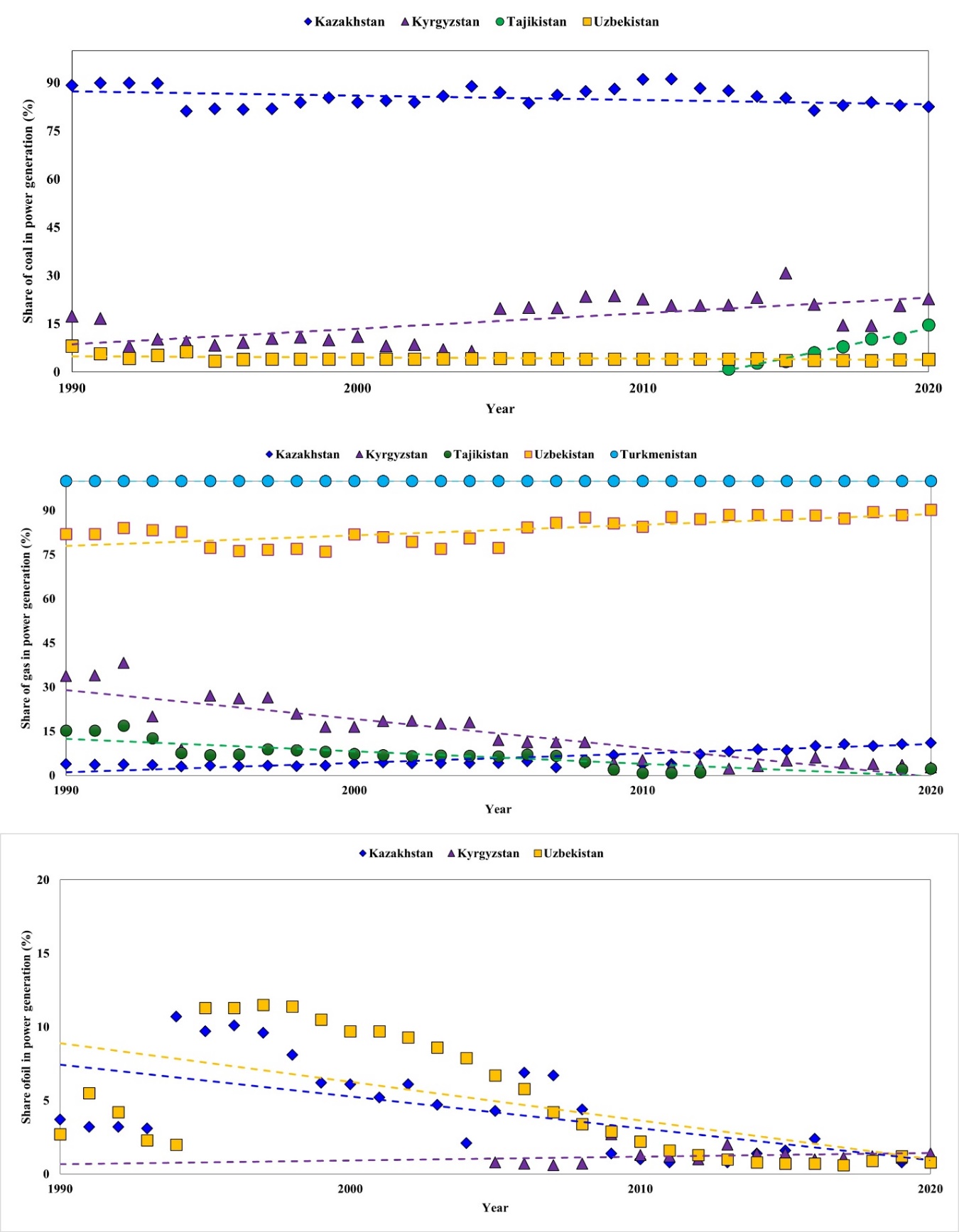
**

**C**

**B**

**A**

**Fig. S20.** The temporal changes in energy transition indicators of (**A**) Share of coal in power generation (%), (**B**) Share of gas in power generation (%), and (**C**) Share of oil in power generation (%) in CA between 1990 and 2021.


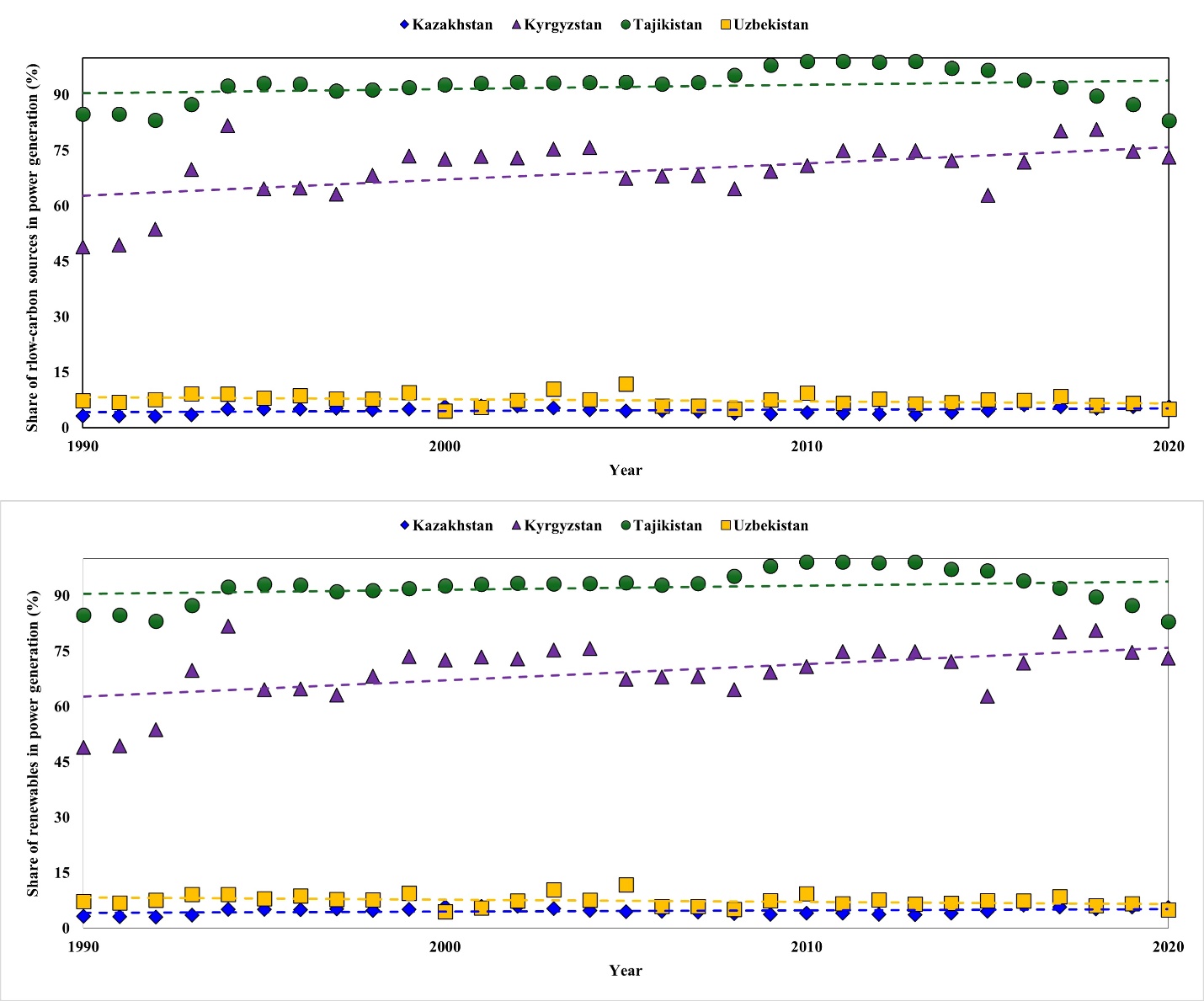


**B**

**A**

**Fig. S21.** The temporal changes in energy transition indicators of (**A**) Share of low-carbon sources in power generation (%) and (**B**) Share of renewables in power generation in CA between 1990 and 2021.

| **Table S11.** The statistics of heating-cooling energy generation separately in the studied countries in the near-far future under both climate scenarios. | | | |
| --- | --- | --- | --- |
| **SSP2–4.5** | | | |
| **Period** | **Kazakhstan** | | |
|  | ***Heating energy generation (kWh)*** | ***Cooling energy generation (kWh)*** | ***Total energy generation (kWh)*** |
| **Near-future** | 46 | 18 | 64 |
| **Far-future** | 40 | 25 | 65 |
| **Period** | **Kyrgyzstan** | | |
|  | ***Heating energy generation (kWh)*** | ***Cooling energy generation (kWh)*** | ***Total energy generation (kWh)*** |
| **Near-future** | 46 | 18 | 64 |
| **Far-future** | 40 | 25 | 64 |
| **Period** | **Tajikistan** | | |
|  | ***Heating energy generation (kWh)*** | ***Cooling energy generation (kWh)*** | ***Total energy generation (kWh)*** |
| **Near-future** | 45 | 19 | 64 |
| **Far-future** | 39 | 26 | 64 |
| **Period** | **Turkmenistan** | | |
|  | ***Heating energy generation (kWh)*** | ***Cooling energy generation (kWh)*** | ***Total energy generation (kWh)*** |
| **Near-future** | 47 | 18 | 65 |
| **Far-future** | 40 | 25 | 65 |
| **Period** | **Uzbekistan** | | |
|  | ***Heating energy generation (kWh)*** | ***Cooling energy generation (kWh)*** | ***Total energy generation (kWh)*** |
| **Near-future** | 46 | 18 | 65 |
| **Far-future** | 40 | 25 | 65 |
| **SSP5–8.5** | | | |
| **Period** | **Kazakhstan** | | |
|  | ***Heating energy generation (kWh)*** | ***Cooling energy generation (kWh)*** | ***Total energy generation (kWh)*** |
| **Near-future** | 47 | 21 | 68 |
| **Far-future** | 28 | 37 | 65 |
| **Period** | **Kyrgyzstan** | | |
|  | ***Heating energy generation (kWh)*** | ***Cooling energy generation (kWh)*** | ***Total energy generation (kWh)*** |
| **Near-future** | 47 | 20 | 67 |
| **Far-future** | 27 | 37 | 65 |
| **Period** | **Tajikistan** | | |
|  | ***Heating energy generation (kWh)*** | ***Cooling energy generation (kWh)*** | ***Total energy generation (kWh)*** |
| **Near-future** | 45 | 21 | 67 |
| **Far-future** | 27 | 37 | 65 |
| **Period** | **Turkmenistan** | | |
|  | ***Heating energy generation (kWh)*** | ***Cooling energy generation (kWh)*** | ***Total energy generation (kWh)*** |
| **Near-future** | 47 | 20 | 68 |
| **Far-future** | 28 | 37 | 65 |
| **Period** | **Uzbekistan** | | |
|  | ***Heating energy generation (kWh)*** | ***Cooling energy generation (kWh)*** | ***Total energy generation (kWh)*** |
| **Near-future** | 47 | 21 | 68 |
| **Far-future** | 28 | 37 | 65 |

| **Table S12.** The statistics of CO_2_ emission for heating-cooling purposes (tonne CO_2_/kWh) separately in the studied countries in the near-far future under both climate scenarios. | | | |
| --- | --- | --- | --- |
| **SSP2–4.5** | | | |
| Kazakhstan | | | |
| **Period** | ***CO_2_ emission for heating (tonne CO_2_/kWh)*** | ***CO_2_ emission for cooling (tonne CO_2_/kWh)*** | ***Total CO_2_ emission (tonne CO_2_/kWh)*** |
| **Near-future** | 3.80×10^-2^ | 1.49×10^-2^ | 5.29×10^-2^ |
| **Far-future** | 3.27×10^-2^ | 2.07×10^-2^ | 5.34×10^-2^ |
| **Kyrgyzstan** | | | |
| **Period** | ***CO_2_ emission for heating (tonne CO_2_/kWh)*** | ***CO_2_ emission for cooling (tonne CO_2_/kWh)*** | ***Total CO_2_ emission (tonne CO_2_/kWh)*** |
| **Near-future** | 6.45×10^-3^ | 2.48×10^-3^ | 8.93×10^-3^ |
| **Far-future** | 5.55×10^-3^ | 3.46×10^-3^ | 9.01×10^-3^ |
| **Tajikistan** | | | |
| **Period** | ***CO_2_ emission for heating (tonne CO_2_/kWh)*** | ***CO_2_ emission for cooling (tonne CO_2_/kWh)*** | ***Total CO_2_ emission (tonne CO_2_/kWh)*** |
| **Near-future** | 4.39×10^-3^ | 1.83×10^-3^ | 6.22×10^-3^ |
| **Far-future** | 3.77×10^-3^ | 2.53×10^-3^ | 6.30×10^-3^ |
| **Turkmenistan** | | | |
| **Period** | ***CO_2_ emission for heating (tonne CO_2_/kWh)*** | ***CO_2_ emission for cooling (tonne CO_2_/kWh)*** | ***Total CO_2_ emission (tonne CO_2_/kWh)*** |
| **Near-future** | 4.06×10^-2^ | 1.56×10^-2^ | 5.62×10^-2^ |
| **Far-future** | 3.49×10^-2^ | 2.18×10^-2^ | 5.67×10^-2^ |
| **Uzbekistan** | | | |
| **Period** | ***CO_2_ emission for heating (tonne CO_2_/kWh)*** | ***CO_2_ emission for cooling (tonne CO_2_/kWh)*** | ***Total CO_2_ emission (tonne CO_2_/kWh)*** |
| **Near-future** | 3.74×10^-2^ | 1.46×10^-2^ | 5.20×10^-2^ |
| **Far-future** | 3.22×10^-2^ | 2.03×10^-2^ | 5.25×10^-2^ |
| **SSP5–8.5** | | | |
| **Kazakhstan** | | | |
| **Period** | ***CO_2_ emission for heating (tonne CO_2_/kWh)*** | ***CO_2_ emission for cooling (tonne CO_2_/kWh)*** | ***Total CO_2_ emission (tonne CO_2_/kWh)*** |
| **Near-future** | 3.85×10^-2^ | 1.69×10^-2^ | 5.54×10^-2^ |
| **Far-future** | 2.30×10^-2^ | 3.02×10^-2^ | 5.32×10^-2^ |
| **Kyrgyzstan** | | | |
| **Period** | ***CO_2_ emission for heating (tonne CO_2_/kWh)*** | ***CO_2_ emission for cooling (tonne CO_2_/kWh)*** | ***Total CO_2_ emission (tonne CO_2_/kWh)*** |
| **Near-future** | 6.54×10^-3^ | 2.82×10^-3^ | 9.36×10^-3^ |
| **Far-future** | 3.82×10^-3^ | 5.23×10^-3^ | 9.05×10^-3^ |
| **Tajikistan** | | | |
| **Period** | ***CO_2_ emission for heating (tonne CO_2_/kWh)*** | ***CO_2_ emission for cooling (tonne CO_2_/kWh)*** | ***Total CO_2_ emission (tonne CO_2_/kWh)*** |
| **Near-future** | 4.45×10^-3^ | 2.07×10^-3^ | 6.53×10^-3^ |
| **Far-future** | 2.68×10^-3^ | 3.67×10^-3^ | 6.35×10^-3^ |
| **Turkmenistan** | | | |
| **Period** | ***CO_2_ emission for heating (tonne CO_2_/kWh)*** | ***CO_2_ emission for cooling (tonne CO_2_/kWh)*** | ***Total CO_2_ emission (tonne CO_2_/kWh)*** |
| **Near-future** | 4.11×10^-2^ | 1.78×10^-2^ | 5.89×10^-2^ |
| **Far-future** | 2.44×10^-2^ | 3.19×10^-2^ | 5.64×10^-2^ |
| **Uzbekistan** | | | |
| **Period** | ***CO_2_ emission for heating (tonne CO_2_/kWh)*** | ***CO_2_ emission for cooling (tonne CO_2_/kWh)*** | ***Total CO_2_ emission (tonne CO_2_/kWh)*** |
| **Near-future** | 3.79×10^-2^ | 1.66×10^-2^ | 5.45×10^-2^ |
| **Far-future** | 2.26×10^-2^ | 2.99×10^-2^ | 5.25×10^-2^ |
